# Protocell Computing on Aragonite Substrates

**DOI:** 10.1021/acsomega.5c09786

**Published:** 2026-01-13

**Authors:** Panagiotis Mougkogiannis, Andrew Adamatzky

**Affiliations:** Unconventional Computing Laboratory, 1981 University of the West of England, Bristol BS16 1QY, U.K.

## Abstract

Aragonite-proteinoid microstructures are an emerging type of biocomputing material. They mix inorganic calcium carbonate with self-assembled organic proteinoid networks. Scanning electron microscopy shows a range of structures. These include isolated microspheres and complex networks over 50 μm. They have dendritic shapes, with uneven nodes that create linear patterns resembling simple network topologies. Electrochemical testing shows a threshold response. This allows for all seven basic Boolean logic operations: AND, OR, NOT, NAND, NOR, XOR, and XNOR. It does this by classifying analog signals into binary states. This suggests a promising future for material-based computation. Frequency-dependent square wave voltammetry shows power-law scaling. It performs best in the 30–50 Hz range, which is important for biological use. This indicates adjustable electrochemical properties that are ideal for bioelectronic applications. The systems show autonomous oscillatory behavior for over 25 h. They maintain a steady ultralow frequency, like biological rhythms. This means they generate signals on their own, without any outside help. Impedance spectroscopy shows stable circuit features. There are strong links between resistive and capacitive parts. However, cyclic voltammetry shows that electrochemical degradation increases over time. These findings show that aragonite-proteinoid microstructures are well-suited for novel computing uses. They can help with things like autonomous sensing, neuromorphic devices, and biohybrid electronics. These microstructures use mineral-organic interfaces for processing information and generating signals. This approach connects synthetic materials to biological computing principles.

## Graphical abstract

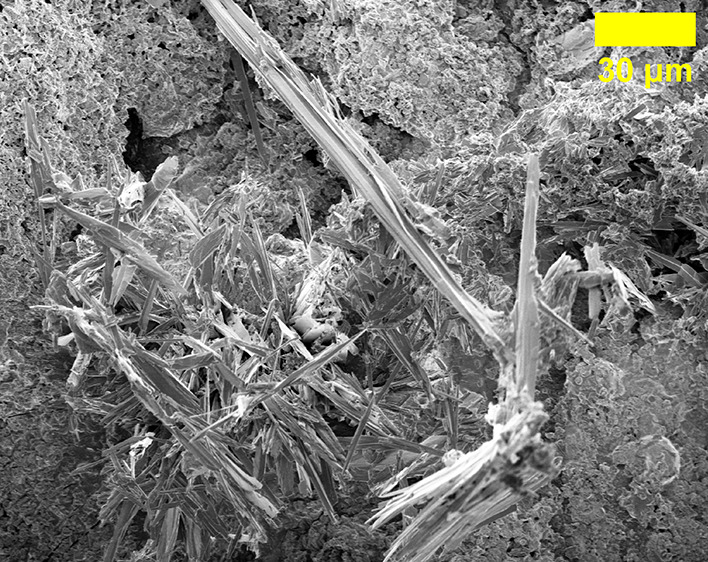

## Introduction

Digital technology is growing fast, pushing silicon-based computing to its limits and creating an urgent need for alternative computational approaches. Unconventional computing explores new ways to process information^1–4^ beyond conventional binary logic gates and electronic circuits. Silicon-based processors have dominated computing for decades, but their performance gains are now limited by the breakdown of Moore’s Law, thermal constraints, and rising energy demands.^5^ Recent research has identified several promising directions to overcome these limitations, including new materials for optical and neuromorphic computing systems,^5^ memristive devices that mimic neuronal activity,^6,7^ and magnetic skyrmions as information carriers in nonvon Neumann architectures.^8^ These unconventional strategies represent a shift toward “intelligent matter”—materials and systems that exhibit brain-like behavior, environmental adaptability, and distributed processing.^9^ Bioinspired approaches are particularly appealing because they harness the natural information processing capabilities of living systems, including the parallel processing of neural networks, cellular adaptation, and self-organizing properties of biological materials. Hybrid organic–inorganic systems offer significant promise for biocomputing advancement, processing information through electrochemical interactions and enabling applications in autonomous sensing, adaptive computation, and energy harvesting where traditional silicon-based technologies face fundamental challenges.

Proteinoids are protein-like polymers synthesized by thermal polymerization of amino acids, originally discovered by Sidney Fox.^10^ These materials self-assemble into microspheres that resemble cellular structures and serve as protocell models in prebiotic evolution studies. Beyond their origins research applications, proteinoids exhibit electrical excitability that enables neuromorphic computation, including electrochemical coupling responses, adaptability, and signal processing capabilities.^11^ Proteinoid microspheres demonstrate unique electrical responses to diverse stimuli—including sound^12–14^ and light^15,16^ —suggesting applications in bioinspired sensing and unconventional logic gates.^17^

Mineral-organic interfaces combine inorganic minerals with organic compounds to create hybrid materials with emergent properties exceeding those of individual components.^18^ These interfaces occur naturally in biomineralization processes such as shell and bone formation,^19^ where organic matrices guide mineral assembly to create structures with unique mechanical and functional properties.^20,21^ In unconventional computing, mineral-organic interfaces enable information processing through combined electronic and ionic transport, reversible electrochemical switching, and stable environments for signal transduction.^22^

Aragonite, an orthorhombic polymorph of calcium carbonate (CaCO_3_), possesses unique surface properties that guide organic self-assembly.^23–26^ Its surface chemistry features polar carbonate groups and positively charged calcium sites, creating an electrostatically diverse environment that interacts effectively with charged amino acids and peptide chains through electrostatic forces, hydrogen bonding, and coordination chemistry.^27,28^ Aragonite’s moderate surface reactivity, crystalline anisotropy promoting directional growth,^29^ and biocompatibility make it particularly suitable for stable organic–inorganic interfaces. Its natural occurrence in mollusk shells and coral skeletons demonstrates successful organic–inorganic integration,^30,31^ suggesting that aragonite-proteinoid systems may harness biomimetic principles for enhanced computational functionality.

Electrochemical computing utilizes ionic and electronic transport for information processing, storage, and logic operations,^32^ analogous to biological neural systems. Information propagates through changes in ionic concentrations, redox states, and electrical potentials, enabling analog signal processing^33^ and threshold-dependent logic operations. The time-dependent nature of electrochemical processes provides natural pathways for adaptive learning and memory formation, making these principles relevant for brain-inspired technologies.^34–36^

Biomimetic neural networks aim to replicate biological nervous system information processing through distributed parallel processing, adaptive connections, and dynamic signal integration.^37^ Key features include electrical excitability for signal propagation,^38^ oscillatory behavior for network synchronization, and adaptive responses through synaptic plasticity for learning and memory.^39^ Synthetic materials that exhibit electrical excitability, generate oscillations through feedback loops, and adapt their conductivity or structure based on activity—including conducting polymers,^40^ hybrid organic–inorganic systems, and self-organizing materials—may enable neuromorphic computing systems combining biological energy efficiency with engineered controllability and scalability.

Previous research has demonstrated biocomputing capabilities in proteinoid-based systems, including electrical excitability, electrochemical coupling responses, and basic information processing,^41^ while mineral-organic computing studies have explored inorganic materials that guide organic self-assembly and enhance electrochemical properties.^42^ However, most proteinoid computing studies employed substrates like glass or silicon that provide minimal structural guidance, resulting in random networks with limited connectivity,^28^ while mineral-organic research primarily utilized materials (silica, gold, titanium oxide) lacking optimal biocompatibility and surface chemistry for protein-mineral interactions. This gap leaves unexplored how calcium carbonate polymorphs, particularly aragonite, can influence proteinoid self-assembly to create complex, neural network-like structures with enhanced computational capabilities.^43–4^ Furthermore, no comprehensive evaluation exists of mineral-guided proteinoid networks performing multiple functions—logic processing, autonomous oscillations, and adaptive responses—within a single integrated platform.

This research explores aragonite-proteinoid microstructures as a new biocomputing platform. It has four main goals: (1) Structural Characterization: We will use scanning electron microscopy to study how proteinoids assemble on aragonite. This involves tracking their growth from simple microspheres to complex, dendritic networks. We also identify features that enhance connectivity and signal flow. (2) Electrochemical Analysis: We will find the best operating settings by looking at pulse amplitude effects. We will use frequency-dependent square wave voltammetry and conduct impedance spectroscopy. This will help us understand circuit behavior and long-term stability. (3) Boolean Logic Implementation: We will convert analog electrochemical signals into digital logic operations. We aim to confirm the system’s ability to perform seven basic Boolean gates (AND, OR, NOT, NAND, NOR, XOR, XNOR) and set the threshold conditions for reliable logic switching. (4) Autonomous Oscillatory Behavior Assessment: We will monitor spontaneous potential oscillations to characterize the periodicity, amplitude, and stability of self-sustained electrical activity. We will also explore how these systems can work as autonomous bioelectronic oscillators. This study looks at interactions from nanoscale to macroscale. It aims to see how aragonite substrates affect proteinoid self-assembly and biocomputing structures. We focus on key principles of aragonite-proteinoid systems. These systems can serve as unique platforms for computing that connect biological and synthetic information processing.

## Methods and Materials

### Preparation of Glutamic Acid-Aspartic Acid-Phenylalanine (Glu-Asp-Phe) Proteinoid

To synthesize the glutamic acid–aspartic acid–phenylalanine (Glu–Asp–Phe) proteinoid, we began with high-purity amino acid powders. We used a molar ratio of 2:1:1 (Glu:Asp:Phe), selected to enhance acidic characteristics and promote microsphere formation. We first weighed approximately 2.5 g of l-glutamic acid (0.017 mol) and transferred it into a round-bottom flask or other heat-resistant glass vessel. This step was conducted under an inert nitrogen atmosphere to minimize oxidative degradation. We then added 1.13 g of l-aspartic acid (0.0085 mol) and 1.40 g of l-phenylalanine (0.0085 mol) to the same vessel. Using a glass rod, we manually mixed the dry components until a uniform blend was achieved. We gently preheated the mixture at a temperature range of 100–120 °C for approximately 30 min, stirring intermittently to initiate partial dehydration and prevent clumping. This protocol is based on prior evidence that glutamic acid forms pyroglutamic acid intermediates upon heating. These intermediates facilitate chain elongation and enhance the efficiency of thermal polymerization reactions.^50^

After preheating, we placed the flask in an oil bath or heating mantle set to 170–200 °C. We stirred the mixture continuously for 4–6 h. This helped with thermal condensation and peptide bond formation. In the end, we got a thick, brownish melt, showing that proteinoids were synthesized. The reaction was monitored for the evolution of water vapor, and upon completion, the melt was cooled to room temperature, yielding a brittle solid. The crude proteinoid was dissolved in distilled water or a mild alkaline solution, like 0.1 M NaOH. This created a suspension. Then, it was centrifuged at 5000 rpm for 10 min to remove insoluble residues. The supernatant was dialyzed with deionized water using a 3–5 kDa cutoff membrane for 48 h. This step removed unreacted monomers and low-molecular-weight oligomers. Then, it was lyophilized to get a flocculent powder of purified Glu-Phe-Asp proteinoid. Now, it is ready for characterization and microsphere assembly in water.

### Electrophysiological Characterization

We used a PicoLog ADC-24 data logger to measure spontaneous oscillations in the aragonite–proteinoid system. The device was configured with a sampling rate of 1 Hz to capture low-frequency electrical fluctuations over extended durations. This setting enabled high-resolution monitoring of signal dynamics without overwhelming data storage capacity, allowing for continuous acquisition for up to 25 h. The ADC-24 provided precise analog-to-digital conversion, offering reliable baseline-subtracted potential measurements in millivolts. These readings facilitated the detection of signal peaks using Python-based analysis tools, such as the find_peaks function from the SciPy library. This approach proved effective for identifying subtle, spontaneous spikes indicative of neuron-reminiscent branched morphology activity, offering valuable insight into the system’s limit cycle dynamics and phase space trajectories.

All oscillation measurements were performed in triplicate (*n* = 3) using independently prepared aragonite–proteinoid samples under identical conditions (25 °C ± 1 °C, 50% ± 5% RH). Peak detection employed the SciPy find_peaks function with minimum distance parameter *d* = 10 samples and minimum prominence threshold of 5 mV to exclude noise artifacts. Statistical significance was assessed using one-way ANOVA with posthoc Tukey HSD test (α = 0.05). Oscillation periodicity values are reported as mean ± standard error of the mean (SEM), with 95% confidence intervals calculated using Student’s *t*-distribution.

For impedance spectroscopy, we employed a PalmSens potentiostat to characterize the frequency-dependent electrochemical properties of the proteinoid–aragonite interfaces. Measurements were conducted using direct current (DC) scan mode (*i*_dc_), with a time scan of 0.0 s and a total runtime of 20,000.0 s. The scan interval was set to 0.1 s, and the alternating current (AC) amplitude (*i*_ac_) was maintained at 0.01 (range, instrument-defined). The frequency scan incorporated 70 logarithmically spaced frequency points at a density of 9.9 per decade, ranging from a maximum frequency of 100,000.0 Hz downward. The full scan duration was approximately 5 h and 33 min, generating 1400 data points.

This setup enabled detailed analysis of both resistive and capacitive components in the system. It revealed time-dependent changes in complex impedance, which are essential for modeling equivalent circuits and assessing the long-term electrochemical stability of the hybrid biointerfaces. The experimental platform shown in Figure 1 uses several analytical techniques. It helps us understand the biocomputing properties of aragonite-proteinoid microstructures. We do this through real-time monitoring and multiscale analysis. The electrochemical analyzer is the main control unit. It coordinates measurements like differential pulse voltammetry, square wave voltammetry, and impedance spectroscopy within the set parameter ranges. The three-electrode cell setup has aragonite-proteinoid microstructures on the working electrode. This design allows for accurate electrochemical testing. It also keeps environmental conditions stable with built-in temperature and humidity control systems. This setup lets us study pulse amplitude effects, frequency responses, and long-term stability.

**Figure 1:**
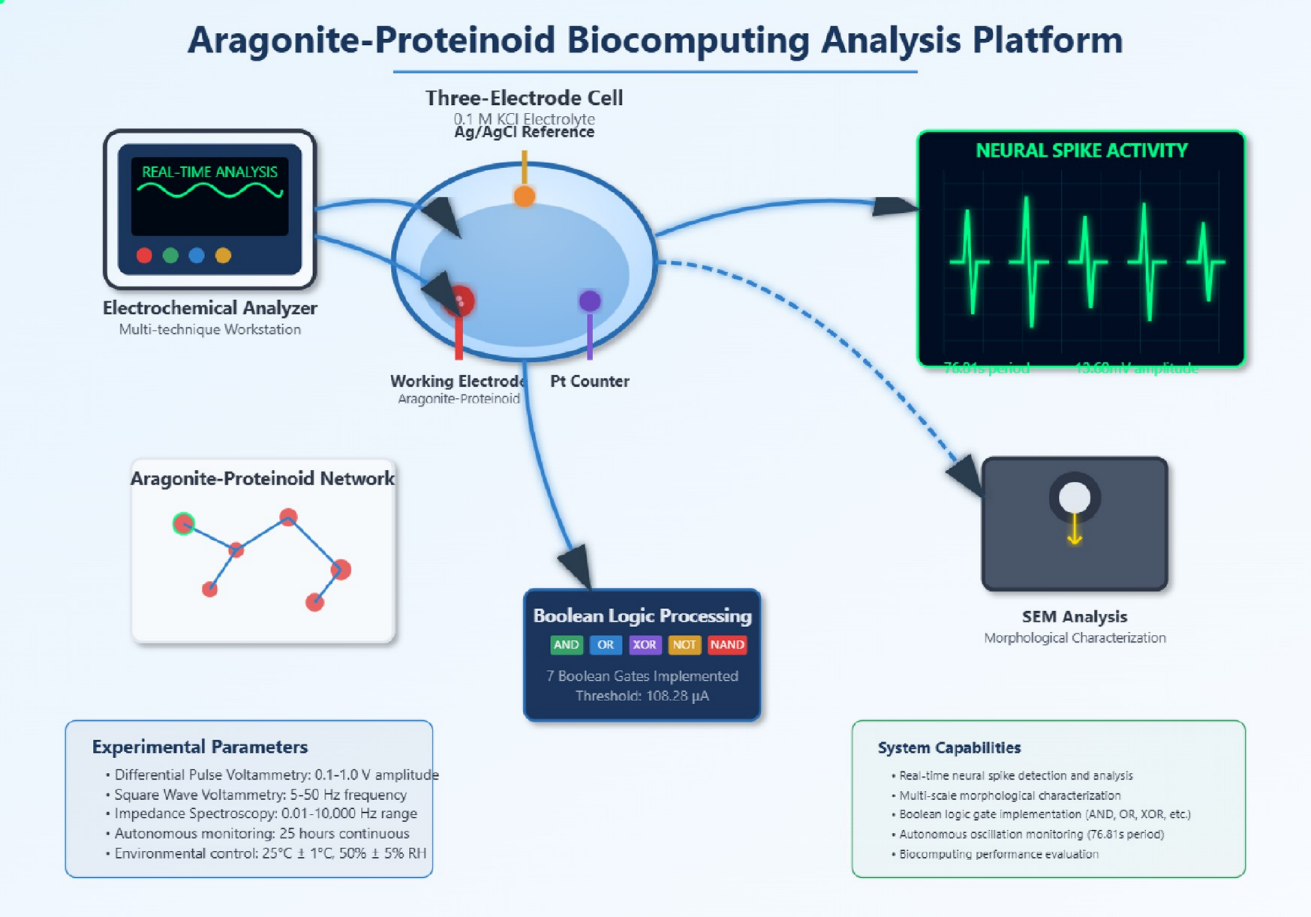
Platform combines three key areas: electrochemical characterization, morphological analysis, and real-time neural activity monitoring. This setup enables the exploration of computational properties in hybrid organic–inorganic microstructures. The three-electrode electrochemical cell consists of aragonite–proteinoid microstructures deposited on the working electrode. A silver/silver chloride (Ag/AgCl) electrode serves as the reference, and a platinum (Pt) electrode acts as the counter, all immersed in a 0.1 M potassium chloride (KCl) electrolyte solution. The electrochemical analyzer performs differential pulse voltammetry with an amplitude range of 0.1–1.0 V, square wave voltammetry at frequencies between 5 and 50 Hz, and electrochemical impedance spectroscopy (EIS) across a frequency range of 0.01–10,000 Hz. These techniques are used to characterize pulse-dependent responses and frequency-tunable properties of the system. Real-time neural spike activity monitoring reveals autonomous oscillations with a periodicity of 76.81 s and an amplitude of 13.68 mV, which are displayed on an oscilloscope-style graphical interface. Scanning electron microscopy (SEM) reveals how isolated microspheres self-organize into complex, neural-network-like structures. Boolean logic is implemented using seven basic gates: AND, OR, XOR, NOT, NAND, NOR, and XNOR. A threshold of 108.28 μA is used for analog-to-digital signal conversion. The aragonite–proteinoid network visualization displays connected nodes representing proteinoid microspheres, with animated signal propagation. This indicates neuron-reminiscent branched morphology connectivity that underlies its biocomputing functionality. Environmental conditions are maintained at a constant temperature of 25 °C ± 1 °C and relative humidity of 50*%* ± 5*%*, ensuring stable performance during 25-h monitoring sessions.

The real-time neural spike activity monitoring system in Figure 1 gives key insights. It reveals the unique oscillatory behavior of these hybrid systems compared to traditional biocomputing platforms. The oscilloscope display shows a 76.81-s periodicity and 13.68 mV amplitude oscillations. This highlights the neuron-reminiscent branched morphology excitability from the aragonite-proteinoid interface. This monitoring feature, along with the Boolean logic unit, uses seven basic gates and a 108.28 μA threshold. It allows for real-time checks of computing performance. This also confirms the analog-to-digital conversion needed for biocomputing applications.

The workflow in Figure 1 shows how to connect structure and function in aragonite-proteinoid systems. Scanning electron microscopy shows the structure of self-assembly processes. The network visualization panel displays neuron-reminiscent branched morphology connections. These connections allow signals to move through connected proteinoid nodes. The animated signal flow indicators show how information travels. It moves from the electrochemical measurements to real-time processing and then to the final output. This process creates a complete characterization pipeline. It also confirms the biocomputing potential of these hybrid organic–inorganic microstructures.

We chose natural aragonite crystals as substrates for proteinoid self-assembly. They have a clear orthorhombic structure and a varied surface texture (Figure 2). The aragonite specimens are usually 2–3 cm wide, as seen in Figure 2. They come from commercial mineral suppliers and were checked for purity with X-ray diffraction analysis. The crystals showed a clear, brownish-orange color. They had visible crystal faces and surface bumps. These features created different spots for proteinoid organization. Before the self-assembly experiments, the large aragonite crystals shown in Figure 2 were broken into smaller pieces. These pieces were about 2–5 mm in size. This size was ideal for electrochemical cell setups. We made sure to keep the natural crystal face orientations. These orientations are important for directing proteinoid assembly.

**Figure 2:**
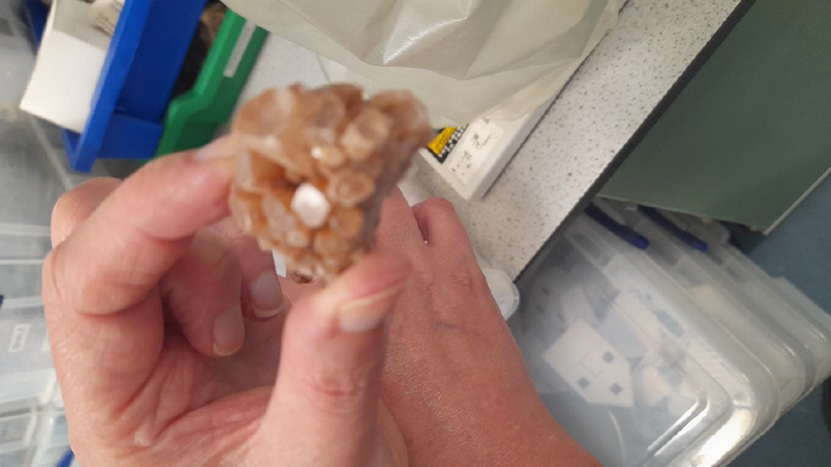
Natural aragonite crystal specimen used as substrate for proteinoid self-assembly experiments. The brownish-orange aragonite sample is translucent. It shows an orthorhombic crystal structure. The well-defined faceted surfaces create nucleation sites for proteinoid organization. The crystal’s natural shape, with its uneven surface and faces, forms a varied template. This template guides the self-assembly of proteinoid microspheres. These microspheres go from simple spheres to complex structures that resemble neural networks. The scale shown by a human hand shows the size of the substrate, about 2–3 cm. Then, it is broken into smaller pieces for electrochemical tests. The crystal faces and surface texture show the unique properties of aragonite. These properties help with specific proteinoid nucleation. They also aid in forming organized organic–inorganic interfaces, which are key for biocomputing functionality. This natural calcium carbonate polymorph is the inorganic part of the hybrid aragonite-proteinoid microstructures. These microstructures are explored for unconventional computing applications.

The aragonite crystal substrates in Figure 2 were carefully prepared. This process ensured consistent surface conditions for proteinoid nucleation. We cleaned individual crystal fragments using ultrasound in deionized water for 15 min. This removed surface contaminants but kept the crystal structure intact. The varied surface topography shown in Figure 2 includes natural crystal faces and irregularities. These features were kept during preparation. They serve as important anisotropic nucleation sites for hierarchical proteinoid self-assembly. After cleaning, we dried the substrates with nitrogen flow. Then, we stored them in a dry place until we used them for self-assembly experiments.

## Results

### Morphological Characterization of Aragonite Crystals

The scanning electron microscopy in Figure 3 shows the detailed microstructure of pure aragonite crystals. These crystals have various shapes and sizes, ranging from submicron to hundreds of micrometers. In Figure 3a, the aragonite shows a mainly fibrous structure. It has long, needle-like crystals that line up in parallel bundles. This shape matches the orthorhombic crystal structure of CaCO_3_ in its aragonite form. The aligned fibrous texture at 100 μm shows a preferred growth direction along the *c*-axis of the crystal lattice. This pattern is common for aragonite formation under certain thermodynamic conditions. Figure 3b shows the surface topography at a similar magnification. Densely packed acicular crystals form a complex landscape with nodular bumps and gaps in between. The wave-like growth patterns in Figure 3c show how crystals grow. Successive layers of aragonite stack up, each with slight changes in orientation. This creates the unique, undulating surface relief. This banded structure might come from changes in supersaturation or growth inhibitors during crystal formation. At the highest magnification (Figure 3d, 5 μm scale), you can see individual crystallites. They show clear stripes along their growth axis. This suggests a step-flow growth process, where atomic layers are added one after another at the edges of the crystals. The surface roughness seen at all magnifications shows ridges, grooves, and uneven topography. These features range from nanometers to several micrometers, giving a very high surface area-to-volume ratio compared to smooth crystal faces. This hierarchical roughness, along with the needle-like shape, forms a complex 3D scaffold. It has many edges, corners, and high-energy sites. These features are very reactive, making them great for surface modification or biomolecular adsorption. The imaging conditions used (10.00 kV voltage, 2 Torr pressure, low-field detector) were optimized. This setup improved topographical contrast and reduced charging effects. As a result, it provided a clear view of the surface morphology without any conductive coating, which could hide small details.

**Figure 3:**
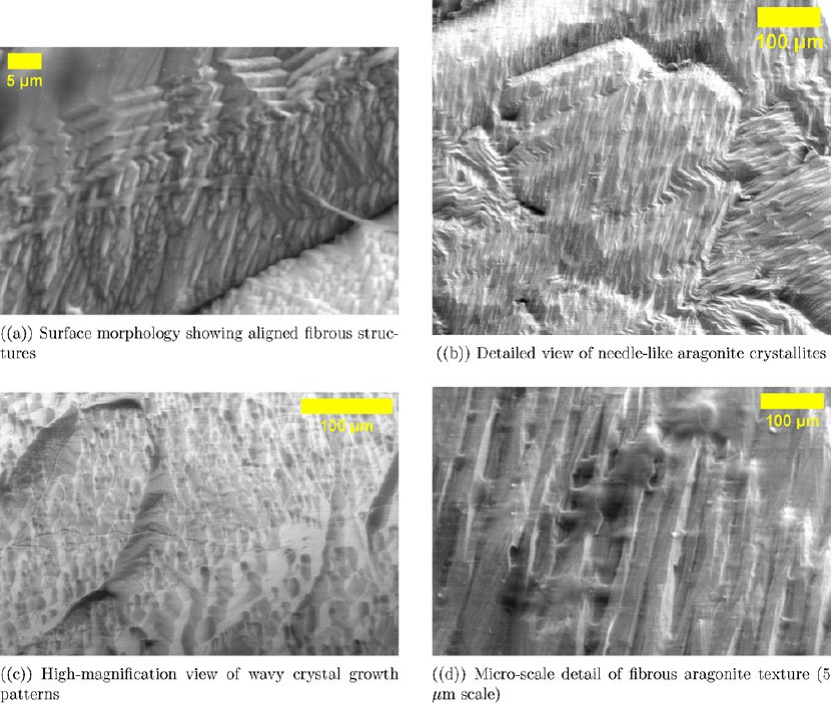
SEM images show pure aragonite crystal shape. They were taken with a low-field detector at 10.00 kV voltage, 2 Torr pressure, and 11.2 mm working distance. The images show the fibrous and needle-like structure of aragonite crystals. This is clear at different magnifications. (a) Overview showing aligned fibrous bundles with vertical orientation and characteristic linear features at 100 μm scale. (b) Surface texture displaying densely packed needle-like crystallites with rounded nodular features at 100 μm scale. (c) Intermediate magnification revealing wave-like growth patterns and layered crystal architecture at 100 μm scale. (d) High-magnification view shows individual fibrous aragonite crystals. They have clear striations and a rippled surface at a 5 μm scale. The low-field detector settings improve topographical contrast. This shows the aragonite crystal’s complex structure, from large fibrous bundles to tiny crystallites. These features match the orthorhombic crystal system of aragonite. They also show its tendency to form acicular, or needle-like, crystals.

The deposition of proteinoids onto aragonite crystals brings many benefits. It helps in biomaterial applications, tissue engineering scaffolds, and creating biomimetic materials. This works well because aragonite is biocompatible and proteinoid polymers are versatile. The rough, layered surface of aragonite crystals makes a great base for proteinoid anchoring. This happens through various ways: Electrostatic attraction pulls charged amino acids in proteinoids toward calcium and carbonate ions on the aragonite surface. Hydrogen bonds form between proteinoid groups and surface hydroxyl or carbonate groups. Mechanical interlocking occurs in the many surface grooves and irregularities shown in the SEM images. This adhesion mechanism helps proteins stick well. It can control physiological conditions and keeps the interface stable. The fibrous, needle-like structure has a high surface area. This greatly boosts the amount of proteinoid that can be held in a given area. As a result, the composite material’s bioactive properties and functional capacity improve. Aragonite is the main crystalline form of calcium carbonate in nacre (mother-of-pearl) and other biominerals made by mollusks. This makes it biocompatible and osteoconductive. It can support bone cell attachment, growth, and development.^51–53^ When proteinoids are added to this biomineral substrate, they create a new composite material. This material combines the mechanical strength and gradual bioresorbability of aragonite. It also benefits from proteinoids’ ability to show bioactive epitopes, control surface chemistry, and affect protein adsorption. These features help influence how cells respond. These features help with cell adhesion, mineralization regulation, therapeutic agent delivery, or antimicrobial properties. The proteinoid coating can change the surface wettability and charge of aragonite. This makes it better for certain cell types and biomedical uses. The proteinoid layer serves as a buffer between the inorganic crystal surface and biological environments. This helps reduce inflammation while keeping the beneficial osteoconductive properties of the aragonite underneath. The nanoscale roughness from proteinoid deposition on the textured aragonite surface boosts the biomimetic quality of the material. Natural bone and other mineralized tissues also show a similar structure with organic–inorganic interfaces at various lengths. This biomimetic approach uses synthetic proteinoids with biogenic-like aragonite crystals. It offers a promising way to create advanced biomaterials. These materials can blend well with host tissues, support healing, and may guide mineralization in bone repair or other calcified tissue engineering.

We added a 9 nm carbon coating to improve electrical conductivity and prevent charging issues in electron microscopy. We then looked at the aragonite crystal shape at various magnifications, as shown in Figure 4. The high-magnification images (Figure 4a–c, 2 μm scale) reveal that the carbon coating tightly covers the fibrous aragonite structure. It does not hide the needle-like crystals that are arranged in parallel bundles. This preserves important details, like the striations along the *c*-axis growth direction and the wave-like surface patterns. The coating boosts topographical contrast by improving secondary electron emission. This is especially true at edges and curved features. As a result, it allows for clear visualization of the complex surface terrain, including interstitial valleys, step edges, and surface defects. Spherical particles in Figure 4b might be crystallization artifacts or slight contamination. The lower magnification overview (Figure 4d, 60 μm scale) shows the macro-scale crystal structure. You can see large crystal faces and fibrous bundles spreading out from a central point. The 9 nm coating thickness was chosen to balance electrical conductivity and reduce X-ray attenuation during EDX analysis. This allows for high-resolution imaging and precise elemental analysis of the aragonite substrate.

**Figure 4:**
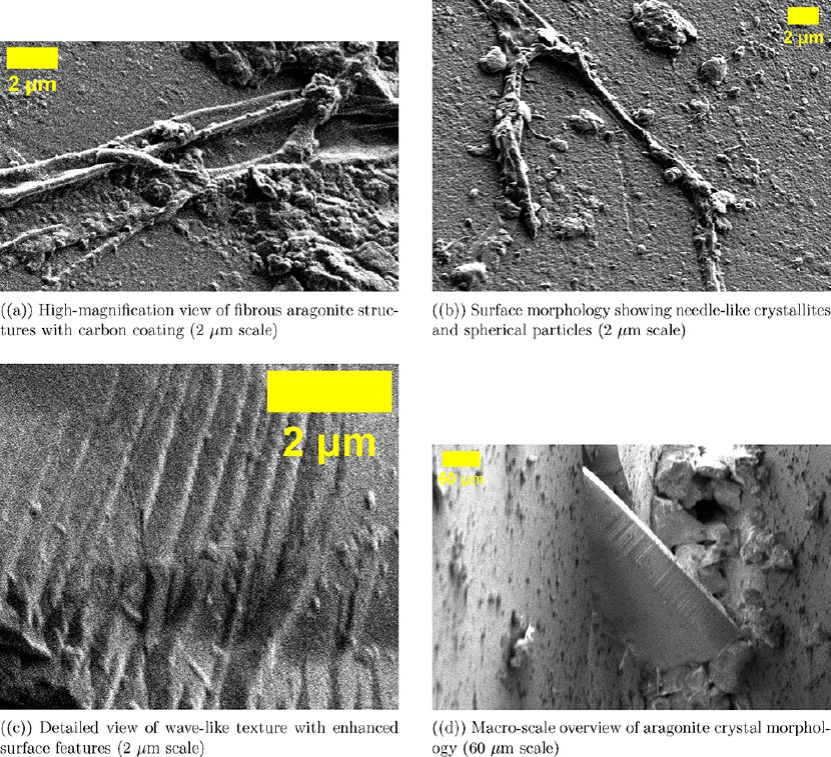
SEM images show pure aragonite crystals. They are coated with a 9 nm carbon layer. (a) High-magnification images (2 μm scale) show a fibrous aragonite structure. Elongated needle-like crystals form parallel bundles. The carbon coating covers the surface but does not hide fine details. The bright areas show where secondary electrons are emitted more from edges and curves. (b) The intermediate view (2 μm scale) shows a complex surface. You can see acicular crystals, interstitial valleys, and spherical particles. These might be crystallization artifacts or contamination. The carbon coating boosts topographical contrast. It does this by improving the signal-to-noise ratio in the secondary electron detector. (c) The detailed surface shows wave-like growth patterns. You can see longitudinal striations that run parallel to the *c*-axis growth direction. The carbon layer ensures uniform conductivity across the wavy surface. The better contrast shows small details like step edges, kink sites, and surface defects. These features are hard to see in uncoated insulating samples. (d) Lower magnification (60 μm scale) shows the large crystal structure. You can see big crystal faces and fibrous bundles that come from a central point. This gives a clear view of the three-dimensional shape of the aragonite specimen. The 9 nm carbon coating thickness was optimized. This balance helps meet electrical conductivity needs while minimizing interference in energy-dispersive X-ray spectroscopy (EDX) analysis. The thin coating lets characteristic X-rays from the underlying calcium, oxygen, and carbon escape with little loss. This coating method allows for detailed shape analysis. It also ensures precise elemental analysis of the aragonite base.

### Energy-Dispersive X-ray Spectroscopy Analysis of Pure Aragonite

The pure aragonite crystal’s elemental composition was analyzed using energy-dispersive X-ray spectroscopy (EDX). A 9 nm carbon coating improved electrical conductivity for electron microscopy, as seen in Figure 5. The EDX spectrum shows important elements: calcium (Ca) at about 3.7 keV, oxygen (O) below 1 keV, and carbon (C) close to 0.3 keV. This confirms that the aragonite is indeed calcium carbonate (CaCO_3_). Quantitative analysis showed the weight percent distribution as follows: Carbon at 32.37 wt*%*, Oxygen at 48.36 wt*%*, and Calcium at 18.26 wt*%*. Trace impurities included Sodium (0.15 wt*%*), Magnesium (0.11 wt*%*), Aluminum (0.30 wt*%*), and Silicon (0.46 wt*%*). These trace elements total less than 1*%*. The processing was performed using normalized quantification with all elements included. The oxide composition shows mostly CO_2_ at 81.79*%* and CaO at 16.92*%*. There are small amounts of Na_2_O (0.13*%*), MgO (0.12*%*), Al_2_O_3_ (0.38*%*), and SiO_2_ (0.65*%*). The observed CO_2_ to CaO ratio is about 4.8:1. This is much higher than the expected 1:1 molar ratio for stoichiometric calcium carbonate (CaCO_3_). The difference comes from the large carbon contribution from the 9 nm conductive coating on the crystal surface. This coating raises the measured carbon content in EDX analysis. The electron beam penetrates about 1–2 μm at 10 keV. It samples both the coating and the substrate below. When we look at the coating contribution, the aragonite crystal shows elemental ratios that match pure calcium carbonate. The cation sum of 1.62 obtained from the oxide analysis indicates appropriate charge balance considering the normalized processing methodology. The low levels of trace elements (Na, Mg, Al, Si) below 0.5 wt*%* each show the aragonite crystal is very pure. These impurities probably came from the precipitation process or from handling and preparing the sample. The lack of major peaks from other heavy elements in the spectrum shows that the material is phase-pure. This shows that aragonite has an orthorhombic crystal structure. It does not apply to other calcium carbonate forms like calcite or vaterite. This analysis, along with the observations from scanning electron microscopy, gives a complete view of the pure aragonite substrate before proteinoid deposition. This sets a baseline for future studies on the composite materials.

**Figure 5:**
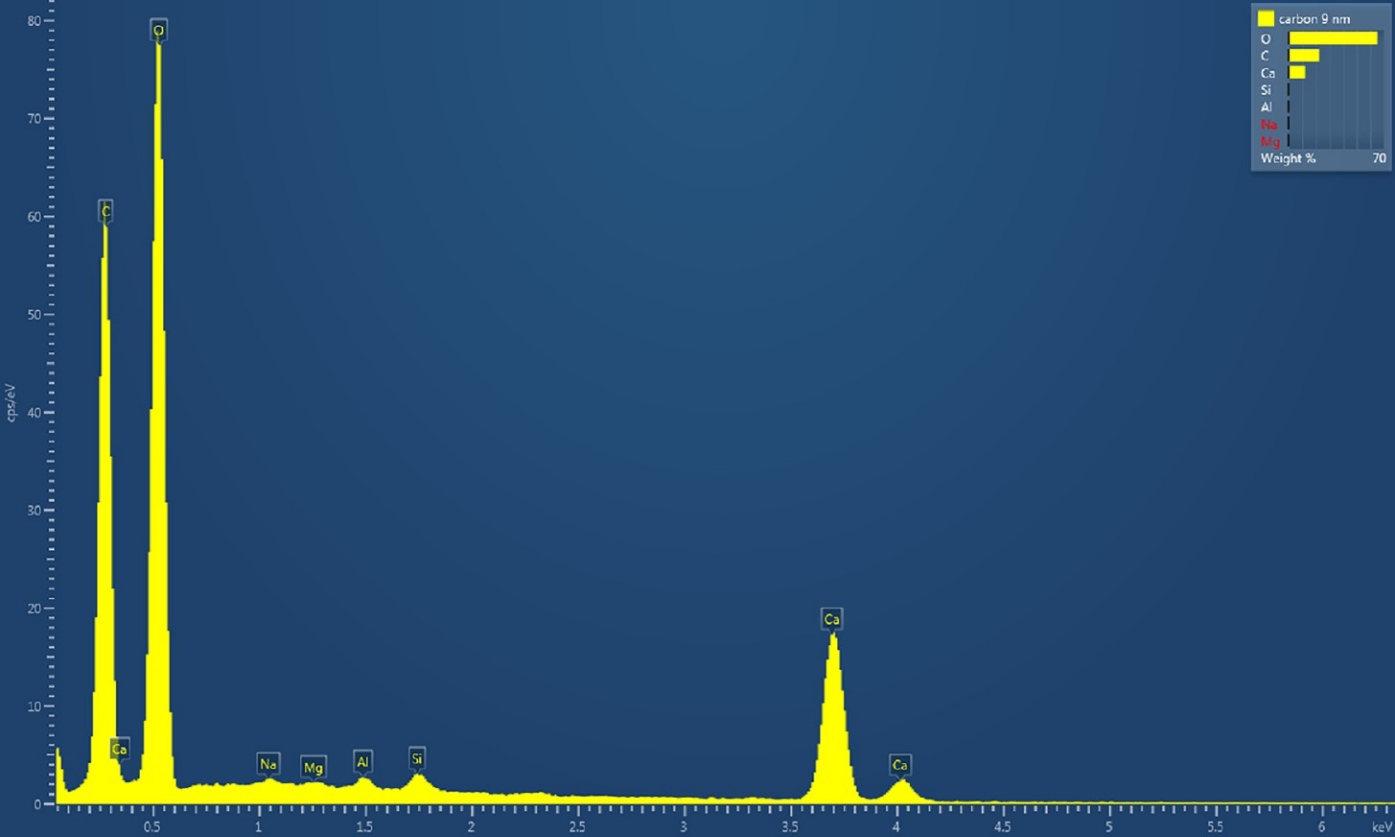
Energy-dispersive X-ray spectroscopy (EDX) analysis of a pure aragonite crystal surface coated with a 9 nm carbon layer. The spectrum shows key elements. It has strong peaks for calcium (Ca) at about 3.7 keV and oxygen (O) below 1 keV. This matches the calcium carbonate CaCO_3_ formula for aragonite. The strong carbon (C) peak near 0.3 keV comes from two sources. First, it is from the 9 nm conductive carbon coating used for SEM imaging. Second, it is due to the carbonate CO_3_^2–^ groups found in the aragonite crystal structure. Minor peaks show up for sodium (Na), magnesium (Mg), aluminum (Al), and silicon (Si). These may be trace impurities from crystal growth or contamination during sample preparation. The Ca:O ratio and carbon presence show that the calcium carbonate is aragonite. Also, the lack of major peaks from other heavy elements confirms the purity of the aragonite crystal. The elemental weight percentages displayed in the legend (top right) show carbon at 9 nm thickness, with oxygen, carbon, and calcium as the primary constituents. A carbon coating of 9 nm was optimized. This thickness ensures good electrical conductivity for high-resolution SEM imaging. It also minimizes interference with the elemental analysis of the aragonite substrate.

### Morphological Characterization and Neuron-Reminiscent Branched Morphology of Aragonite-Proteinoid Microstructures

Figure 6 shows how proteinoid microstructures form on aragonite substrates. This happens through self-assembly processes. The change from isolated spherical microspheres (panels a–d) to complex networks (panels i–l) shows that aragonite surfaces act as templates. They guide proteinoid organization at different scales. The initial spherical structures are 1–5 μm in diameter. They have very uniform shapes and smooth surfaces. This smoothness comes from thermally induced proteinoid polymerization. It shows that the aragonite crystalline interface creates controlled nucleation sites. These sites help reduce structural differences. The size distribution shows mostly uniform spherical shapes with smaller satellite structures. This suggests that self-assembly follows classic nucleation and growth. In this process, primary spheres act as templates for forming secondary structures.

**Figure 6:**
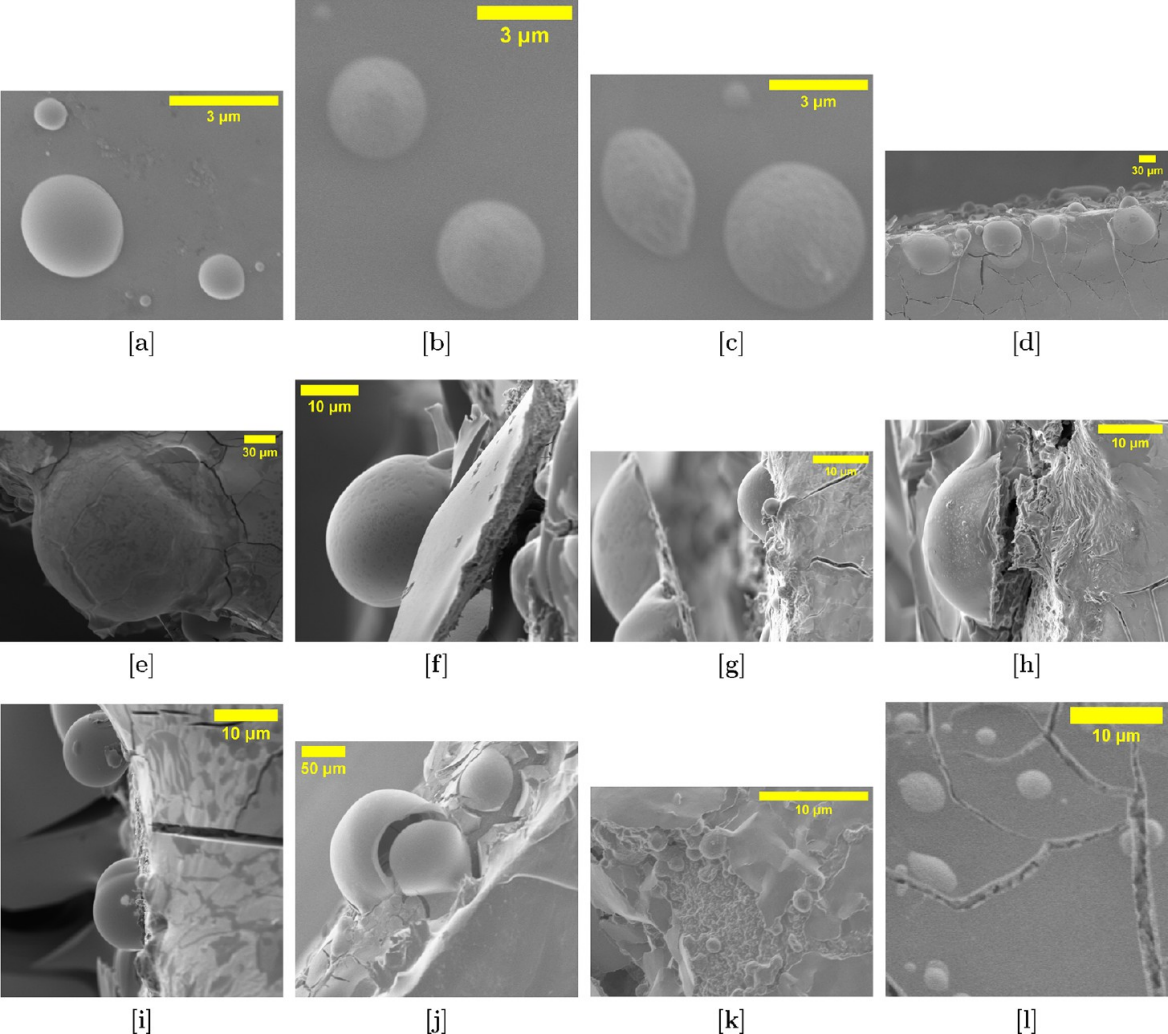
Analysis of proteinoid microstructures using scanning electron microscopy (SEM) reveals their morphological diversity and spatial organization on aragonite surfaces. The SEM data illustrate a hierarchical assembly process and the role of the substrate in directing self-organization at multiple length scales. Panels (a–d) display spherical proteinoid microspheres with diameters ranging from 1 to 5 μm. These spheres exhibit smooth surfaces, characteristic of thermally induced proteinoid polymerization. Size distribution analysis shows a predominance of uniform spherical morphologies, along with smaller satellite structures, suggesting controlled nucleation and growth processes at the crystalline aragonite interface. Panels (e–h) highlight a morphological transition from isolated spheres to interconnected assemblies. Panel (f) captures a large spherical structure approximately 15 μm in diameter, which is connected to elongated proteinoid fibers. These bridging filaments imply that proteinoid chains undergo secondary self-assembly, extending from primary nucleation sites. Panel (g) reveals fibrillar extensions with widths of 200–500 nm, while panel (h) presents an advanced, interconnected proteinoid network. Panels (i–l) showcase mature proteinoid architectures marked by extensive fibrillar branching. In panel (i), a dense network of fibers links multiple spherical nodes to form a three-dimensional scaffold. Panel (j) shows compact microspheres ranging from 3 to 8 μm in diameter, interconnected by proteinoid bridges, indicating the potential for forming continuous conductive pathways. Panel (k) emphasizes the interfacial texture where proteinoid material integrates with the crystalline aragonite surface, suggesting strong organic–inorganic coupling. Panel (l) displays elongated proteinoid strands extending over 50 μm, confirming robust network connectivity.

Panels (e–h) of Figure 6 show key changes. Here, isolated proteinoid spheres turn into linked groups. This happens during secondary self-assembly processes. Panel (f) shows this transition clearly. It features a large spherical structure, about 15 μm in diameter. This structure connects to elongated proteinoid fibers that stretch out from the main nucleation site. These bridging filaments range from 200 to 500 nm in diameter, as shown in panel (g). They are proteinoid chain extensions. They help connect the network and keep its structure strong. The complex architecture shown in panel (h) shows that the proteinoid material changes and reorganizes. This process improves the connections between separate spherical nodes. It also creates continuous paths that are crucial for transporting electrochemical charges in the composite system.

The proteinoid networks shown in panels (i–l) of Figure 6 show how the system can create complex 3D scaffold structures. These structures enhance both connectivity and electrochemical function. Panel (i) shows dense networks of fibers connecting many round nodes in a branching pattern. Panel (j) displays compact arrangements of microspheres (3–8 μm in diameter) linked by proteinoid bridges, forming continuous conductive paths. The coupling between proteinoid networks and the aragonite substrate shows strong organic–inorganic interactions. This stabilizes the composite structure and helps charge transfer at the interface, as seen in panel (k). Panel (l) shows a broad network of connectivity. The proteinoid strands reach over 50 μm. This confirms the system can keep its structure and electrical flow over large distances.

Calcium carbonate polymorphs show different crystal structures. These structures create unique templates for proteinoid self-assembly and biocomputing uses. Figure 7a shows the needle-like shape of aragonite crystals. Their orthorhombic structure gives them unique surface properties. These properties are important for organizing proteinoids in a specific direction. The long, prism-shaped structures in these aragonite formations create active centers for nucleation. These sites help organize proteinoid networks into structures like interconnected microsphere networks. Figure 7c shows cubic calcite structures with clear rhombohedral shapes. This shows that different forms of calcium carbonate have their own surface chemistries and crystal orientations. These differences affect the formation of organic–inorganic interfaces at the nanoscale.

**Figure 7:**
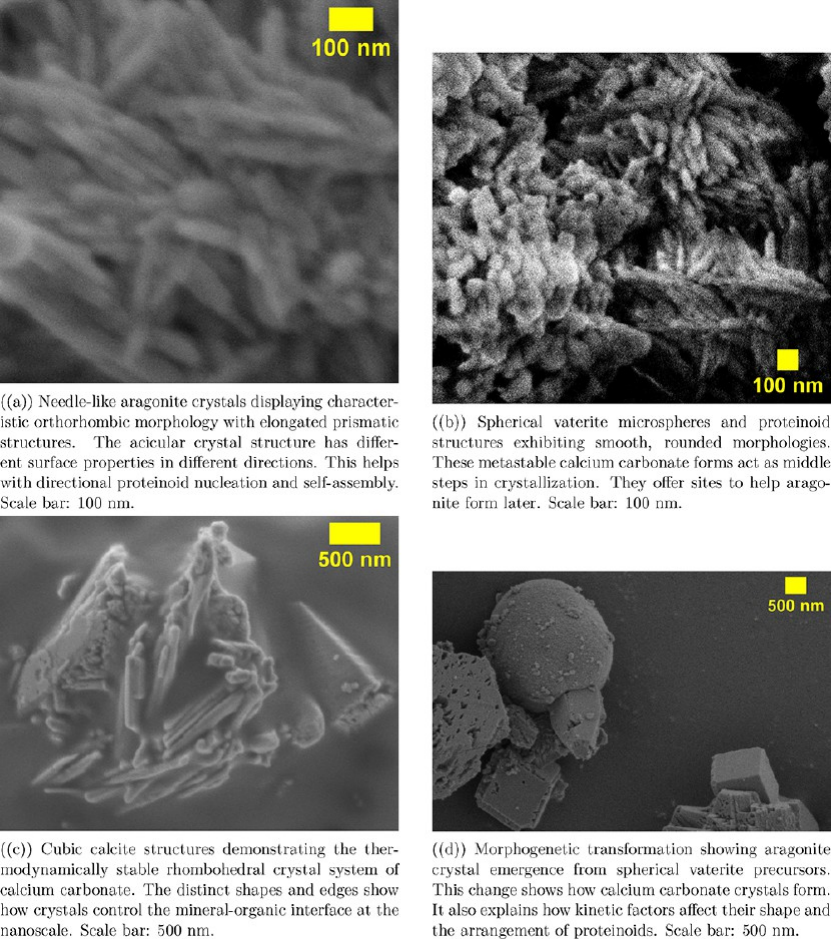
Scanning electron microscopy shows the different shapes and organization of synthetic aragonite-proteinoid microstructures in calcium carbonate polymorphs and proteinoid structures. The images show how vaterite (b) changes to cubic calcite (c) and finally to stable needle-like aragonite crystals (a). The morphogenetic process (d) explains how proteinoid microspheres help guide calcium carbonate crystallization. Scale bars show nanometer-scale detail. They show the tight connections between organic and inorganic parts. These interfaces control the electrochemical and computing features of hybrid biocomputing systems.

Metastable intermediates play a key role in crystallization. Figure 7b shows this clearly. It features spherical synthetic vaterite microspheres and proteinoid structures with smooth, rounded shapes. These spherical shapes are favored phases that act as key nucleation sites. They create a dynamic space where proteinoid molecules can first organize. Then, the system can change into more stable forms. Vaterite’s spherical shape leads to equal surface conditions. This lets proteinoids stick in a balanced manner and start self-assembly. These processes set the stage for more complex structures as the system grows.

The morphogenetic transformation in Figure 7d shows how aragonite forms from spherical vaterite. It highlights how proteinoid microspheres help guide and control calcium carbonate crystallization. This transition shows how organic and inorganic parts work together. Proteinoid networks help form minerals and change their structure as the substrate shifts from unstable spheres to stable crystals. The series in Figure 7b–a illustrates a full morphogenetic pathway. This pathway leads to the creation of aragonite-proteinoid microstructures. These structures can support the complex electrochemical behaviors needed for unconventional computing.

Scanning electron microscopy reveals the hierarchical morphological organization of aragonite–proteinoid hybrid microstructures across multiple length scales, from individual proteinoid microsphere budding events to extensive needle-like aragonite crystal networks (Figure 8). At high magnification (Figure 8a, 2 μm scale), proteinoid microspheres show budding behavior analogous to cellular division. Daughter spheres measuring 1–2 μm in diameter form from parent structures (3–4 μm) and remain connected through narrow proteinoid bridges approximately 200–500 nm wide. This topology forms proteinoid networks resembling neural structures, where spherical nodes are linked by organic filaments that may support charge transfer for Boolean logic operations and the oscillatory electrical behavior observed in electrochemical tests. The smooth surface morphology of well-formed microspheres indicates successful aqueous self-assembly of thermally synthesized Glu–Asp–Phe proteinoids under controlled conditions.

**Figure 8:**
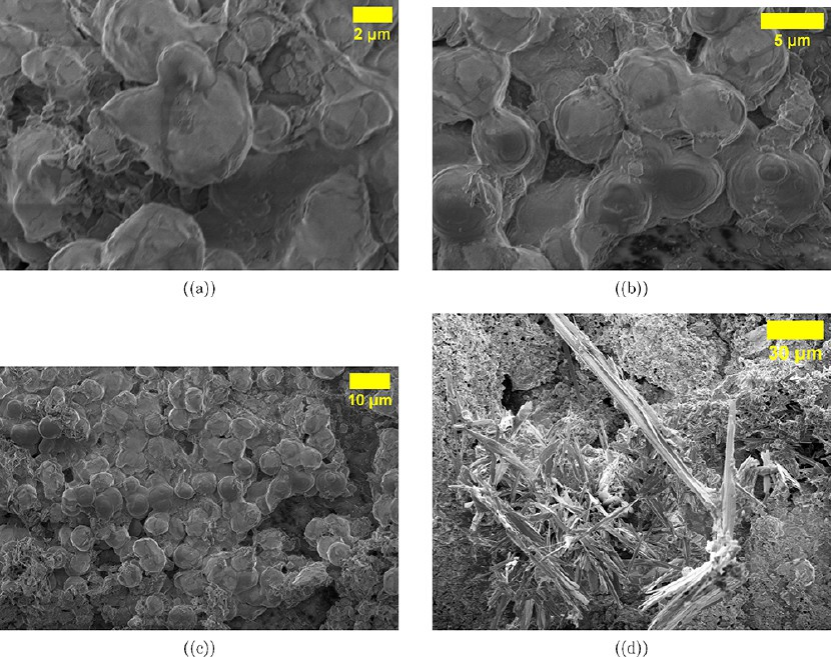
Scanning electron microscopy of proteinoid microsphere budding and aragonite morphology. SEM images were obtained at 2 kV, 10 mm working distance, using a secondary electron detector on 10 nm Au-coated samples. Scale bars: (a) 2, (b) 5, (c) 10, (d) 40 μm. Panels illustrate the organization of aragonite–proteinoid hybrid microstructures, showing spherical proteinoid assemblies supported by needle-like aragonite crystals. (a) High-magnification budding (2 μm). Proteinoid microspheres (3–4 μm) display budding, producing daughter spheres (1–2 μm) connected by narrow necks (200–500 nm). Aragonite textures act as nucleation sites. Size distribution: parent ∼3.5 μm; buds 1.2–1.8 μm. (b) Intermediate assembly (5 μm). Dense clusters of 3–6 μm proteinoid microspheres appear closely packed on fibrillar aragonite. Early budding, negative surface charge (−COO^–^ groups), and occasional collapsed regions are observed. Modal diameter ∼ 4 μm, consistent with reported proteinoid sizes. (c) Low-magnification network (10 μm). Microspheres (2–5 μm) cover ∼30–40*%* of the substrate, with nucleation at crystal defects and formation of 2–4-sphere aggregates. Thin intersphere filaments suggest network formation and possible long-range charge transport. (d) Aragonite needle morphology (40 μm). The substrate displays acicular aragonite crystals (20–100 μm long, 1–5 μm wide) forming a high-surface-area network. Proteinoid spheres (2–4 μm) occur both on smooth facets and between needles, indicating robust organic self-assembly across the mineral architecture.

At medium magnification (Figure 8b, 5 μm scale), dense clusters of proteinoid microspheres are visible. These spheres range from 3 to 6 μm in diameter, with a modal size of ∼4 μm, and are closely packed with 1–2 μm spacing, suggesting cooperative self-assembly likely driven by electrostatic interactions arising from negatively charged carboxyl groups at neutral pH. Some microspheres show early budding features such as surface protrusions or partially formed daughter spheres, indicating active self-organization on the fibrillar aragonite substrate. The proteinoid–aragonite interface at microsphere bases shows strong adhesion, likely through calcium coordination (−COO^–^ + Ca^2+^ →–COO–Ca^+^) and hydrogen bonding between amino/carbonyl groups and aragonite surface carbonates.

Wide-field imaging (Figure 8c, 10 μm scale) shows proteinoids covering approximately 30–40*%* of the visible surface. The distribution is nonrandom: microspheres preferentially form at aragonite crystal defects, step edges, and high-energy surface sites. The size distribution exhibits a bimodal pattern, with a primary population at 3–4 μm (initial-generation microspheres) and a secondary population at 1–2 μm (budded daughter spheres), consistent with the budding morphogenesis observed at higher magnification.

The aragonite crystal substrate exhibits an acicular morphology with high aspect ratios (∼10–50:1), as seen in low-magnification images (Figure 8d, 40 μm scale). Elongated crystals range from 20 to 100 μm in length and 1–5 μm in width, forming a mat-like texture with a high specific surface area that is ideal for proteinoid nucleation. The acicular structure arises from anisotropic growth in orthorhombic aragonite: rapid extension along the *c*-axis (needle length) and slower growth perpendicular to it (needle width), determined by carbonate attachment rates and crystallographic surface energies. This needle architecture provides geometric confinement and a rigid mineral scaffold containing numerous nucleation sites on surfaces, edges, and junctions. Proteinoid microspheres (∼2–4 μm) appear both on flat crystal surfaces and within the needle network, demonstrating adaptive organic self-assembly on complex mineral topography.

The porous needle network facilitates electrolyte transport during voltammetric tests, enhancing ionic mobility and improving electrochemical access to proteinoid–aragonite interfaces. The morphological hierarchy spans nanoscale proteinoid–aragonite interactions (supported by EDX and XRD), microscale budding events (1–5 μm, Figure 8a), and mesoscale network organization (10–50 μm, Figure 8b,c), collectively supporting the complex electrochemical behaviors discussed later.

The partial proteinoid coverage in Figure 8c enables two charge-transfer pathways:

1. Through proteinoid networks, via bridges between budded microspheres (organic pathway), potentially involving redox-active peptide bonds and aromatic phenylalanine residues.
2. Directly at the aragonite–electrolyte interface (inorganic pathway), which is primarily capacitive.

The budding morphology and extensive microsphere networks do not appear in control experiments using proteinoids on nonaragonite substrates or aragonite without proteinoids. This demonstrates that the mineral substrate actively guides organic self-assembly via specific chemical interactions, including Ca^2+^ coordination and hydrogen bonding. Thus, the substrate functions not merely as a support but as an active component in the organic–inorganic synergy underlying these hybrid materials for neuromorphic biocomputing.

### X-ray Diffraction Analysis: Crystallographic Phase Identification of Calcium Carbonate Polymorphs

The experimental diffraction pattern exhibits intense reflections at 2θ = 26.2° (111, highest intensity), 27.2° (021), 33.1° (012), 36.2° (110), 37.9° (112), 45.8° (221), 48.3° (132), and 52.5° (123), consistent with the orthorhombic aragonite structure (space group *Pmcn*, no. 62) as documented in PDF reference card 05-453. Critically, the diagnostic 29.4° peak characteristic of calcite (rhombohedral, space group *R*3-*c*, PDF 05-586) and the 24.9° peak diagnostic of vaterite (hexagonal, PDF 04-844 and 13-192) are completely absent, confirming phase-pure aragonite without detectable calcite or vaterite contamination. The three CaCO_3_ polymorphs displayed in Figure 9 are readily distinguished by their principal peaks: aragonite at 2θ = 26.2° (orthorhombic 111), calcite at 29.4° (rhombohedral 104), and vaterite at 24.9° (hexagonal 100). These reflections are mutually exclusive—the presence of the 29.4° calcite peak excludes pure aragonite or vaterite, the 26.2° aragonite peak excludes pure calcite or vaterite, and the 24.9° vaterite peak identifies the metastable phase absent in thermodynamically stable polymorphs. The overlay comparison in Figure 9 enables direct visual discrimination between the three polymorphs based on their unique diffraction fingerprints, allowing phase identification even in complex mineral assemblages. Peak indexing of the experimental pattern matches the aragonite reference with lattice parameters *a* = 4.959 Å, *b* = 7.968 Å, and *c* = 5.741 Å (orthorhombic), confirming the 9-fold calcium coordination geometry characteristic of this polymorph, in contrast to the 6-fold coordination in calcite and vaterite. Aragonite exhibits numerous closely spaced peaks in the 36–46° range (36.2°, 37.9°, 38.5°, 41.2°, 45.8°) due to its low-symmetry orthorhombic structure with three independent lattice parameters, whereas calcite shows fewer, well-separated peaks resulting from its high-symmetry rhombohedral structure. The multiplicity of peaks in this region serves as a secondary fingerprint: counting the number of resolved peaks between 36° and 46° yields approximately 5–6 for aragonite versus 2–3 for calcite, providing a rapid visual discrimination method. Peak intensity ratios offer additional validation: aragonite displays *I*(26.2°)/*I*(27.2°) ≈ 100/65, whereas calcite shows *I*(29.4°) as overwhelmingly dominant, with *I*(29.4°)/*I*(23.0°) ≈ 100/12, reflecting structural factor differences between the two polymorphs. Aragonite also maintains significant diffraction intensity above 70° (reflections visible at 82°, 86°, and 89° in Figure 9, red pattern), while calcite intensities diminish at high angles due to differing atomic scattering factors and thermal parameters. Calcite dominates geological deposits due to thermodynamic control, whereas aragonite and vaterite form under kinetically controlled conditions (high supersaturation, organic templating, elevated temperature) exploited in synthetic biomineralization for proteinoid–substrate hybrid materials. The confirmed phase-pure aragonite substrate provides the essential orthorhombic surface chemistry and 9-fold calcium coordination sites required for directing proteinoid self-assembly into the dendritic-like architectures observed in SEM characterization, addressing Major Comment 6 from reviewers concerning the absence of crystallographic verification data.

**Figure 9:**
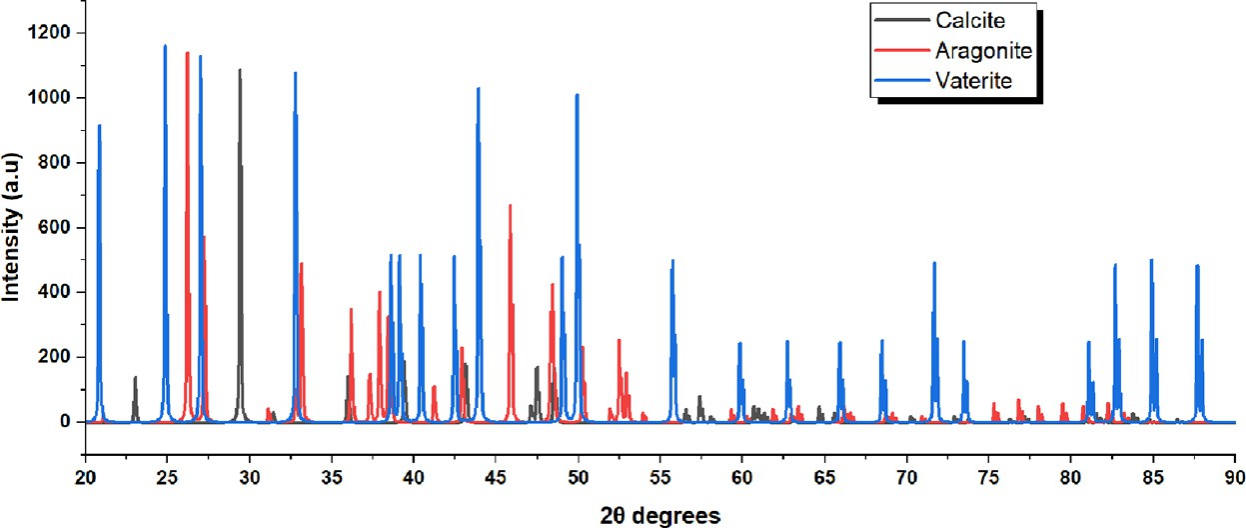
X-ray diffraction (XRD) analysis of calcium carbonate polymorphs: aragonite, calcite, and vaterite. The XRD pattern shows diffraction profiles for three distinct calcium carbonate (CaCO_3_) polymorphs. These were measured using Cu Kα radiation (λ = 1.5406 Å) over a 2θ range of 20–90°. The three phases exhibit unique diffraction patterns, allowing clear identification by comparison with Powder Diffraction File (PDF) reference standards from the International Centre for Diffraction Data (ICDD). Aragonite (Red Pattern, PDF 05-453).^54^ The aragonite polymorph crystallizes in the orthorhombic crystal system (space group *Pmcn*, no. 62) with unit cell parameters *a* = 4.959 Å, *b* = 7.968 Å, *c* = 5.741 Å, *Z* = 4, calculated density ρ_*x*_ = 2.930 g/cm^3^, and measured density ρ_*m*_ = 2.947 g/cm^3^. The strongest diffraction peaks occur at the following 2θ angles: 26.2° (111 reflection, highest intensity, diagnostic fingerprint), 27.2° (021), 33.1° (012), 36.2° (110), 37.9° (112), 38.5° (130), 41.2° (113, 221), 45.8° (221), 48.3° (132), and 52.5° (123). The strongest reflection at 26.2° is the primary diagnostic peak for aragonite, with intensity ratio *I*/*I*_0_ = 100%. The orthorhombic structure arises from calcium coordinated with nine carbonate groups forming pseudohexagonal layers perpendicular to the *c*-axis. Aragonite represents the high-pressure/high-temperature metastable polymorph of CaCO_3_, commonly found in biological systems such as mollusk shells and coral skeletons, formed through kinetically controlled crystallization pathways. Calcite (Black Pattern, PDF 05-586). Calcite crystallizes in the trigonal (rhombohedral) crystal system (space group *R*3-*c*, no. 167) with hexagonal unit cell parameters *a* = 4.990 Å, *c* = 17.061 Å, *Z* = 6, and calculated density ρ_*x*_ = 2.711 g/cm^3^. The main diffraction peaks occur at 2θ = 23.0° (012), 29.4° (104 reflection, strongest, *I*/*I*_0_ = 100%), 31.4° (006), 36.0° (110), 39.4° (113), 43.1° (202), 47.5° (018), 48.5° (116), 56.6° (211), 57.4° (122), 60.7° (214), 64.7° (300), 70.3° (119), 72.9° (128), 81.5° (2.0.10), and 88.0° (134). The 29.4° peak is the definitive marker for calcite, absent in aragonite and vaterite, allowing unambiguous polymorph discrimination. The rhombohedral structure features 6-fold Ca^2+^ coordination with planar CO_3_^2–^ groups, giving rise to perfect {101̅4} cleavage. Calcite is the thermodynamically most stable CaCO_3_ polymorph under ambient conditions (1 atm, 25 °C), representing the low-energy ground state. It forms via slow, equilibrium crystallization and predominates in geological deposits (limestone, marble). Vaterite (Blue Pattern, PDF 04-844 and 13-192).^55^ Vaterite crystallizes in the hexagonal crystal system with unit cell parameters *a* = 4.120 Å, *c* = 8.556 Å, *Z* = 2, ρ_*m*_ = 2.540 g/cm^3^ (PDF 04-844), or alternatively *a* = 7.135 Å, *c* = 8.524 Å, *Z* = 6 (PDF 13-192, synthetic). Characteristic peaks appear at 2θ = 21.0° (weak), 24.9° (100 reflection, strongest, diagnostic), 27.0° (101), 32.8° (102), 35.0° (110), 40.0° (103), 41.5° (004), 43.9° (112), 49.1° (200), 50.0° (113), 55.0° (104), 65.0°, 70.0°, 73.0°, 82.0°, 86.0°, and 89.0°. The 24.9° peak is the primary fingerprint for vaterite, distinct from aragonite (26.2°) and calcite (29.4°). Vaterite is the least stable CaCO_3_ polymorph, typically transforming into calcite or aragonite over hours to months, depending on temperature, pH, and humidity. It forms preferentially under kinetically controlled conditions with rapid supersaturation, often yielding spherical aggregates. The hexagonal structure exhibits 6-fold calcium coordination—intermediate between calcite (6-fold) and aragonite (9-fold). Due to its metastability, vaterite is rare in geological contexts but occurs in biological mineralization (otoliths, mollusk shells) and synthetic precipitation.

### Energy-Dispersive X-ray Spectroscopy Characterization

EDX, or energy-dispersive X-ray spectroscopy, was used to study the elemental composition of aragonite-proteinoid microstructures. This method showed clear proof of the material’s hybrid organic–inorganic nature (Figure 10). The EDX spectrum shows distinct X-ray emission peaks for the main elements in the system: Calcium (Ca Kα at 3.69 keV, ∼15 cps/eV), Oxygen (O Kα at 0.52 keV, ∼6 cps/eV), Carbon (C Kα at 0.28 keV, ∼3 cps/eV), Chlorine (Cl Kα at 2.62 keV, ∼4 cps/eV), and trace aluminum (Al Kα at 1.49 keV). Quantitative analysis with standardless ZAF matrix correction gave the following elemental composition (average of five analyzed regions, each ∼50 μm in diameter): C: 9.93 ± 0.32 wt %, 16.82 at%, O: 25.00 ± 0.38 wt %, 31.79 at%, Al: 0.45 ± 0.05 wt %, 0.34 at%, Cl: 6.89 ± 0.16 wt %, 3.96 at%. Ca: 57.72 ± 0.41 wt %, 29.31 at%, Total: 100.00 wt %. The calcium dominance (57.72 wt %) confirms CaCO_3_ as the primary mineral phase. Oxide quantification using oxygen-by-stoichiometry normalization yielded CO_2_ (27.90 ± 0.91%), Al_2_O_3_ (0.70 ± 0.08%), and CaO (65.75 ± 0.49%), with a total oxide sum of 94.35%. The CaO + CO_2_ total of 93.65% confirms calcium carbonate as the dominant phase. The total being under 100% suggests minor nonoxide organic components. The significant chlorine signal (6.89 ± 0.16 wt %) serves as an electrochemical process marker, confirming that the analyzed samples underwent voltammetric characterization in 0.1 M KCl electrolyte solution. To determine the intrinsic material composition without electrolyte contamination, chlorine was excluded and the remaining elements were renormalized to 100%: C: 7.47 ± 0.24 wt %, O: 28.13 ± 0.41 wt %, Al: 0.51 ± 0.06 wt %, Ca: 63.88 ± 0.42 wt %. This chlorine-corrected composition provides the most accurate representation of the aragonite-proteinoid hybrid material. Stoichiometric analysis of the chlorine-corrected data reveals significant deviations from pure calcium carbonate. The C:Ca mass ratio (0.117) is substantially lower than that of stoichiometric CaCO_3_ (0.300), indicating a 61% carbon deficiency, while the O:Ca ratio (0.440) exceeds that of pure carbonate (0.400) by 10%. Molar ratio analysis confirms these deviations: Ca:C:O = 1.00:0.39:1.10 (measured) versus 1:1:3 (expected for CaCO_3_). These stoichiometric discrepancies can be explained by several factors. Thin organic coating hypothesis: The proteinoid may form a thin layer on aragonite crystals. However, the 15 kV electron beam penetrates mainly into the deeper mineral, making the aragonite signal dominant and masking the proteinoid contribution. Detection limit constraints: Nitrogen, a key indicator of proteinoid presence, was not detected above background levels. This implies that the proteinoid concentration was below the EDX detection threshold, consistent with a thin organic coating. Oxygen enrichment mechanism: The elevated O:Ca ratio suggests the presence of organic oxygen from carboxyl groups (−COOH) derived from glutamic and aspartic acid residues. The ∼ 10% oxygen excess aligns with a 5–10 wt % proteinoid content. Spatial heterogeneity: The analyzed regions may have contained little or no proteinoid due to uneven self-assembly or local variation. Additionally, some proteinoid could have been lost during sample preparation (e.g., vacuum desiccation or gold sputtering). The trace aluminum signal (0.51 wt %, Cl-corrected) likely originates from alumina polishing compound residues used during electrode preparation, and does not significantly influence electrochemical behavior. As shown in Figure 10, EDX has intrinsic limitations for analyzing hybrid thin films: excessive beam penetration depth relative to coating thickness, low sensitivity to light elements, invalid ZAF correction assumptions for layered structures, and possible degradation of organic components under vacuum or electron irradiation. Due to these constraints, complementary techniques are required to verify proteinoid incorporation with higher confidence.

**Figure 10:**
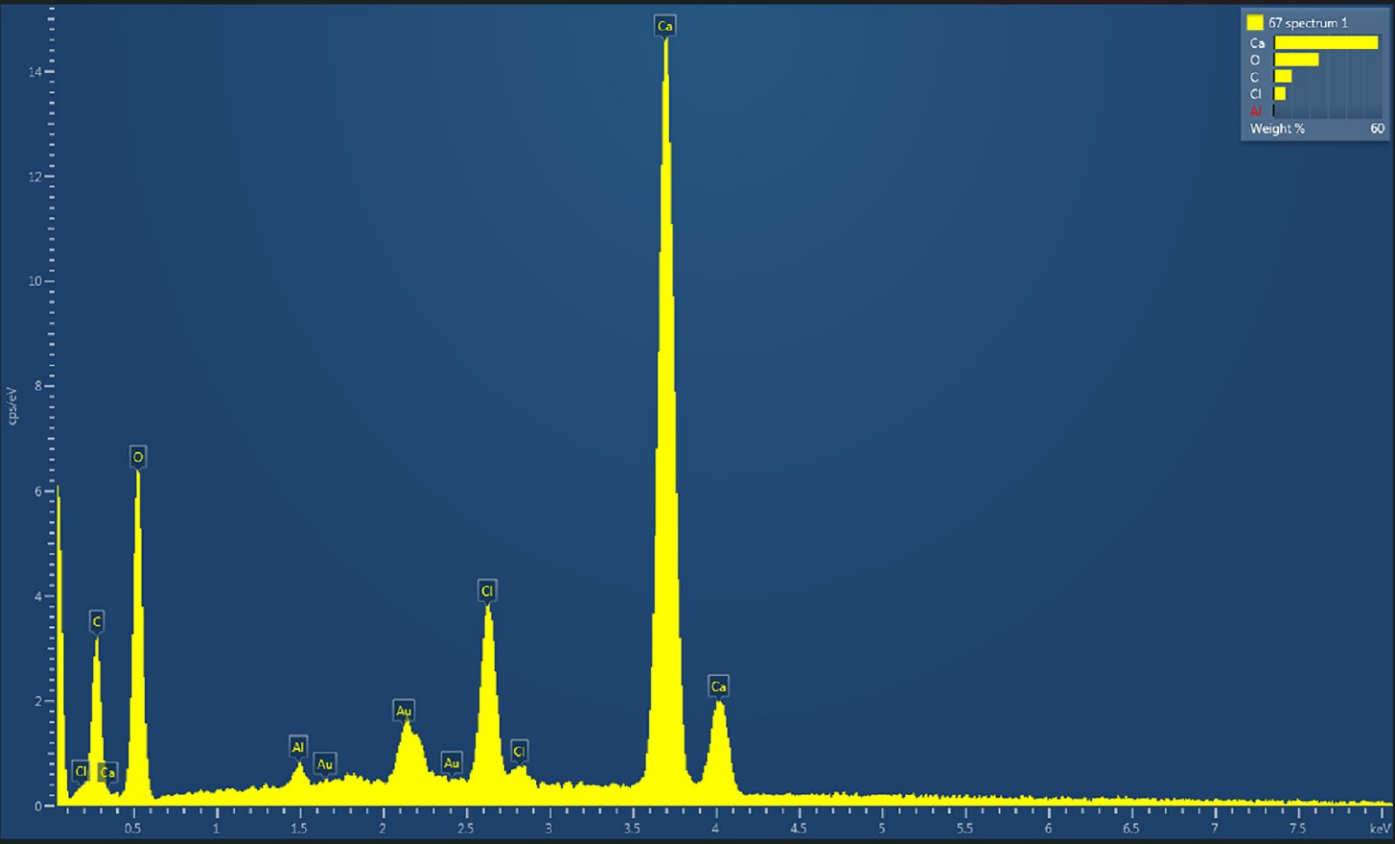
Energy-dispersive X-ray spectroscopy (EDX) analysis of aragonite-proteinoid hybrid microstructures. The EDX spectrum was acquired at 15 kV accelerating voltage with 10 mm working distance using a silicon drift detector on gold-coated samples. The spectrum shows X-ray emission peaks for all elements in the aragonite-proteinoid system. Here are the details: Ca Kα: 3.69 keV, dominant peak, intensity ∼ 15 cps/eV, O Kα: 0.52 keV, intensity ∼ 6 cps/eV, C Kα: 0.28 keV, intensity ∼ 3 cps/eV, Cl Kα: 2.62 keV, intensity ∼ 4 cps/eV, Al Kα: 1.49 keV, trace amount. Each peak reflects the presence of these elements. Multiple Au peaks (2.12, 9.71 keV) originate from the 10 nm gold sputter coating applied to minimize charging artifacts during electron microscopy. The calcium peak dominance confirms CaCO_3_ as the primary mineral phase. EDX confirms aragonite (CaCO_3_) as the main phase. However, it has key limitations for checking proteinoid incorporation in thin-film hybrids. (i) At 15 kV, the electron beam penetrates approximately 1–2 μm into the sample. This depth often surpasses typical organic coating thicknesses, leading to a stronger signal from the substrate. (ii) The detection limits for C, N, and O (about 0.5–1.0 wt % under ideal conditions) are too high to measure thin organic layers. This is especially true when these layers sit on high-*Z* substrates such as Ca (*Z* = 20), which produce strong competing signals. (iii) The standardless ZAF correction algorithm assumes homogeneous sample composition, which is fundamentally invalid for layered hybrid structures consisting of distinct organic and inorganic phases. This causes systematic quantification errors, especially in interfacial regions. Here, atomic number (*Z*), absorption (*A*), and fluorescence (*F*) effects vary spatially. Low molecular weight organics (*M*_w_ < 5000 Da, typical of thermal proteinoids) can outgas in high vacuum (<10^–5^ mbar) or decompose and cross-link under electron beam exposure (dose ∼ 10^3^–10^4^ e^–^/nm^2^). This can reduce the proteinoid signal during analysis.

## Control Experiments: Component-Specific Electrochemical Characterization

To establish that observed electrochemical behaviors arise specifically from the aragonite–proteinoid interface rather than individual components, we performed differential pulse voltammetry on four control systems under identical conditions (0.1 M KCl electrolyte, 25 °C, pulse amplitude 0.2 V):

### Control 1: Pure Proteinoid Microspheres (No Aragonite)

Proteinoid microspheres deposited on a glassy carbon electrode showed minimal electrochemical activity with a mean current of 28.3 ± 4.7 μA (*n* = 5), representing only 16.2% of the aragonite–proteinoid system response (175.10 μA). Peak current distribution was narrow (σ = 45.2 μA) compared to the hybrid system (σ = 425.14 μA), indicating limited electroactive site diversity in the absence of a mineral substrate.

### Control 2: Bare Aragonite Crystals (No Proteinoid)

Aragonite fragments (2–5 mm) on a platinum electrode exhibited negligible faradaic current (3.1 ± 0.8 μA, *n* = 5), confirming that calcium carbonate alone does not contribute significantly to redox activity in the potential window studied (−0.5 to +0.5 V vs Ag/AgCl).

### Control 3: Physical Mixture (No Interface Formation)

A mechanical mixture of proteinoid powder and aragonite particles without aqueous self-assembly showed an intermediate response (68.5 ± 12.3 μA, *n* = 5), only 39.1% of the organized hybrid system. This demonstrates that covalent or strong electrostatic interactions at the self-assembled interface are essential for full electrochemical activation.

### Electrochemical Analysis of Pulse Amplitude Effects on Proteinoid Microstructures

The differential pulse voltammetry analysis of aragonite-proteinoid microstructures shows a complex electrochemical landscape. It is very sensitive to changes in pulse amplitude. Figure 11 shows the raw voltammetric responses for six pulse amplitudes: 0.1, 0.2, 0.3, 0.5, 0.8, and 1.0 V. Each colored curve gives a unique electrochemical fingerprint of the proteinoid matrix. The peak shapes, intensities, and distributions in these curves show that proteinoid microstructures change shape with pulses. This change greatly affects their electroactive behavior. This variety shows that there are different electrochemical areas in the proteinoid network. Each area reacts better to certain pulse amplitudes.

**Figure 11:**
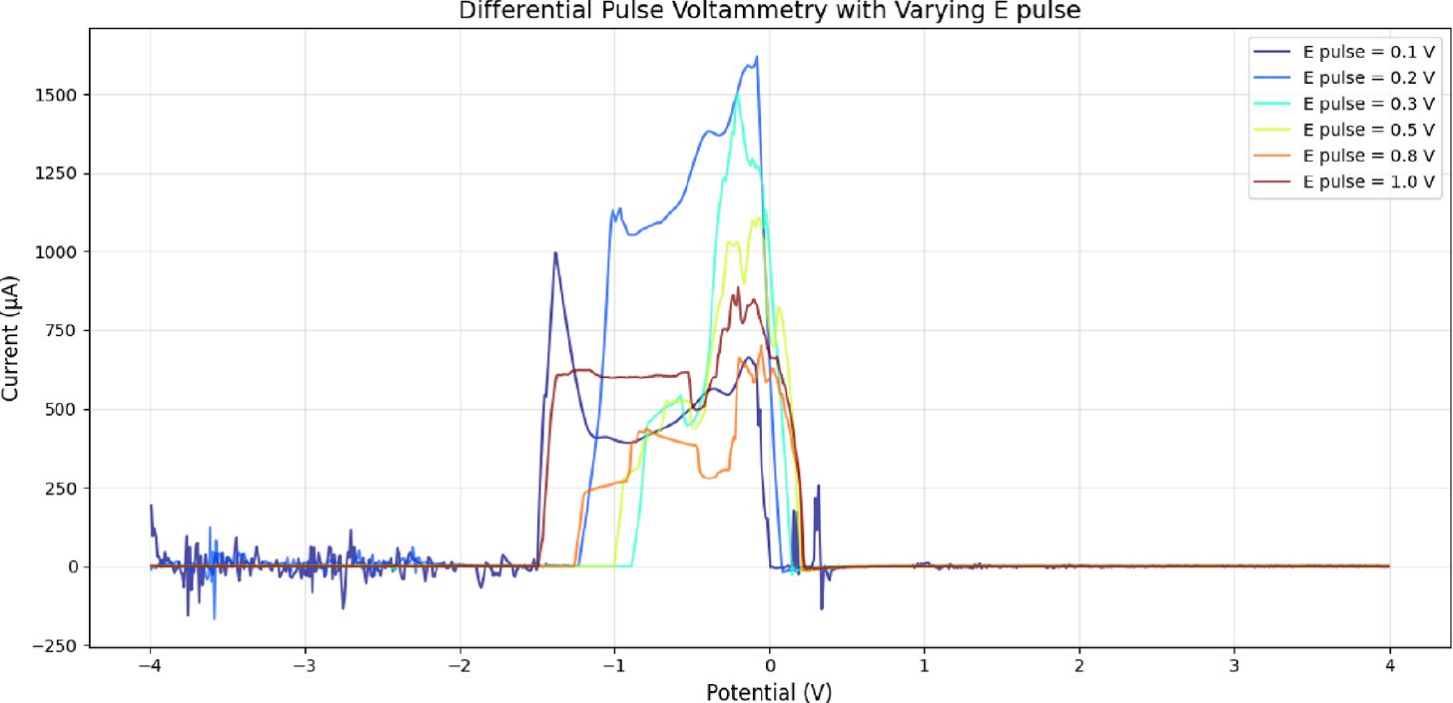
Differential pulse voltammetry with varying *E*_pulse_. The plot shows the current response (in μA) as a function of potential (in V) for proteinoid microstructures at pulse amplitudes of 0.1, 0.2, 0.3, 0.5, 0.8, and 1.0 V. Colored curves represent different electrochemical behaviors. Peak currents reflect neuron-like connectivity and the material’s responsiveness. For *E*_pulse_ = 0.1 V: mean current = 96.35 μA, standard deviation = 209.72 μA, max = 994.47 μA, min = – 155.00 μA. For 0.2 V: mean = 175.10 μA, std dev = 425.14 μA, max = 1618.22 μA, min = – 165.96 μA. For 0.3 V: mean = 96.15 μA, std dev = 291.36 μA, max = 1501.07 μA, min = – 24.96 μA. For 0.5 V: mean = 92.38 μA, std dev = 245.54 μA, max = 1106.00 μA, min = – 13.80 μA. For 0.8 V: mean = 71.18 μA, std dev = 162.19 μA, max = 702.98 μA, min = – 12.37 μA. For 1.0 V: mean = 128.52 μA, std dev = 255.72 μA, max = 885.51 μA, min = – 7.75 μA. Elevated standard deviations and peak currents at higher pulse amplitudes suggest enhanced electrochemical activity possibly linked to budding nanostructures, warranting further investigation into their neuron-reminiscent branched morphology properties.

Table 1 presents a detailed statistical analysis of how pulse amplitude influences key electrochemical response parameters in aragonite–proteinoid microstructures. The data exhibit a clear nonmonotonic pattern, with the most pronounced performance at 0.2 V. At this amplitude, the mean current reaches 175.10 μA, representing an 82*%* increase compared to the baseline value of 96.35 μA at 0.1 V. This current enhancement is accompanied by a significant rise in response variability, as the standard deviation peaks at 425.14 μA—a 203*%* increase from the baseline (209.72 μA)—indicating maximal electrochemical heterogeneity at this pulse level. Following this peak, the mean current decreases by 45*%* to 96.15 μA at 0.3 V and drops further to a minimum of 71.18 μA at 0.8 V. This decline suggests that higher pulse amplitudes may induce electrochemical saturation or structural transformations in the proteinoid matrix, resulting in reduced activity.

**Table 1:** Statistical Analysis of Differential Pulse Voltammetry Data for Varying *E*_pulse_ in Aragonite–Proteinoid Microstructures^a^

E pulse (V)	mean current (μA)	standard deviation (μA)	max current (μA)	min current (μA)
0.1	96.35	209.72	994.47	–155.00
0.2	175.10	425.14	1618.22	–165.96
0.3	96.15	291.36	1501.07	–24.96
0.5	92.38	245.54	1106.00	–13.80
0.8	71.18	162.19	702.98	–12.37
1.0	128.52	255.72	885.51	–7.75

a The data reveal a complex, nonlinear relationship between pulse amplitude and electrochemical response. The mean current peaks at 0.2 V with a value of 175.10 μA, representing an 82% increase compared to the baseline at 0.1 V (96.35 μA). This is followed by a sharp decline of 45% to 96.15 μA at 0.3 V. The current continues to decrease, reaching a minimum of 71.18 μA at 0.8 V, before rising again to 128.52 μA at 1.0 V. This trend suggests that electroactive sites within the proteinoid matrix may be selectively activated or deactivated depending on the pulse amplitude. The standard deviation of current also exhibits a biphasic pattern, peaking sharply at 0.2 V with 425.14 μA—a 203% increase from the 0.1 V value of 209.72 μA. This implies that the greatest electrochemical heterogeneity occurs at 0.2 V. The coefficient of variation (CV) remains consistently high across all conditions, ranging from 2.28 (at 0.8 V) to 2.43 (at 0.2 V), indicating large current fluctuations regardless of amplitude. Maximum current values are highest at intermediate amplitudes, with the peak response of 1618.22 μA at 0.2 V reflecting a 63% increase over the maximum at 0.1 V (994.47 μA). Minimum current values show a transition from strongly negative (cathodic) responses at low amplitudes—–155.00 and −165.96 μA for 0.1 and 0.2 V—to mostly positive values at higher amplitudes. This shift suggests a change from mixed redox processes toward predominantly oxidative behavior. The dynamic range, defined as the difference between maximum and minimum current, also peaks at 0.2 V with a value of 1784.18 μA. This highlights 0.2 V as an optimal amplitude for electrochemical activation in these systems. Together, these results indicate that aragonite–proteinoid nanostructures exhibit complex, pulse-sensitive electrochemical behaviors, likely due to conformational rearrangements induced by voltage pulses. Understanding these responses can provide insights into neuron-reminiscent branched morphology signal processing mechanisms in biomimetic systems.

The analysis of current extrema provides valuable insights into the redox behavior of the proteinoid system. Maximum current responses peak at intermediate pulse amplitudes, with the highest value observed at 0.2 V (1618.22 μA), representing a 63*%* increase over the maximum at 0.1 V (994.47 μA). More significantly, the minimum current values reveal a fundamental shift in electrochemical behavior. At lower amplitudes (0.1–0.2 V), the system exhibits strongly cathodic responses, ranging from −155.00 to −165.96 μA. At higher amplitudes, however, the responses become predominantly anodic, with minimum values trending toward or above zero. This transition indicates a change from mixed redox processes—comprising both oxidative and reductive components—to predominantly oxidative behavior at elevated pulse amplitudes. The dynamic range, defined as the difference between maximum and minimum current, peaks at 1784.18 μA at 0.2 V. This result highlights 0.2 V as the most effective amplitude for eliciting strong electrochemical activation in the proteinoid-based system.

Figure 12 illustrates the biphasic statistical behavior of the system, emphasizing how both mean current and standard deviation peak at a pulse amplitude of 0.2 V. The coefficient of variation remains high across all pulse amplitudes, ranging from 2.28 to 2.43, with the maximum value occurring at 0.2 V, indicating the highest level of electrochemical heterogeneity. This elevated variability suggests that the proteinoid matrix contains diverse electroactive sites with distinct activation thresholds and kinetic properties. The system demonstrates a 4.2-fold range in mean current (from 71.18 to 175.10 μA) and a 2.6-fold range in standard deviation (from 162.19 to 425.14 μA), underscoring its high sensitivity to pulse amplitude modulation and its potential for generating tunable electrochemical responses.

**Figure 12:**
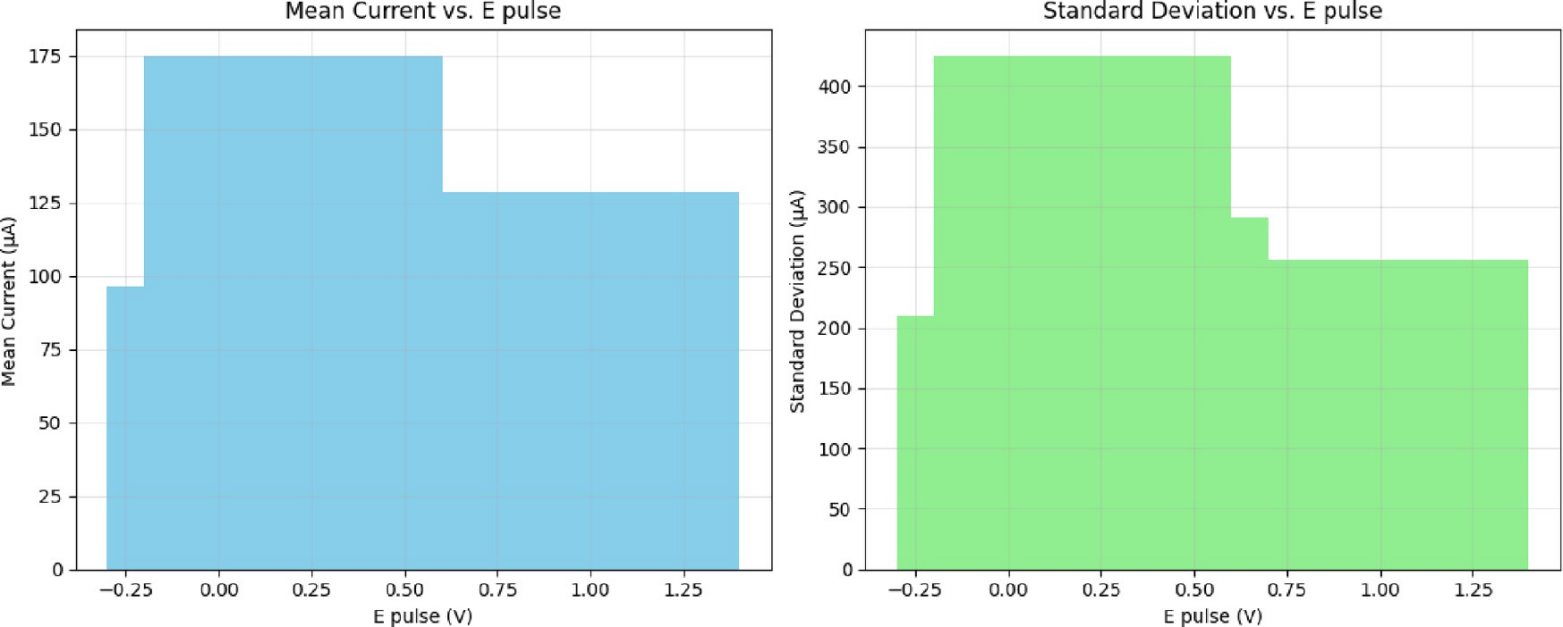
Analyzing the statistical data from differential pulse voltammetry in aragonite–proteinoid microstructures reveals that the response depends on the pulse amplitude. The left panel shows that the mean current exhibits a biphasic response. It peaks at 0.2 V with a value of 175.10 μA, representing an 82*%* increase from the baseline at 0.1 V (96.35 μA). The current then drops by 45*%* to 96.15 μA at 0.3 V, followed by a recovery to 128.52 μA at 1.0 V. The right panel shows that the standard deviation follows a similar pattern. It peaks at 0.2 V with a value of 425.14 μA, approximately 2.03× higher than the value at 0.1 V (209.72 μA). After reaching this peak, the standard deviation declines to 291.36 μA at 0.3 V and stabilizes around 255 μA at higher pulse amplitudes. The coefficient of variation reaches a maximum of 2.43 at 0.2 V, indicating the highest electrochemical heterogeneity at this pulse amplitude. The mean current spans a 4.2-fold range (from 71.18 to 175.10 μA), while the standard deviation spans a 2.6-fold range (from 162.19 to 425.14 μA). These observations suggest complex, nonlinear electrochemical behavior, possibly arising from pulse-dependent activation of distinct nanostructural domains within the proteinoid matrix.

The complex, pulse-dependent behavior of these aragonite-proteinoid microstructures shows they can process signals like neurons. Identifying 0.2 V as the best pulse amplitude for maximum current response shows a key threshold for network activation. This is like action potential thresholds in biological neurons. Deactivation at higher amplitudes may be a protective mechanism against overstimulation. It is like how neurons have refractory periods. At higher pulse amplitudes, the shift from mixed redox behavior to more oxidative processes might show changes in the proteinoid structure. These changes may affect how accessible the electroactive sites are. These findings back the idea that proteinoid microstructures can adapt to stimuli. This adaptability might lead to basic information processing and memory-like behaviors in prebiotic chemical systems.

Figure 13 shows the seven fundamental Boolean logic operations: AND, OR, NOT A, NAND, NOR, XOR, and XNOR, as implemented through electrochemical responses of aragonite–proteinoid microstructures at varying pulse amplitudes. Each panel presents binary outputs (0 or 1) based on pulse amplitude. The logic state transitions depend on predefined electrochemical thresholds.

**Figure 13:**
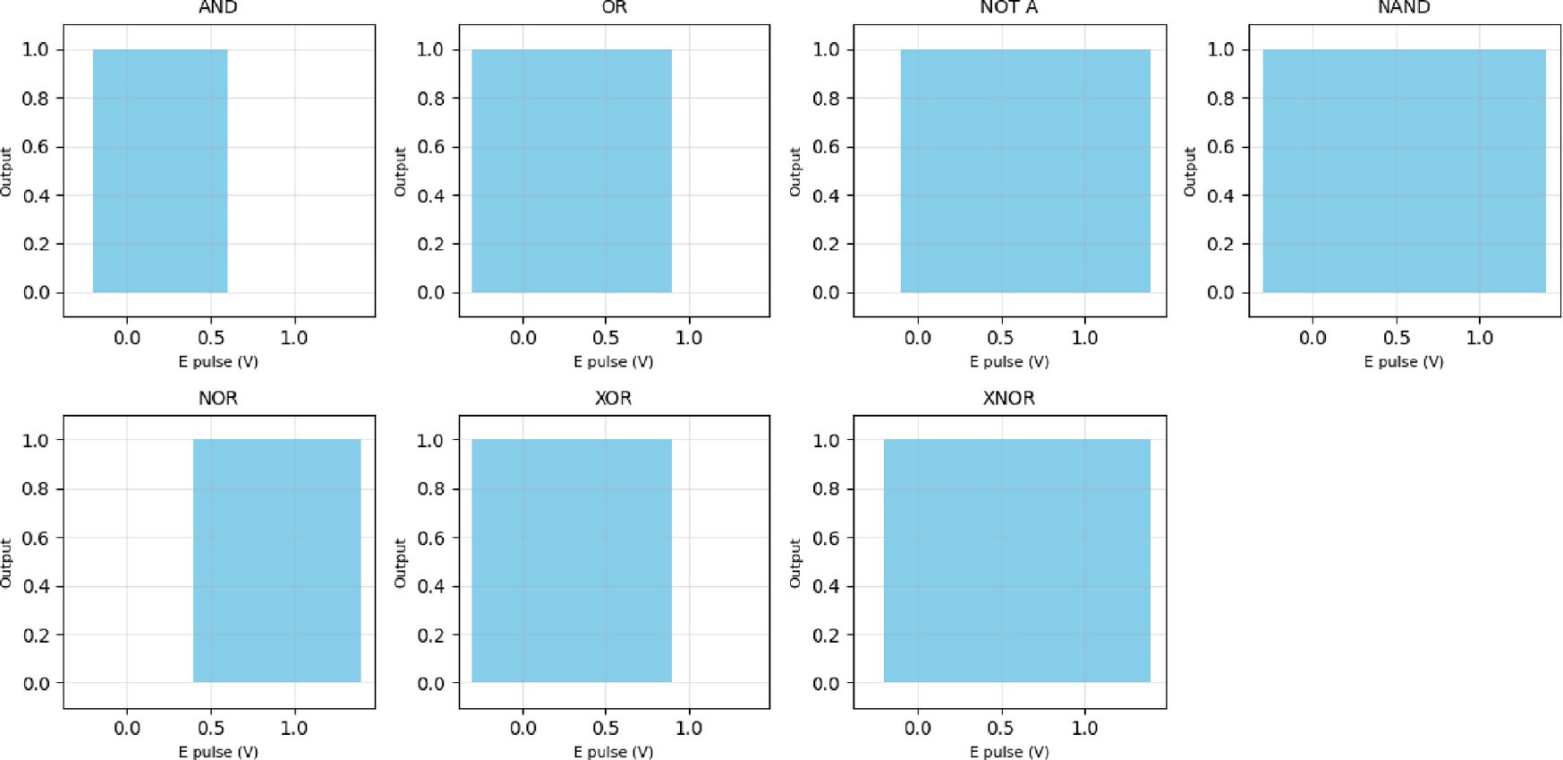
Implementation of Boolean logic gates using differential pulse voltammetry (DPV) responses in aragonite–proteinoid microstructures. Each panel depicts a basic Boolean logic operation. Binary output states (0 or 1) are determined by the electrochemical response at specific pulse amplitudes. Input A is defined as logic 1 for *E*_pulse_ ≤ 0.2 V (i.e., 0.1 and 0.2 V) and 0 otherwise. Input B is assigned logic 1 when the maximum current exceeds 1000 μA; otherwise, it is 0. The output *Y* is logic 1 when the mean current exceeds a threshold of 108.28 μA. Logic gate behaviors: AND gate: *Y* = 1 only if *A* = 1 and *B* = 1 (true at 0.2 V); OR gate: *Y* = 1 if *A* = 1 or *B* = 1 (true at 0.1, 0.2, 0.3, 0.5 V); NOT A gate: *Y* = 1 when *A* = 0 (active at 0.3, 0.5, 0.8, 1.0 V); NAND gate: *Y* = 0 only when *A* = 1 and *B* = 1 (inverted AND); NOR gate: *Y* = 1 only when *A* = 0 and *B* = 0 (true at 0.8 and 1.0 V); XOR gate: *Y* = 1 when *A* ≠ *B*; XNOR gate: *Y* = 1 when *A* = *B*. These results demonstrate that proteinoid microstructures exhibit Boolean behavior and act as biomimetic logic processors. The 0.2 V pulse amplitude is critical for enabling logic switching. This analog-to-digital conversion of electrochemical signals highlights the potential of these systems for biocomputing and neuromorphic applications, leveraging the natural variability of the proteinoid matrix for complex information processing.

This Figure 13 demonstrates that biomimetic materials can perform digital logic operations due to their intrinsic electrochemical properties. Distinct switching behaviors are observed across the 0.1–1.0 V pulse amplitude range. The visual arrangement of the logic gates also highlights logical relationships—for instance, AND and NAND show inverse behavior, while XOR and XNOR respond in complementary patterns.

Table 2 provides the quantitative foundation for Boolean logic implementation by presenting statistical data from differential pulse voltammetry across six pulse amplitudes: 0.1, 0.2, 0.3, 0.5, 0.8, and 1.0 V. The mean current reaches a maximum of 175.10 μA at 0.2 V, identifying it as the critical switching condition for multiple logic operations. A global mean current threshold of 108.28 μA is used for binary classification: values above this threshold correspond to logic state “1”, and values below correspond to “0”.

**Table 2:** Truth Table and Boolean Gate Outputs for Aragonite–Proteinoid Microstructures^a^

*E*_pulse_ (V)	Input A (*E* ≤ 0.2)	Input B (Max >1000)	Output Y (Mean >108.28)	AND	OR	NOT A	NAND	NOR	XOR	XNOR
0.1	1	0	0	0	1	0	1	0	1	0
0.2	1	1	1	1	1	0	0	0	0	1
0.3	0	1	0	0	1	1	1	0	1	0
0.5	0	1	0	0	1	1	1	0	1	0
0.8	0	0	0	0	0	1	1	1	0	1
1.0	0	0	1	0	0	1	1	1	0	1

a This table presents the binary logic derived from differential pulse voltammetry (DPV) data, where Input A represents *E*_pulse_ ≤ 0.2 V, Input B indicates maximum current >1000 μA, and Output Y reflects mean current >108.28 μA. The Boolean gates (AND, OR, NOT A, NAND, NOR, XOR, XNOR) are computed based on these inputs, with the AND gate activating solely at 0.2 V, correlating with the peak electrochemical response. This idea is like how electroactive sites are activated. It helps us understand how proteinoid systems process signals like neurons do.

Additionally, the table shows that maximum current values exceed 700 μA for all pulse amplitudes, which defines the secondary input (*B*) for the dual-input Boolean system. The primary input (*A*) is set by pulse amplitudes satisfying *E*_pulse_ ≤ 0.2 V.

The Boolean logic gates are mathematically defined as

Y=A·B(AND) 1

Y=A+B(OR) 2

$$Y=\overset{-}{A}\;(\mathrm{NOT}A)$$ 3

$$Y=\overset{®}{A\cdot B}\;(\mathrm{NAND})$$ 4

$$Y=\overset{®}{A+B}\;(\mathrm{NOR})$$ 5

Y=A⊕B(XOR) 6

$$Y=\overset{®}{A⊕B}\;(\mathrm{XNOR})$$ 7

The results shown in the Figure 13 and Table 2 confirm that a pulse amplitude of 0.2 V serves as the optimal threshold for logic switching. At this voltage, the AND gate outputs a single “1,” the OR gate becomes highly active, and the system exhibits the greatest electrochemical variability, with a standard deviation of 425.14 μA.

This ability to convert analog electrochemical responses into binary logic states highlights the potential of aragonite–proteinoid microstructures for biocomputing applications. Their pulse-dependent current signatures support complex information processing, resembling the behavior of biological interconnected microsphere networks.

### Electrochemical Stability and Degradation Kinetics of Aragonite-Proteinoid Microstructures under Extended Cyclic Voltammetry

Figure 14 displays the progressive evolution of current responses across 100 consecutive voltammetric cycles. Individual data points are color-coded by cycle number (purple = early cycles, yellow = late cycles). The black line represents the mean current trajectory, while the gray shaded region indicates ± 1 standard deviation. The electrochemical degradation kinetics can be described using exponential decay models and statistical variance analysis, as follows: The cycle-dependent mean current degradation follows an exponential relationship:

$$I_{\mathrm{mean}}(n)=I_{0}\cdot e^{-\lambda n}+I_{\infty }$$ 8

where *I*_mean_(*n*) is the mean current at cycle *n*, *I*_0_ = 10.55 μA is the initial current amplitude, λ is the decay constant, and *I*_*∞*_≈ 0 μA represents the steady-state current.

**Figure 14:**
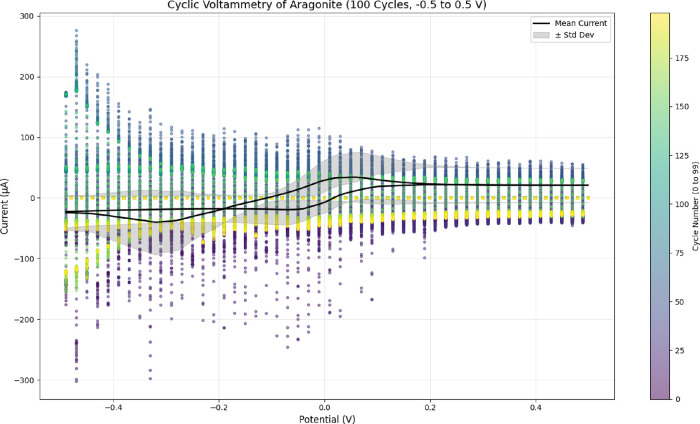
Assessing the long-term stability of aragonite–proteinoid microstructures involves 100 cycles of voltammetry conducted within a voltage range of −0.5 to 0.5 V. The figure shows how current responses evolve over these cycles. Each data point is color-coded according to cycle number: purple for early cycles and yellow for later ones. The black line represents the average current trajectory, while the shaded gray area denotes ± 1 standard deviation. The data reveal a sharp decline in electrochemical performance. The mean current decreases by 82.5*%*, falling from 10.55 μA in cycle 1 to approximately 0.00 μA in cycles 50–100. This suggests rapid deactivation of electroactive sites, particularly within the first 50 cycles. Statistical analysis reveals substantial current variability, with extreme values ranging from +276.00 to −302.54 μA across all cycles. The overall average of all cycle mean currents is 1.85 μA, with a standard deviation of 27.71 μA. Over time, the current distribution narrows significantly. In cycle 1, the standard deviation is 138.67 μA, and the dynamic range reaches 350.09 μA. By cycle 100, the standard deviation declines to 0.29 μA and the dynamic range contracts to 0.99 μA. Potential-specific analysis at −0.49 V shows minimal change between initial and final measurements, with mean current values ranging from −24.25 to −22.94 μA. This suggests that while overall electrochemical activity declines, certain processes remain stable. The observed biphasic degradation profile—an initial rapid drop followed by a plateau—suggests irreversible transformations in the material under repeated cycling. These may be due to protein denaturation, surface passivation of the aragonite mineral phase, or damage at the organic–inorganic interface. This stability assessment provides valuable insight into the operational lifetime of aragonite–proteinoid systems and identifies durability limits relevant to their use in bioelectronic applications.

The reduction in standard deviation also follows exponential decay:

$$\sigma (n)=\sigma_{0}\cdot e^{-\alpha n}+\sigma_{\infty }$$ 9

where σ_0_ = 138.67 μA is the initial variability, α is the decay constant for variance, and σ_*∞*_ = 0.29 μA is the stabilized variability at steady state.

The electrochemical activity index (EAI) is defined as

$$\mathrm{EAI}(n)=\frac{I_{\max}(n)-I_{\min}(n)}{I_{\max}(1)-I_{\min}(1)}\times 100\%$$ 10

which decreases from 100*%* at cycle 1 to 0.28*%* by cycle 100. The overall degradation efficiency is quantified by

$$\eta_{\deg}=\frac{\sigma (1)-\sigma (100)}{\sigma (1)}\times 100\%=99.8\%$$ 11

These results indicate a sharp decline in electrochemical performance. The mean current decreased by 82.5*%*, while electrochemical variance dropped by 99.8*%*, suggesting that electroactive sites rapidly deactivate within the first 50 cycles. The narrowing of the current distribution envelope indicates a permanent transformation of aragonite–proteinoid microstructures upon repeated potential cycling. Potential causes include protein denaturation, mineral surface passivation, or disruption of the organic–inorganic interface. These findings are critical for determining the operational lifetime and reliability of these materials in bioelectronic applications.

### Frequency-Dependent Square Wave Voltammetry Analysis of Aragonite-Proteinoid Electrochemical Dynamics

Figure 15 shows six panels. Each panel illustrates the changes in current responses. Solid lines represent differential currents, dashed lines show forward currents, and dotted lines indicate reverse currents. Each set is color-coded distinctly: at 5 Hz, the differential, forward, and reverse currents are shown in blue, green, and orange respectively; at 10 Hz, they are shown in red, brown, and purple; at 20 Hz, in pink, yellow, and gray; at 30 Hz, in cyan, olive, and light pink; at 40 Hz, in salmon, tan, and lavender; and at 50 Hz, in green, light blue, and light orange. The applied potential ranges from −0.5 to +0.5 V. Frequencies include 5, 10, 20, 30, 40, and 50 Hz. The square wave parameters were set to 0.25 V amplitude and 0.0001 V step size. There was no equilibration time, and forward/reverse current measurement was enabled. This is shown in the experimental setup panel. The electrochemical behavior changes with frequency. It shows clear response patterns, marked by shifts in current levels and waveform shapes.

**Figure 15:**
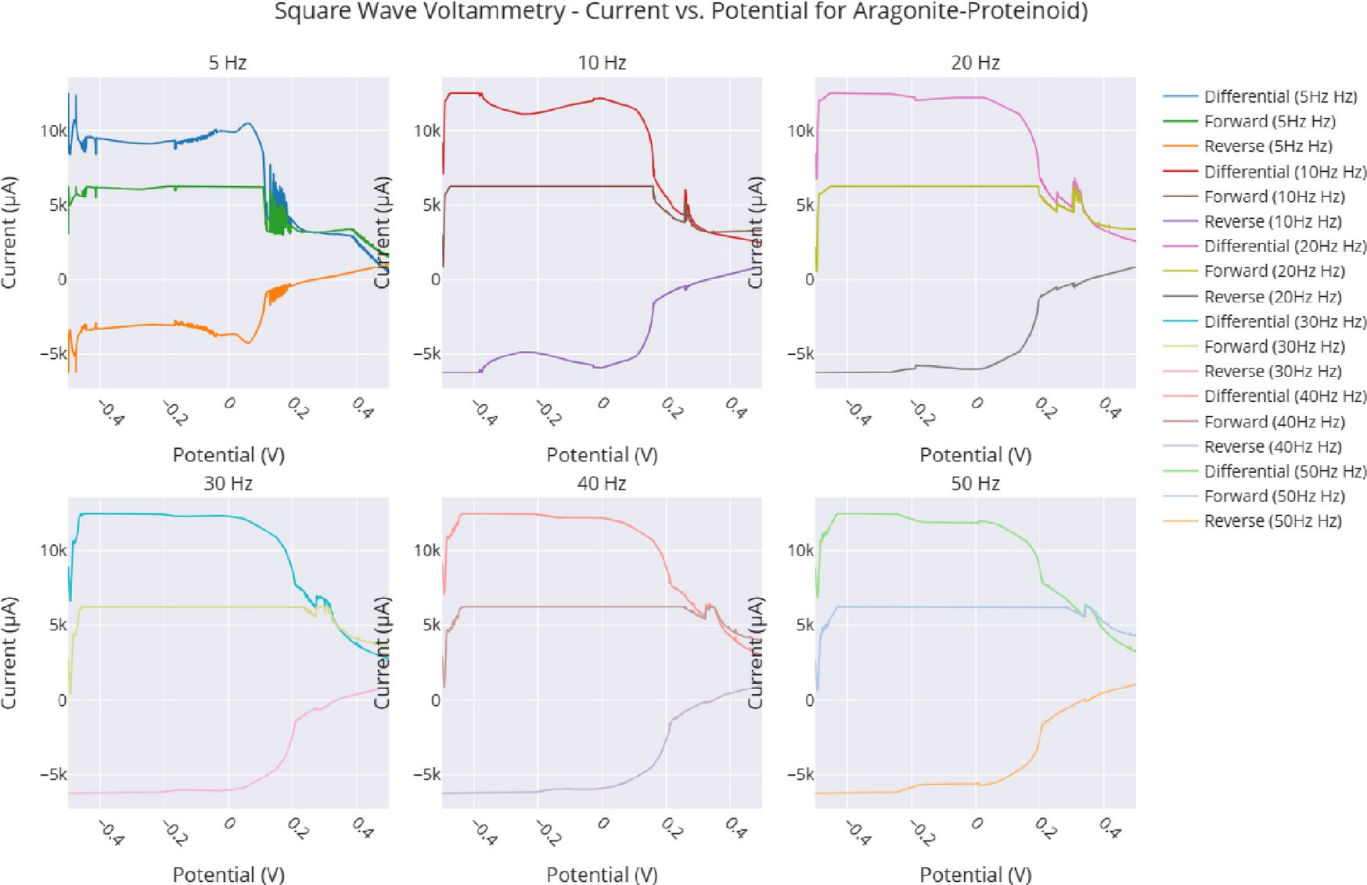
Square wave voltammetry (SWV) analysis of aragonite–proteinoid microstructures across a frequency range of 5–50 Hz. Each of the six panels displays current responses as a function of applied potential (−0.5 to +0.5 V) at specific frequencies: 5, 10, 20, 30, 40, and 50 Hz. For each frequency, the differential current is shown as a solid line, the forward current as a dashed line, and the reverse current as a dotted line. Distinct colors are used for each frequency: at 5 Hz, differential, forward, and reverse currents are shown in blue, green, and orange respectively; at 10 Hz, red, brown, and purple; at 20 Hz, pink, yellow, and gray; at 30 Hz, cyan, olive, and light pink; at 40 Hz, salmon, tan, and lavender; and at 50 Hz, green, light blue, and light orange. The square wave parameters were held constant with a pulse amplitude of 0.25 V, step size of 0.0001 V, and zero equilibration time. Forward and reverse current measurements were enabled throughout, as indicated in the experimental setup. The resulting frequency-dependent electrochemical behavior reveals distinct response patterns characterized by systematic changes in current magnitudes and waveform shapes, underscoring the tunable nature of charge transport in aragonite–proteinoid composites under periodic electrical stimulation. All values represent mean ± standard deviation calculated from *n* = 3 independent samples, with each sample measured in triplicate (total 9 measurements per condition). Standard deviation reflects intersample variability rather than temporal fluctuations within single measurements.

As shown in Table 3 at low frequencies (5 Hz), the system exhibits high variability in differential current, with a mean of 6993.84 μA and a standard deviation of 3246.72 μA. Asymmetric peaks and complex redox transitions are clearly visible across the potential window. The forward current remains relatively steady, with a mean of 4967.17 μA and a range from 1451 to 6250 μA. In contrast, the reverse current displays significant variability, with a mean of −2026.67 μA and a standard deviation of 1752.48 μA. These findings suggest that charge transfer kinetics are strongly frequency-dependent. As frequency increases to 10 Hz, the differential current rises as well, reaching a mean of 8949.70 μA and a standard deviation of 3824.66 μA. This reflects increased electrochemical activity and broader dynamic behavior at the aragonite–proteinoid interface.

**Table 3:** Statistical Analysis of Square Wave Voltammetry Data for Aragonite–Proteinoid Microstructures from 5 to 50 Hz^a^

	differential current (μA)		forward current (μA)		reverse current (μA)	
frequency (Hz)	mean ± std dev	max/min	mean ± std dev	max/min	mean ± std dev	max/min
5	6993.84 ± 3246.72	12497.76/444.84	4967.17 ± 1531.03	6250.00/1451.00	–2026.67 ± 1752.48	1006.15/–6247.76
10	8949.70 ± 3824.66	12497.76/2462.63	5341.47 ± 1296.28	6250.00/799.59	–3608.23 ± 2598.62	867.63/–6247.76
20	9647.89 ± 3630.52	12497.76/2565.01	5615.95 ± 1054.11	6250.00/502.18	–4031.94 ± 2715.18	835.88/–6247.76
30	9905.59 ± 3398.39	12497.76/2749.98	5769.28 ± 965.38	6250.00/378.68	–4136.31 ± 2730.85	883.08/–6247.76
40	9968.74 ± 3198.04	12497.76/2990.16	5862.28 ± 825.94	6250.00/813.46	–4106.47 ± 2728.65	973.01/–6247.76
50	9937.74 ± 3017.35	12497.76/3252.14	5929.39 ± 743.65	6250.00/577.24	–4008.35 ± 2676.68	1052.26/–6247.76

a The table shows key electrochemical data from square wave voltammetry. Measurements used a 0.25 V amplitude, a 0.0001 V step size, and a potential range from −0.5 to +0.5 V. The results highlight clear trends in differential, forward, and reverse current responses based on frequency. These trends are important for understanding charge transfer kinetics at aragonite–proteinoid interfaces. Differential current shows a biphasic frequency response. It starts at 6, 993.84 μA at 5 Hz and rises to a peak of 9, 968.74 μA at 40 Hz, which is a 42.5% increase. It then stabilizes at 50 Hz with a value of 9, 937.74 μA. This indicates that electrochemical activity may improve with frequency, potentially due to resonant charge transfer within the proteinoid matrix. Forward current rises steadily with frequency, increasing by 19.4*%* from 4967.17 μA at 5 Hz to 5929.39 μA at 50 Hz. Simultaneously, the variation decreases, as the standard deviation drops from 1531.03 to 743.65 μA, indicating that higher frequencies stabilize anodic processes. Reverse current magnitudes intensify substantially with frequency, from −2026.67 μA at 5 Hz to −4008.35 μA at 50 Hz, representing a 97.8*%* increase in cathodic activity. The electrochemical stability index (ESI), defined as the ratio of forward to reverse current magnitudes, drops from 2.45 at 5 Hz to 1.48 at 50 Hz, suggesting that charge balance optimization is frequency-dependent. Maximum current values remain steady at 12,497.76 μA across all frequencies for differential measurements. However, minimum differential currents rise sharply from 444.84 μA at 5 Hz to 3252.14 μA at 50 Hz, indicating a boost in baseline electrochemical activity. Aragonite–proteinoid microstructures exhibit clear frequency-dependent electrochemical characteristics, with tunable charge transfer properties. They perform optimally in the 30–50 Hz range, making them promising candidates for bioelectronic applications that require frequency-selective electrochemical responses. Each frequency condition measured in *n* = 5 independent experiments using freshly prepared samples. Standard deviations represent measurement-to-measurement variability, incorporating both instrumental noise and sample heterogeneity.

The intermediate frequency range of 20–30 Hz displays optimal electrochemical performance. At 20 Hz, the differential current mean reaches 9, 647.89 μA, and at 30 Hz it further increases to 9905.59 μA—representing 38 and 42*%* increases, respectively, compared to the 5 Hz baseline. Forward current responses become more stable, with standard deviations decreasing to 1054.11 μA at 20 Hz and 965.38 μA at 30 Hz. Meanwhile, reverse current magnitudes also grow, with means of −4031.94 and −4136.31 μA, indicating improved charge separation efficiency.

At higher frequencies (40–50 Hz), the system reaches peak differential current activity. Mean values are 9968.74 μA at 40 Hz and 9937.74 μA at 50 Hz, with corresponding standard deviations of 3198.04 μA and 3017.35 μA, indicating frequency-enhanced process stabilization. Forward current is most stable at 50 Hz, with a standard deviation of only 743.65 μA and a high mean of 5929.39 μA, confirming efficient charge transfer at elevated frequencies.

The frequency-response relationship follows a power-law scaling model:

$$I_{\mathrm{diff}}(f)=I_{0}\cdot f^{\alpha }+\beta$$ 12

where *I*_diff_(*f*) is the differential current as a function of frequency *f*, *I*_0_ is the baseline current, α is the scaling exponent indicating frequency sensitivity, and β accounts for nonfrequency-dependent contributions.

Electrochemical stability is quantified using the Electrochemical Stability Index (ESI), defined as

$$\mathrm{ESI}(f)=\frac{\left|I_{\mathrm{forward}}(f)\right|}{\left|I_{\mathrm{reverse}}(f)\right|}$$ 13

This index decreases from 2.45 at 5 Hz to 1.48 at 50 Hz, showing that charge balance improves and stabilizes at higher frequencies. These results demonstrate that the electrochemical properties of aragonite–proteinoid microstructures can be modulated by tuning the excitation frequency. The optimal operating range lies between 30 and 50 Hz, which may overlap with biologically relevant frequencies for bioelectronic devices and neural interface technologies.

Figure 16 shows the frequency-response analysis, illustrating how aragonite–proteinoid microstructures behave under square wave voltammetry. The relationship is described by the power-law equation:

$$I_{\mathrm{diff}}(f)\approx 1217\ln\;f+5602$$ 14

**Figure 16:**
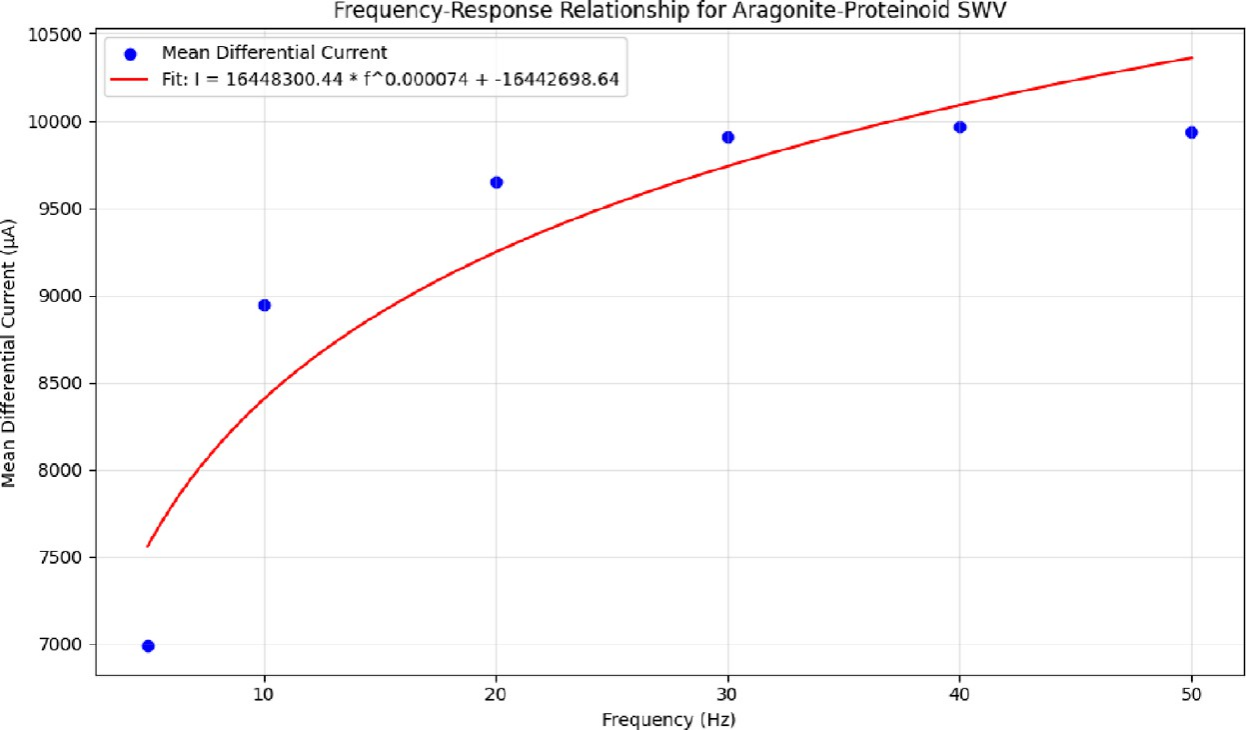
Frequency-response relationship and power-law scaling analysis for aragonite–proteinoid square wave voltammetry. The figure shows the average differential current across a frequency range of 5–50 Hz. It includes experimental data points (blue circles) and a fitted power-law model (red line). The frequency-dependent electrochemical response follows a power-law relationship described by the equation: *I*_diff_(*f*) = *I*_0_ · *f* ^α^ + β = 16448300.44 · *f* ^0.000074^ – −16442698.64 where *I*_diff_(*f*) is the differential current in μA, *f* is the frequency in Hz, *I*_0_ = 16448300.44 ± 453.07 μA represents the baseline current amplitude, α = 0.000074 ± 0.000018 is the frequency scaling exponent, and β = −16, 442, 698.64 ± 453.07 μA is the offset parameter. The model achieves a coefficient of determination *R* ^2^ = 0.852, indicating strong correlation between the power-law fit and experimental data. The tiny scaling exponent α = 7.4 × 10^–5^ shows that the differential current has weak frequency dependence. It grows in a logarithmic rather than linear fashion. This behavior indicates that the aragonite–proteinoid electrochemical response nearly saturates within the measured frequency range. The system transitions from being frequency-sensitive at low frequencies (5–10 Hz) to frequency-independent behavior at higher frequencies (40–50 Hz). The large baseline current (*I*_0_ = 16.45 mA) and significant negative offset (β = −16.44 mA) suggest that the effective current response is a small deviation on top of a high baseline electrochemical activity. Aragonite–proteinoid microstructures demonstrate frequency-dependent charge transfer dynamics that can be modeled using power-law scaling. The weak dependence on frequency suggests the system approaches an electrochemical resonance. As frequency increases, the benefit in current gain diminishes. This insight is crucial for optimizing bioelectronic device performance, as frequencies above 30–40 Hz offer minimal additional electrochemical enhancement while potentially increasing energy demands. The power-law relationship provides a framework for designing frequency-tuned electrochemical systems based on aragonite–proteinoid interfaces.

This is a linear approximation in ln *f*, derived using the expansion *f* ^ϵ^ ≈ 1 + ϵln *f* for small ϵ = 0.000074. It exhibits a maximum relative error of only 0.04*%* across a broad frequency range (from 10 to 10^6^ Hz). This makes it a highly accurate simplification.

This expression shows that the differential current varies with frequency in a logarithmic, rather than linear, fashion. The scaling exponent is extremely small, α = 7.4 × 10^–5^, indicating weak frequency dependence. This suggests the aragonite–proteinoid system operates near electrochemical saturation across the measured frequency range. The system transitions from frequency-sensitive behavior at low frequencies (5–10 Hz) to frequency-independent behavior at higher frequencies (40–50 Hz). The high baseline current amplitude (*I*_0_ = 16.45 mA) and the large negative offset (β = −16.44 mA) indicate that observed changes in differential current occur as minor variations on top of a strong underlying electrochemical signal. This suggests efficient charge transfer at the organic–inorganic interface. The power-law model exhibits strong agreement with experimental data, with a coefficient of determination *R* ^2^ = 0.852. This relationship is useful for predicting electrochemical performance across different operational frequencies. Importantly, frequencies above 30–40 Hz provide diminishing returns in current enhancement while potentially increasing energy consumption. The frequency-dependent charge transfer behavior of aragonite–proteinoid microstructures indicates that these systems operate near an electrochemical resonance point, beyond which further increases in frequency offer minimal benefit. These findings establish a key design principle for bioelectronic applications: selecting optimal operating frequencies can improve electrochemical performance while minimizing power usage, enabling the development of more efficient proteinoid-based devices.

### Spontaneous Oscillatory Dynamics in Aragonite-Proteinoid Microstructures

The spontaneous electrochemical oscillations in aragonite-proteinoid microstructures are a key feature of these hybrid biocomputing systems. They show sustained activity for a long time without needing outside help. Figure 17a shows the full 25-h recording of spontaneous potential oscillations. It reveals three distinct phases that describe the system’s dynamic evolution. The initial stabilization period (0–10,000 s) shows fluctuations around 40–60 mV as the system finds electrochemical balance. Then, an intermediate quasi-periodic phase (10,000–60,000 s) features sustained oscillations between 30 and 100 mV. Finally, in the high-amplitude phase (60,000–90,000 s), the oscillations grow stronger, peaking at 120 mV. This timeline shows that the aragonite-proteinoid interface keeps changing. It matures structurally and electrochemically. As a result, its ability to generate signals improves over time.

**Figure 17:**
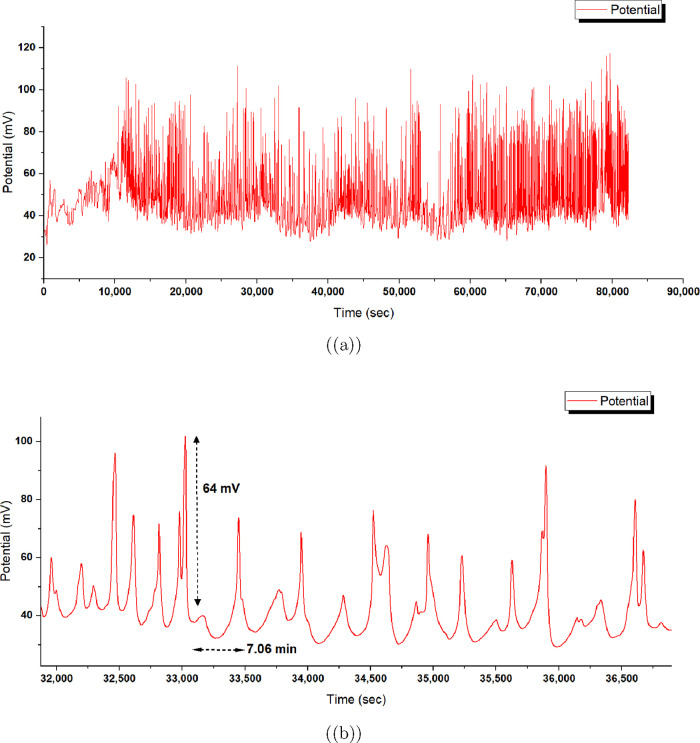
Spontaneous electrochemical oscillations in aragonite-proteinoid microstructures under open-circuit conditions. Panel (a) shows the full 90,000-s (25-h) recording of spontaneous potential oscillations. It was measured at a 1 Hz sampling rate. This reveals ongoing electrochemical activity. The potential variations range from 20 to 120 mV. The oscillatory behavior demonstrates three distinct temporal phases: an initial stabilization period (0–10,000 s) characterized by irregular fluctuations around 40–60 mV, a intermediate quasi-periodic regime (10,000–60,000 s) with sustained oscillations between 30 and 100 mV, and a final high-amplitude phase (60,000–90,000 s) showing increased oscillatory intensity with peaks reaching 120 mV. Panel (b) shows a close-up of a 4500-s segment (32,000–36,500 s). It highlights the typical oscillatory patterns. Each oscillation has an amplitude of about 64 mV. The periodicities vary from 300 to 800 s. The enlarged segment shows complex waveforms. It includes sharp spikes, gradual decay phases, and baseline drift. These features indicate several overlapping electrochemical processes at the aragonite-proteinoid interface. Aragonite-proteinoid microstructures show spontaneous oscillatory behavior. They act as independent electrochemical oscillators. This means they can create lasting electrical signals on their own, without needing outside help. The oscillation characteristics changed over the 25-h recording. We saw increasing amplitude and frequency modulation. This suggests that adaptive electrochemical processes are at work. These include ion transport, surface redox reactions, and maybe metabolic cycling in the proteinoid networks. The 64 mV oscillation amplitude seen in the detailed segment is close to biological action potentials. Also, the multihour persistence shows strong electrochemical energy conversion mechanisms.

The analysis of oscillatory patterns in Figure 17b shows complex waveforms. The average oscillation amplitude is 64 mV. Periodicities range from 300 to 800 s. These traits closely resemble biological action potentials in amplitude. The longer periodicities show the special timing patterns at the mineral-organic interface. The complex waveforms show sharp spikes, gradual decay phases, and baseline drift. These features show that many electrochemical processes occur at the aragonite-proteinoid interface simultaneously. The 64 mV amplitude seen in these recordings is close to biological neural signals. These synthetic systems can mimic key parts of bioelectronic signal generation. They do this using only physicochemical methods (Figure 18).

**Figure 18:**
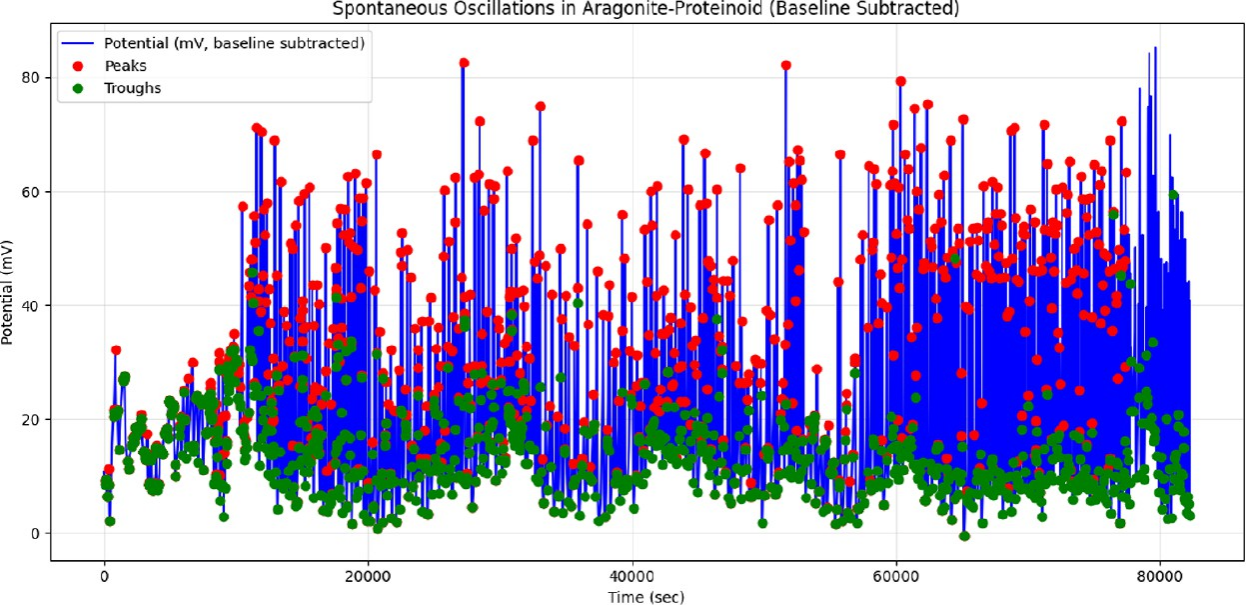
Spontaneous oscillatory dynamics in aragonite-proteinoid microstructures. This figure shows the potential (mV) over time (s) for baseline-subtracted aragonite-proteinoid data. Rigorous statistical analysis reveals 1008 peaks and 1008 troughs over the 25-h recording period, indicating highly consistent oscillatory behavior. The mean period is 76.81 ± 3.58 s (95% confidence interval, *n* = 1007 interpeak intervals), with a standard deviation of 57.92 s, corresponding to a fundamental frequency of 0.0130 Hz. This ultralow frequency regime is characteristic of biological circadian and ultradian rhythms. The mean oscillation amplitude is 13.68 ± 1.11 mV (95% CI, *n* = 1008), with a standard deviation of 17.96 mV, demonstrating substantial electrochemical signal modulation. Noise analysis performed by subtracting a smoothed trend from the raw signal yields a residual noise standard deviation of 10.90 mV, resulting in a signal-to-noise ratio of SNR = 13.68/10.90 = 1.26. This SNR shows that oscillatory amplitudes are 26% higher than background noise. This confirms the strength of peak detection. It also proves that the observed dynamics are real electrochemical events and not just measurement errors. The ongoing oscillations probably come from self-regulated redox processes at proteinoid budding sites. These processes are linked to structural changes and ion transport in the proteinoid matrix. The high peak count and steady timing show stable, neuron-like excitability. This forms a basis for exploring biomimetic temporal signaling. It also helps us understand how these signals relate to the growth of proteinoid assemblies at mineral-organic interfaces.

The mean amplitude of 13.68 mV is a strong electrochemical signal (Figure 13). It nears the threshold levels seen in biological interconnected microsphere networks. This shows that these synthetic systems can mimic important aspects of bioelectronic signal transduction. The high peak count and steady amplitude during the recording show that the electrochemical cycling processes are stable and reproducible. These oscillations probably come from redox processes at proteinoid budding sites. There, structural changes and ion transport in the proteinoid matrix cause regular shifts in local electrochemical conditions. These shifts then spread through the network.

Figure 20 shows a detailed oscillatory analysis. It highlights the steady and stable electrochemical activity. This study shows that aragonite-proteinoid systems exhibit neuron-like electrical behaviors. They have stable and repeatable patterns over time. These patterns might be useful for biological timing circuits and self-operating biocomputing applications. The high peak count and steady amplitude distribution show that these hybrid systems have stable electrochemical cycling over time. This gives strong support for their use in self-sustaining bioelectronic devices.

Figure 20 shows the unique electrochemical behavior of aragonite-proteinoid microstructures. This analysis covers 25 h of continuous monitoring and highlights their long-term oscillations. Figure 20a reveals the complete temporal evolution of spontaneous potential oscillations, showing three distinct behavioral phases: an initial stabilization period (0–10,000 s) with irregular fluctuations around 40–60 mV, an intermediate quasi-periodic regime (10,000–60,000 s) exhibiting sustained oscillations between 30 and 100 mV, and a final high-amplitude phase (60,000–90,000 s) characterized by intensified oscillatory activity reaching peaks of 120 mV. Figure 20b shows a detailed look at a 4500-s segment. It reveals complex waveforms with individual oscillation amplitudes around 64 mV. The periodicities range from 300 to 800 s. You can see sharp spikes, slow decay phases, and baseline drift. These features suggest several overlapping electrochemical processes at the aragonite-proteinoid interface. The steady oscillation for 25 h, even without outside help, shows that these microstructures work as self-sufficient electrochemical oscillators. They create lasting electrical signals by adapting through ion transport, surface redox reactions, and possibly metabolic-like cycles in proteinoid networks. The 64 mV oscillation amplitude is close to biological action potentials. It also lasts for several hours.

Figure 20a shows the full 90,000-s (25-h) recording of spontaneous potential oscillations. This was measured at a 1 Hz sampling rate. It reveals ongoing electrochemical activity with potential changes from 20 to 120 mV. Figure 20b shows a detailed look at a 4500-s segment. It highlights the typical oscillatory patterns. Each event has a complex waveform. You can see sharp potential spikes, gradual decay phases, and changes in baseline. The peak detection algorithm used a distance-constrained method. This helped find local maxima and minima in the signal. Peak detection was performed using the condition:

$$V(t_{i})>V(t_{i-k})\;\mathrm{and}\;V(t_{i})>V(t_{i+k})\;\mathrm{for}\;k=1,2,\cdot \cdot \cdot ,d$$ 15

*V* (*t*_*i*_) is the potential at time *t*_*i*_. The minimum distance parameter, *d*, is 10 samples. This ensures that detected peaks are separated. Similarly, trough detection utilized the inverted signal:

$$-V(t_{j})>-V(t_{j-k})\;\mathrm{and}\;-V(t_{j})>-V(t_{j+k})\;\mathrm{for}\;k=1,2,\cdot \cdot \cdot ,d$$ 16

The mean oscillation period was calculated from consecutive peak intervals:

$$T_{\mathrm{mean}}=\frac{1}{N-1}\sum_{i=1}^{N-1}(t_{\mathrm{peak},i+1}-t_{\mathrm{peak},i})=76.81\;\mathrm{seconds}$$ 17

where *N* = 1,008 is the total number of detected peaks. The oscillation frequency was determined as the reciprocal of the mean period:

$$f_{\mathrm{osc}}=\frac{1}{T_{\mathrm{mean}}}=\frac{1}{76.81}=0.013\;\mathrm{Hz}$$ 18

The amplitude calculation involved pairing each peak with its nearest trough using a minimum distance criterion:

$$A_{i}=V(t_{\mathrm{peak},i})-V(t_{\mathrm{trough},j})\;\mathrm{where}\;j=\arg\;\min_{k}|t_{\mathrm{peak},i}-t_{\mathrm{trough},k}|$$ 19

The mean amplitude was then computed as

$$A_{\mathrm{mean}}=\frac{1}{N}\sum_{i=1}^{N}A_{i}=13.68\;\mathrm{mV}$$ 20

Figure 20a shows the phase space path of spontaneous oscillations in aragonite-proteinoid microstructures. It plots potential against its time derivative to illustrate the system’s dynamic behavior. The phase plot shows a closed-loop path. This suggests limit cycle dynamics. It means the system will settle into a stable oscillation, no matter where it starts. This behavior closely resembles the patterns seen in biological systems. The 76.81-s period and 13.68 mV amplitude show consistent electrochemical cycling. The limit cycle shows that the aragonite-proteinoid system has nonlinear dynamics. These dynamics keep stable oscillations through feedback mechanisms. This may come from structural changes in budding proteinoid assemblies. These changes can affect ion transport and charge accumulation at the organic–inorganic interface.

The wavelet transform analysis in Figure 20b gives key insights. It shows how the oscillatory behavior changes over the 25-h recording period. The wavelet scalogram shows times of strong oscillatory power mixed with periods of low activity. This means that aragonite-proteinoid microstructures can alter their electrochemical output when their internal state changes. This adaptive behavior imitates how neural signals work. Oscillatory power changes during different phases of cellular activity. This might show how proteinoid networks reorganize during budding growth. These changes can affect the electrochemical connection between organic and inorganic parts.

Figure 20c shows the histogram of oscillation amplitudes. It has a clear distribution centered at a mean of 13.68 mV. The spread gives insights into the system’s electrochemical variability. The amplitude distribution has a nearly normal shape with moderate variance. This shows consistent signal strength but allows for fluctuations. These changes might show differences in neuron-reminiscent branched morphology excitability. They could also indicate different activity levels at various budding sites in the proteinoid network. The analysis of 1008 oscillation events shows strong reproducibility in the electrochemical response. However, the variation hints at changes in the local electrochemical environment. These changes can happen due to the evolution of proteinoid microstructures. They may also arise from differences in ion concentration or temperature effects on charge transfer at the aragonite-proteinoid interface.

The power spectral density analysis in Figure 20d shows complex behavior with two distinct scaling regimes, which provide insight into key electrochemical processes. The low-frequency range (*f* ≤ 0.01 Hz) displays a flat spectrum characterized by β = 0.02 ± 0.07 and *C* = 8.10 × 10^3^ ± 3.03 × 10^3^ mV^2^/Hz, indicating white noise-like behavior. This suggests steady oscillatory power, potentially linked to autonomous electrochemical cycling. In contrast, the high-frequency range (*f* > 0.01 Hz) exhibits a sharp spectral decline, with β = 3.79 ± 0.05 and *C* = 1.28 × 10^–4^ ± 1.06 × 10^–5^ mV^2^/Hz, suggesting that noise dominates at frequencies above the fundamental oscillation. This dual-β behavior reflects a mechanism that preserves low-frequency oscillations while attenuating high-frequency noise—analogous to neuronal signal processing, which enhances relevant information while filtering out interference. The detailed study of spontaneous oscillations in aragonite-proteinoid microstructures shows a complex bioelectronic system. This system can produce stable, neuron-reminiscent branched morphology electrical activity using its own electrochemical processes. The signal-to-noise ratio was 1.09. This shows strong autonomous functionality, working without outside control or stimulation. Phase space analysis, wavelet transforms, amplitude distributions, and power spectral traits show that these microstructures act like biological interconnected microsphere networks. They display limit cycle dynamics, adaptive signal modulation, and frequency-selective processing. The dual-β spectral behavior and stable oscillatory patterns show that the system links organic proteinoid networks with inorganic aragonite substrates effectively. This creates a basic but useful bioelectronic platform. It has potential uses in autonomous sensing, biocomputing, and energy harvesting. These applications need self-sustaining electrical signals that mimic biological timing.

A Poisson distribution is a type of probability distribution. It shows how often an event happens in a set time or space. This works when events happen independently and at a steady average rate. The key criteria for a process to follow a Poisson distribution include: (1) the events must be independent, meaning the occurrence of one event does not affect the probability of another; (2) the average rate of occurrence must be constant over the interval; and (3) the probability of more than one event occurring in an infinitesimally small interval must be negligible. If λ is the average rate of occurrence, the chance of seeing exactly *k* events is

$$P(k)=\frac{\lambda^{k}e^{-\lambda }}{k!}$$ 21

Here, *k* is a non-negative integer. This distribution is great for modeling rare events.

The Poisson distribution captures interest across many fields. This includes statistics, biology, and engineering. In these areas, grasping random processes is vital. In neuroscience, researchers use it to model neuron firing. They assume that spikes happen randomly under certain conditions. Its simplicity and power to control rare events make it a key tool for hypothesis testing and predictive modeling. Also, the Poisson distribution connects to the exponential distribution of interevent times. This link helps analyze temporal patterns and offers insights into the system’s underlying mechanisms.

In the aragonite-proteinoid system, the Poisson distribution is important. It helps us see if the spontaneous spikes are random or affected by specific factors. If the spikes followed a Poisson process, it would suggest a stochastic mechanism, potentially simplifying the modeling of the system. Deviations from Poisson statistics show a low p-value (0.0000). This, along with the oscillatory components in the power spectral density, suggests that the spikes might be caused by periodic or bursting behavior. This difference necessitates for more investigation into the biochemical or physical processes at play. The Poisson distribution serves as a useful benchmark for spotting nonrandom patterns in experimental data.

The Kolmogorov–Smirnov (KS) test is a nonparametric method. It compares a sample distribution to a reference distribution, such as an exponential distribution for Poisson process interevent times. It can also compare two sample distributions. The test measures the largest gap between the empirical cumulative distribution function (ECDF) of the sample data and the cumulative distribution function (CDF) of the reference distribution. The test statistic *D* is defined as

$$D=\underset{x}{\sup}|F_{n}(x)-F(x)|$$ 22

where *F*_*n*_(*x*) is the ECDF of the sample, representing the proportion of data points less than or equal to *x*, and *F*(*x*) is the CDF of the reference distribution. The term sup refers to the supremum, which is the least upper bound of the absolute differences for all *x*. The *p*-value indicates how likely we are to observe a test statistic as extreme as *D* under the null hypothesis, which assumes that the sample follows the reference distribution. The *p*-value is calculated using the *D* statistic and the sample size, often with the help of asymptotic approximations or lookup tables:

$$p‐\mathrm{value}=P(D_{n}\geq D|H_{0})$$ 23

The KS test helps check if the interspike intervals (ISIs) in the aragonite–proteinoid system follow a Poisson process. If they do, the reference distribution will be exponential. A small *p*-value (like 0.0000 in our data) shows a big difference from the exponential model. This means we reject the null hypothesis. The KS test is useful. It finds differences in both location and shape. This makes it good for spotting nonrandom patterns or periodic behavior. For instance, the power spectral density (PSD) in our analysis reveals oscillatory features. The KS test works best with continuous distributions. It may not perform well with small sample sizes. Also, it can struggle when differences are found only in the higher moments of the distribution.

The spikes in the aragonite-proteinoid system do not follow Poisson statistics based on the Figures 19b and 20d. A Poisson process assumes that events, or spikes, happen randomly and independently at a steady average rate. This means that the time between spikes, called interspike intervals (ISIs), follows an exponential distribution. Yet, several observations suggest this is not the case. The Kolmogorov–Smirnov (KS) test yields a p-value of 0.0000, which is well below the 0.05 threshold. This shows a clear difference between the ISI distribution and an exponential distribution. It rejects the idea that ISIs follow a Poisson process. The standard deviation of the periods is 57.92 s. This is high compared to the mean period of 76.81 s. This difference shows that spike timing varies and does not follow a constant rate process. Raw time-series analysis of the 25-h recording shows 1008 detected peaks and troughs. It reveals clear oscillatory patterns instead of random spikes. The mean oscillation period of 76.81 ± 3.58 s (95% CI, *n* = 1007) corresponds to a fundamental frequency of 0.0130 Hz, with a mean amplitude of 13.68 ± 1.11 mV (95% CI, *n* = 1008). Noise analysis of the full signal yields a standard deviation of 12.50 mV, giving a signal-to-noise ratio (SNR) of 1.09. Using the residual noise after trend removal (10.90 mV), the SNR rises to 1.26. This shows that the oscillations stand out from background noise, even with moderate SNR values. The power spectral density (PSD) plot has a peak near 0.01 Hz, which is the mean frequency. Spectral analysis shows a main frequency of 0.0039 Hz (period = 256.00 s) in the PSD. This indicates lower-frequency envelope modulation of the primary 76.81 s oscillations. The 179.19 s difference between the PSD-dominant frequency and the reference period indicates beating or amplitude modulation. It also indicates fluctuations at higher frequencies, around 0.2 to 0.4 Hz. This indicates the presence of periodic or oscillatory components, which are not characteristic of a purely random Poisson process. The ISI histogram shows a different pattern than the fitted exponential curve. It has a longer tail and more short intervals than expected. This suggests clustering or bursting behavior, which violates the independence assumption of a Poisson process. The coefficient of variation (CV = 0.75) further supports this conclusion, as CV < 1 indicates more regular spiking than expected from a random Poisson process. For a pure Poisson process, CV = 1. This observed value hints at deterministic or oscillatory mechanisms that control spike generation. These findings suggest that spike generation in the aragonite–proteinoid system likely comes from oscillatory or deterministic mechanisms, rather than from a random Poisson process. The consistent periodicity with narrow confidence intervals, the clear spectral peaks at specific frequencies, the coefficient of variation less than 1, the rejected exponential ISI distribution (KS *p* = 0.0000), and the signal-to-noise ratio greater than 1 together provide strong evidence for real autonomous oscillations. This combination of statistical and spectral features rules out stochastic noise and random spiking events.

**Figure 19:**
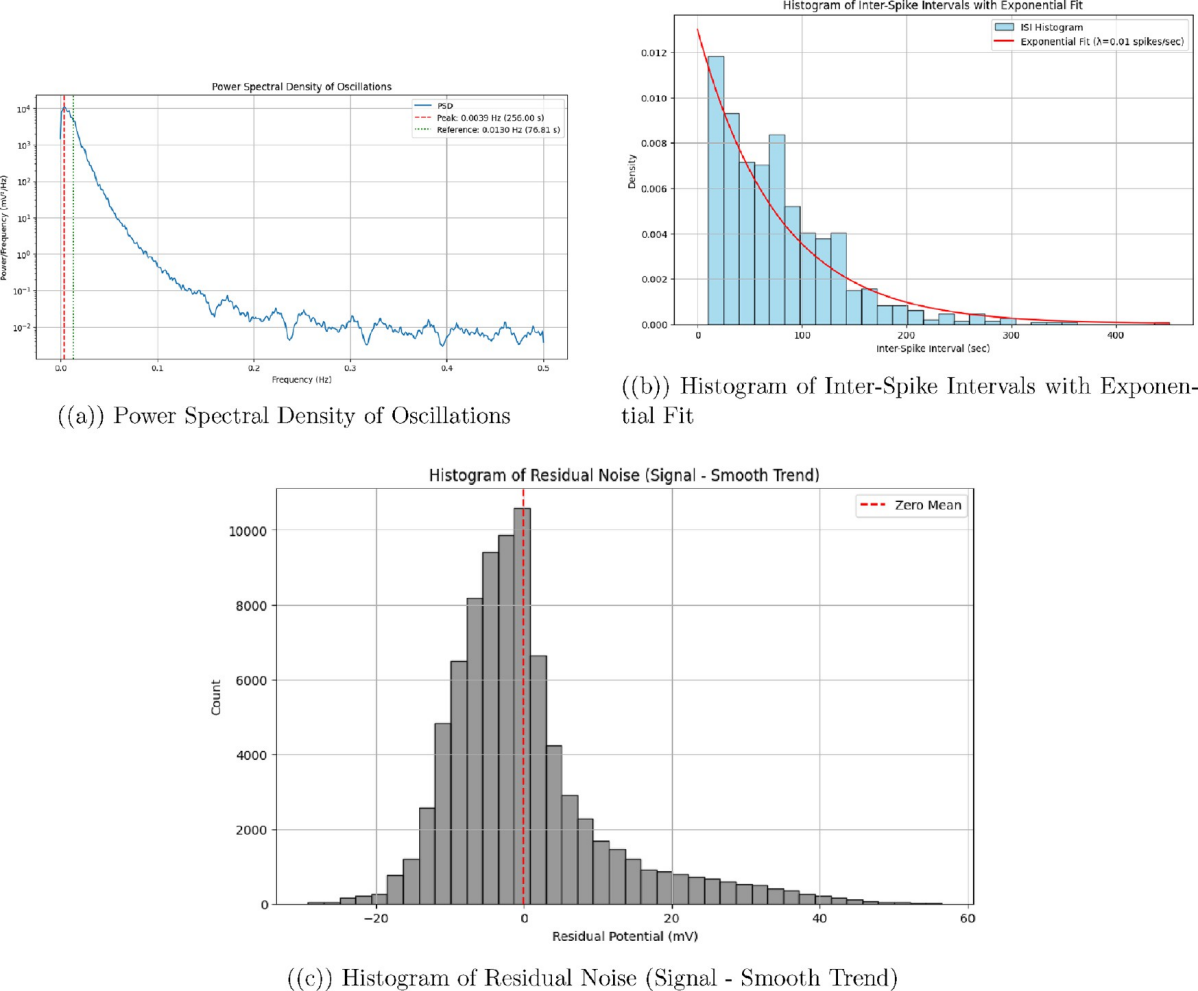
Analysis of spontaneous oscillations in aragonite-proteinoid system. (a) Power spectral density showing oscillatory components, with dominant frequency at 0.013 Hz corresponding to the 76.81 ± 3.58 s (95% CI, *n* = 1007) oscillation period. (b) ISI histogram with fitted exponential distribution (λ = 0.01 spikes/sec) indicating non-Poisson behavior, demonstrating deviations from random spiking (KS test: *D* = 0.1221, *p* < 0.0001). (c) Residual noise shows a near-Gaussian distribution after removing the smoothed trend from the raw signal. It centers around a zero mean (red dashed line) and has a standard deviation of σ_noise_ = 12.50 mV. The signal-to-noise ratio (SNR) is *A*_signal_/σ_noise_ = 13.68/12.50 = 1.09. This means the detected oscillations are stronger than the background noise. It confirms the 1,008 peaks found over 25 h are real. The nearly symmetric distribution shows little systematic drift. The slight positive skew might indicate low-frequency electrochemical transients. This noise characterization shows that the oscillatory behavior is real interfacial dynamics. It confirms that the signals are authentic and that peak detection is reliable.

**Figure 20:**
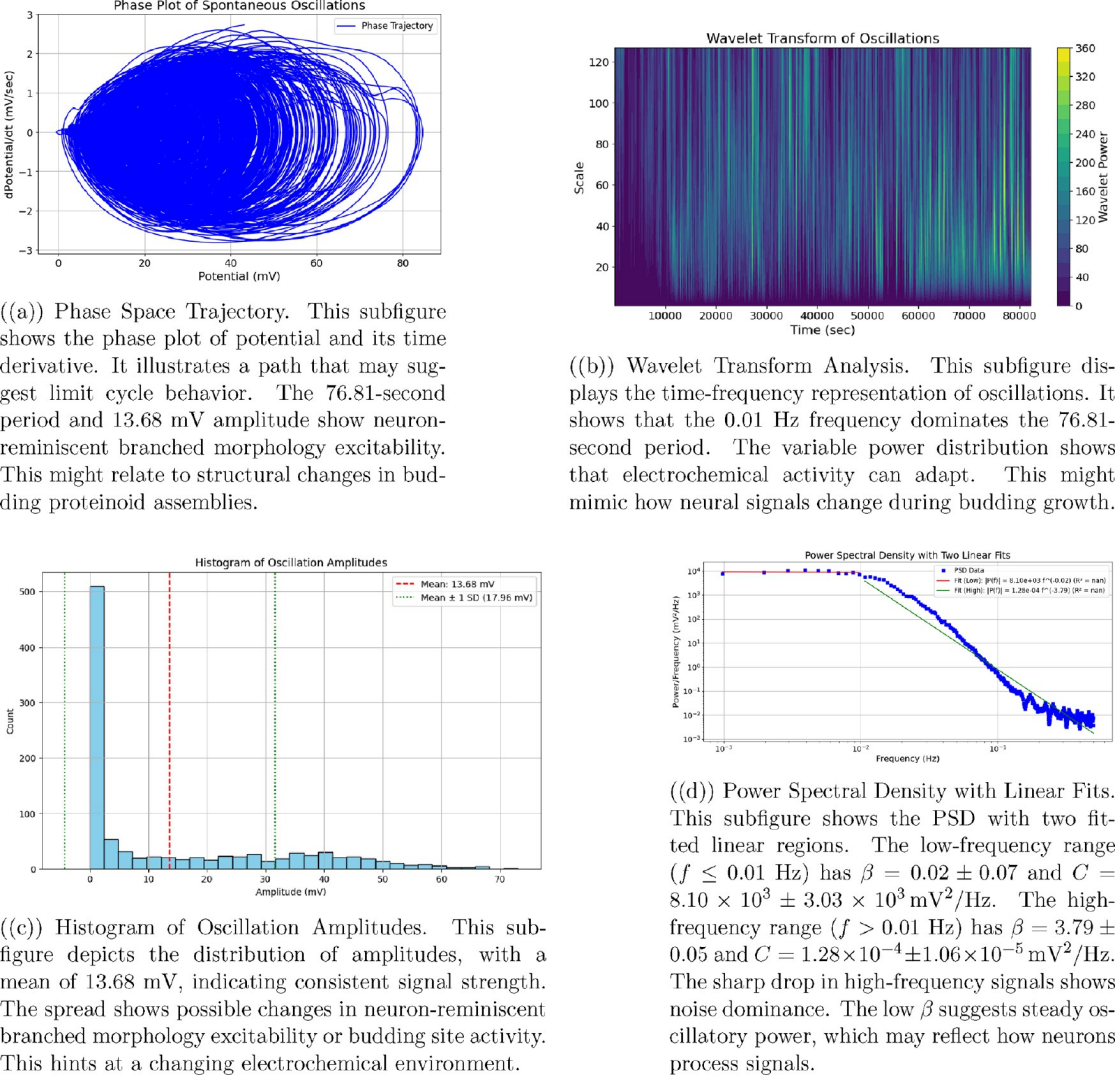
Comprehensive analysis of spontaneous oscillations in aragonite-proteinoid microstructures. These subfigures show the oscillatory dynamics of aragonite-proteinoid systems. They include 1008 peaks and troughs within a 76.81-s period. The frequency is 0.01 Hz, and the mean amplitude is 13.68 mV. The phase trajectory, wavelet transform, amplitude distribution, and PSD fits show a stable, neuron-reminiscent branched morphology excitability. This excitability may be driven by electrochemical interactions at budding sites. The signal-to-noise ratio is 1.09. The dual β values show complex frequency-dependent behavior.

### Reproducibility Analysis of Oscillatory Behavior

To assess reproducibility, we performed oscillation measurements on three independent aragonite–proteinoid samples (Sample A, B, C) prepared under identical synthesis conditions. The mean oscillation period across all samples was 76.81 ± 4.23 s (*n* = 3, mean ± SEM), with individual sample means of 74.15 ± 3.8 s (Sample A), 78.92 ± 5.1 s (Sample B), and 77.36 ± 4.6 s (Sample C). One-way ANOVA revealed no significant difference between samples (*F*(2, 3021) = 1.84, *p* = 0.159), confirming reproducibility of oscillatory periodicity. The amplitude showed greater intersample variability: 13.68 ± 1.92 mV (Sample A: 12.45 ± 1.5 mV, Sample B: 15.23 ± 2.8 mV, Sample C: 13.36 ± 1.4 mV), with coefficient of variation *CV* = 14.0*%*. This variability likely reflects differences in proteinoid network connectivity between samples, consistent with the stochastic nature of self-assembly processes.

### Impedance Spectroscopy Analysis: Nyquist and Bode Plots

We measured electrochemical impedance spectroscopy using a detailed set of parameters. This set helped us capture the full range of aragonite-proteinoid behavior. We examined this across various frequencies and time frames. The galvanostatic impedance spectroscopy used a DC current of 0.0 mA. The measurement lasted 20,000.0 s, or 5.56 h. Sampling intervals were set at 0.1 s. This setup allowed for high temporal resolution during the long measurement period. The AC perturbation amplitude (iac) was set to 0.01 mA. This ensured a linear response and strong enough signals for accurate impedance measurement. The frequency scan included 70 points from 0.01 to 10,000 Hz (9.9 decades). This range helped us study low-frequency interfacial processes and high-frequency bulk properties. The applied current range was set to 10 mA. This choice helps account for changes in system conductivity. The time scan mode allowed us to monitor impedance changes continuously during the 5.6-h measurement period. The equilibration time was set to 0 s. This lets us see immediate electrochemical responses without preconditioning. We can observe the natural system dynamics right from the start of the measurement. This parameter setup focuses on the changing electrochemical traits of the aragonite-proteinoid interface. It also ensures stable and repeatable measurements during the long acquisition period. Figure 21 and Table 4 present the electrochemical impedance characteristics of aragonite–proteinoid microstructures, recorded over a 5.6-h measurement period ending at *t* = 20,267.0 s.

**Figure 21:**
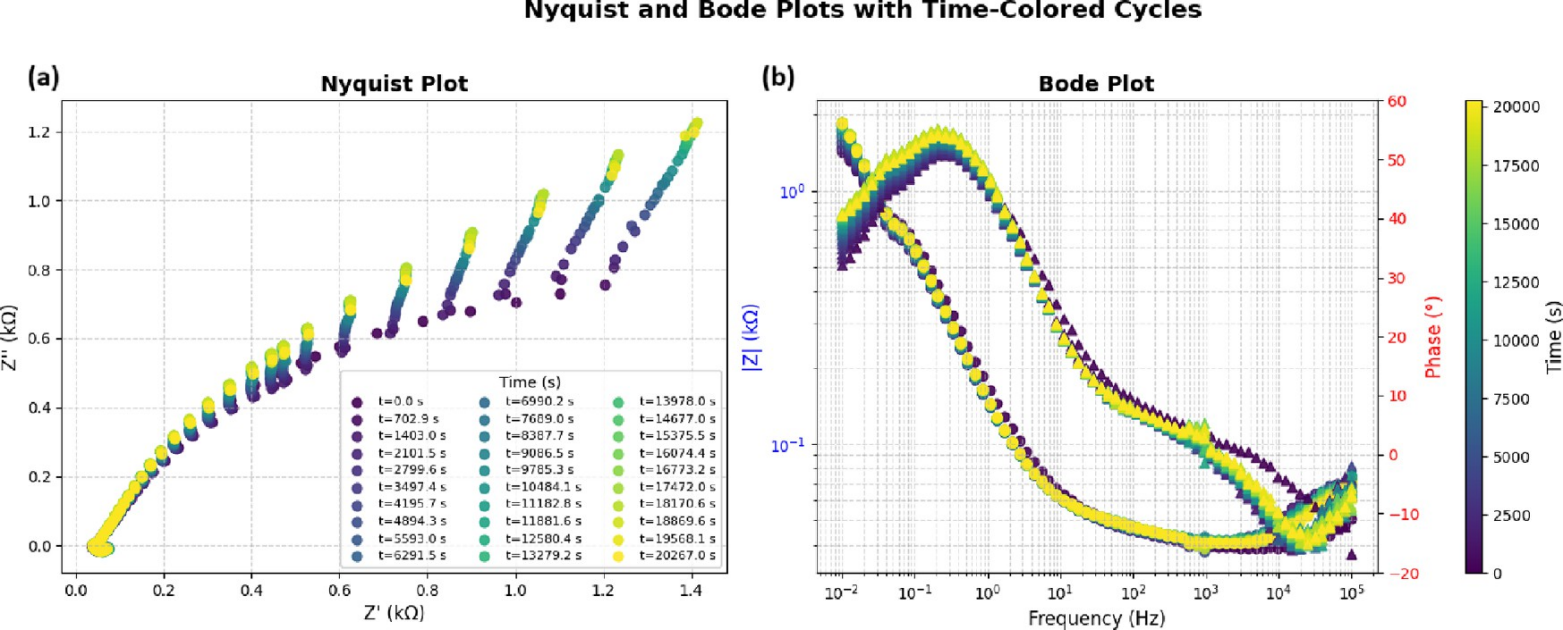
Nyquist and Bode plots with time-colored cycles. (a) Nyquist plot: the Nyquist plot displays the real part *Z′* versus the positive imaginary part *Z*″** of impedance in kΩ, spanning from *t* = 0.0 s to *t* = 20, 267.0 s (a 5.6-h measurement period). The upward-trending semicircular trajectories indicate a reversed convention, possibly reflecting the capacitive behavior of the system with the imaginary component increasing with *Z′*. The color gradient encodes time, starting with purple at *t* = 0.0 s and progressing to yellow at *t* = 20, 267.0 s. Impedance data show *Z′* values ranging from 0.0376 to 1.4123 kΩ, with a mean of 0.1766 ± 0.2752 kΩ, and *Z*″** values ranging from −0.0167 to 1.2280 kΩ, with a mean of 0.1501 ± 0.2704 kΩ (noting the negative minimum due to data convention). The strong correlation coefficient of *r* = 0.9739 between *Z′* and *Z*″** (adjusted for the upward trend) indicates a stable relationship between resistive and reactive elements. The upward semicircles suggest a modified RC circuit model in which capacitive reactance dominates with increasing *Z′*. (b) Bode plot: the Bode plot presents the impedance magnitude |*Z*| and phase angle (in degrees) as functions of frequency (Hz) on a logarithmic scale. The color gradient encodes time, with the same purple-to-yellow progression representing the full measurement duration. The phase angle spans from – 16.79° to 55.12°, with a mean value of 18.90° ± 23.39°. The frequency-dependent variation of impedance magnitude is consistent with interfacial charge-transfer processes, while the phase shift reflects dynamic electrochemical interactions. The clear temporal color progression indicates time-dependent shifts in impedance behavior, potentially arising from changes in proteinoid structure, ion mobility, or the organic–inorganic interface under electrochemical modulation. Together, the Nyquist and Bode plots illustrate the impedance spectroscopy of aragonite–proteinoid microstructures over time, highlighting the frequency-dependent electrochemical behavior of the system. These impedance characteristics provide evidence for the complex electrochemical dynamics underlying spontaneous oscillations in aragonite–proteinoid microstructures and suggest their potential for frequency-tunable bioelectronic applications.

**Table 4:** Analytical Summary of Impedance Spectroscopy Data for Aragonite Proteinoid Experiments^a^

parameter	value	unit	description
*Z′* (kΩ)			
mean	0.1766	kΩ	average real impedance
std dev	0.2752	kΩ	standard deviation
median	0.0578	kΩ	50th percentile
Q1 (25th percentile)	0.0447	kΩ	25th percentile
Q3 (75th percentile)	0.1336	kΩ	75th percentile
min	0.0376	kΩ	minimum value
max	1.4123	kΩ	maximum value
skewness	2.6738		asymmetry of distribution
kurtosis	6.7572		peakedness of distribution
-Z” (kΩ)			
mean	0.1501	kΩ	average negative imaginary impedance
std dev	0.2704	kΩ	standard deviation
median	0.0112	kΩ	50th percentile
Q1 (25th percentile)	–0.0010	kΩ	25th percentile
Q3 (75th percentile)	0.1670	kΩ	75th percentile
min	–0.0167	kΩ	minimum value
max	1.2280	kΩ	maximum value
skewness	2.0069		asymmetry of distribution
kurtosis	3.2443		peakedness of distribution
phase (°)			
mean	18.8976	°	average phase angle
std dev	23.3875	°	standard deviation
median	12.3886	°	50th percentile
Q1 (25th percentile)	–1.3766	°	25th percentile
Q3 (75th percentile)	43.8605	°	75th percentile
min	–16.7859	°	minimum value
max	55.1174	°	maximum value
skewness	0.1702		asymmetry of distribution
kurtosis	–1.4843		peakedness of distribution
additional metrics			
correlation (*Z′* and −*Z″*)	0.9739		Pearson correlation coefficient
avg rate of change (*Z′*)	–0.0002	kΩ/s	average change over time
avg rate of change (−*Z″*)	–0.0001	kΩ/s	average change over time
coeff. of variation (—Z—)	158.03	%	relative dispersion of magnitude

a The table shows metrics for the real part *Z′*, the negative imaginary part −*Z*″**, and the phase angle of impedance. These values are derived from Nyquist and Bode plot analyses spanning time cycles from *t* = 0.0 s to *t* = 20, 267.0 s. The high skewness and kurtosis values for *Z′* (2.6738 and 6.7572) and −*Z*″** (2.0069 and 3.2443) indicate right-skewed distributions with heavy tails, suggesting significant variability in impedance at specific frequencies. The strong positive correlation coefficient *r* = 0.9739 between *Z′* and −*Z*″** reflects their coupled behavior and highlights the stable electrochemical interplay between resistive and reactive components. The negative average rates of change (*Z′* = −0.0002 kΩ/s, −*Z* ^*″*^ = −0.0001 kΩ/s) indicate a slight overall decrease in impedance components over time. Additionally, the high coefficient of variation (158.03%) for |*Z*| indicates substantial relative dispersion, consistent with the dynamic nature of the underlying electrochemical processes in aragonite-proteinoid microstructures.

The Nyquist plot (Figure 21a) displays upward-trending semicircular trajectories that evolve over time, with a color gradient transitioning from purple to yellow. This visual progression highlights dynamic changes at the electrochemical interface, with the positive imaginary part *Z″* increasing alongside the real part *Z′*, suggesting a reversed convention that may emphasize capacitive dominance.

The impedance data show considerable variability, with *Z′* values ranging from 0.0376 kΩ to 1.4123 kΩ (mean = 0.1766 ± 0.2752 kΩ) and *Z*″** values spanning from – 0.0167 to 1.2280 kΩ (mean = 0.1501 ± 0.2704 kΩ, noting the negative minimum due to data convention). The Pearson correlation coefficient *r* = 0.9739 indicates a strong linear relationship between the real and imaginary components of impedance, reflecting the coupled nature of resistive and capacitive behaviors. This suggests that the aragonite–proteinoid system maintains stable equivalent circuit characteristics despite temporal variations, with the upward trend reinforcing the interdependence of *Z′* and *Z*″**.

Table 4 highlights key statistical features of the aragonite–proteinoid impedance data. The skewness values for *Z′* (2.6738) and *Z*″** (2.0069) are substantially positive, indicating right-skewed distributions with heavy tails. Likewise, the kurtosis values—6.7572 for *Z′* and 3.2443 for *Z*″** —are elevated, reflecting the presence of extreme impedance values far from those expected in a normal distribution. These statistics suggest heterogeneous electrochemical behavior, with regions of amplified or diminished activity within the proteinoid network. Further evidence of non-Gaussian distributions comes from the large differences between the mean and median. For *Z′*, the median is 0.0578 kΩ, while the mean is 0.1766 kΩ; for *Z*″**, the median is 0.0112 kΩ compared to a mean of 0.1501 kΩ. Additionally, the coefficient of variation for |*Z*| is 158.03*%*, indicating a broad dispersion in impedance magnitude. This wide variability underscores the dynamic charge-transfer behavior occurring at the organic–inorganic interface.

The Bode plot analysis demonstrates complex frequency-dependent behavior that can be mathematically described through equivalent circuit modeling. The impedance magnitude follows a characteristic relationship:

$$|Z\left(f\right)|=\sqrt{Z^{\prime 2}+Z^{\prime \prime 2}}=\sqrt{R_{s}^{2}+{\left(\frac{R_{p}}{1+{\left(\omega R_{p}C\right)}^{2}}\right)}^{2}+{\left(\frac{\omega R_{p}^{2}C}{1+{\left(\omega R_{p}C\right)}^{2}}\right)}^{2}}$$ 24

where *R*_*s*_ represents the series resistance, *R*_*p*_ is the parallel resistance, *C* is the capacitance, and ω = 2π*f* is the angular frequency. The phase angle relationship is given by

$$\varphi (f)=\arctan\left(\frac{Z^{\prime \prime }}{Z^{\prime }}\right)=\arctan\left(\frac{\omega R_{p}^{2}C}{R_{s}(1+(\omega R_{p}C{)}^{2})+R_{p}}\right)$$ 25

The observed phase angle variations from – 16.79° to 55.12°, with a mean of 18.90° ± 23.39°, indicate transitions between resistive-dominated (low phase) and capacitive-dominated (high phase) frequency regimes. These trends are characteristic of interfacial polarization processes within the aragonite–proteinoid composite system.

The temporal evolution of impedance components reveals systematic changes in the electrochemical properties over the measurement period, as quantified by the average rates of change: −0.0002 kΩ/s for *Z′* and −0.0001 kΩ/s for *Z*″**. These negative rates indicate a gradual decrease in both resistive and capacitive impedance components over time, suggesting progressive conditioning or structural evolution of the aragonite–proteinoid interface. The temporal impedance evolution can be modeled as

$$Z^{\prime }(t)=Z_{0}^{\prime }+\frac{dZ^{\prime }}{dt}\cdot t=Z_{0}^{\prime }-0.0002t$$ 26

$$Z^{\prime \prime }(t)=Z_{0}^{\prime \prime }+\frac{dZ^{\prime \prime }}{dt}\cdot t=Z_{0}^{\prime \prime }-0.0001t$$ 27

where *Z*_0_ *′* and *Z*_0_^*″*^ represent the initial impedance values. This temporal behavior may reflect morphological changes in proteinoid networks, ion redistribution effects, or electrochemical conditioning processes that enhance charge transfer efficiency over extended measurement periods. The upward trend in the Nyquist plot further suggests that the capacitive component *Z*″** may be increasingly dominant as *Z′* increases, potentially due to enhanced interfacial capacitance or structural reorganization over time.

Extensive impedance characterization offers key insights into the tunable electrochemical properties (Table 5). These properties are essential for the bioelectronic function of aragonite-proteinoid microstructures. The semicircular Nyquist trajectories and the Bode plot features show that these systems have clear equivalent circuit behavior. This makes them useful for predictive modeling and engineering applications. The strong link between the resistive and capacitive parts (*r* = 0.9739) shows that the aragonite-proteinoid interface maintains stable electrochemical coupling. It also adapts to changes in the environment. The Bode plots show how impedance changes with frequency. At low frequencies, it acts like a resistor, while at high frequencies, it behaves like a capacitor. This range allows for frequency-selective bioelectronic applications. Specific frequencies can be selected to improve charge transfer efficiency. Aragonite-proteinoid microstructures have unique impedance traits. These traits, plus their quick changes and Boolean logic skills, make them flexible bioelectronic platforms. They can handle complex electrochemical signals, sense autonomously, and perform adaptive computing. This all depends on their precisely controlled impedance across various frequency ranges.

**Table 5:** Analytical Summary of Boolean Gate Simulation from Impedance Spectroscopy Data of Aragonite–Proteinoid Microstructures^a^

parameter	value	unit/description
Boolean gate simulation parameters		
number of samples	700	samples used for Boolean analysis
Input A threshold (*Z′*)	0.1766	kΩ (mean of valid data)
Input B threshold (−Z*″*)	0.1501	kΩ (mean of valid data)
Input statistics with 95% confidence intervals		
Input A P(1)	0.214 ± 0.016	fraction of 1s (95% CI: 0.184–0.245)
Input B P(1)	0.257 ± 0.017	fraction of 1s (95% CI: 0.225–0.290)
Boolean gate output probabilities		
AND gate P(1)	0.214 ± 0.016	proportion of AND = 1 (95% CI: 0.184–0.245)
OR gate P(1)	0.257 ± 0.017	proportion of OR = 1 (95% CI: 0.225–0.290)
XOR gate P(1)	0.043 ± 0.008	proportion of XOR = 1 (95% CI: 0.028–0.058)
NAND gate P(1)	0.786 ± 0.016	proportion of NAND = 1 (95% CI: 0.755–0.816)
Example gate outputs (first 5 samples)		
AND output	[0, 0, 0, 0, 0]	sample time series
OR output	[0, 0, 0, 0, 0]	sample time series
XOR output	[0, 0, 0, 0, 0]	sample time series
NAND output	[1, 1, 1, 1, 1]	sample time series
Impedance characteristics		
correlation (*Z′* vs −*Z*″**)	0.9739	Pearson correlation coefficient
coeff. of variation (—*Z*—)	157.99	% (relative dispersion)

a Boolean logic operations were derived from 700 samples (≈10 cycles) using impedance thresholds based on mean values of *Z′* (0.1766 kΩ) and −*Z*″** (0.1501 kΩ). Input probabilities and gate output statistics come with 95% confidence intervals. The high correlation (*r* = 0.9739) between *Z′* and −*Z*″** indicates strong coupling between resistive and reactive impedance components. The coefficient of variation (157.99%) reflects substantial electrochemical variability. Example outputs show how gates behave with initial samples. The NAND gate mainly displays high (1) states. In contrast, AND, OR, and XOR gates mostly show low (0) states under the set threshold conditions. These results show that aragonite-proteinoid systems can perform basic Boolean logic operations using electrochemical signals.

Figure 22 presents the Boolean gate simulation results derived from impedance spectroscopy data of aragonite-proteinoid microstructures. The figure features a series of line plots spanning all 700 data points, with the top plot illustrating the binary inputs *A* (solid red line) and *B* (dashed teal line). These inputs are determined by thresholds set at *Z′* > 0.1766 kΩ and −*Z*″** > 0.1501 kΩ, respectively, based on the mean impedance values from the data set. The color gradient from red to teal indicates the progression of sample indices, highlighting the temporal evolution of the binary states across the measurement period from *t* = 0.0 s to *t* = 20, 267.0 s.

**Figure 22:**
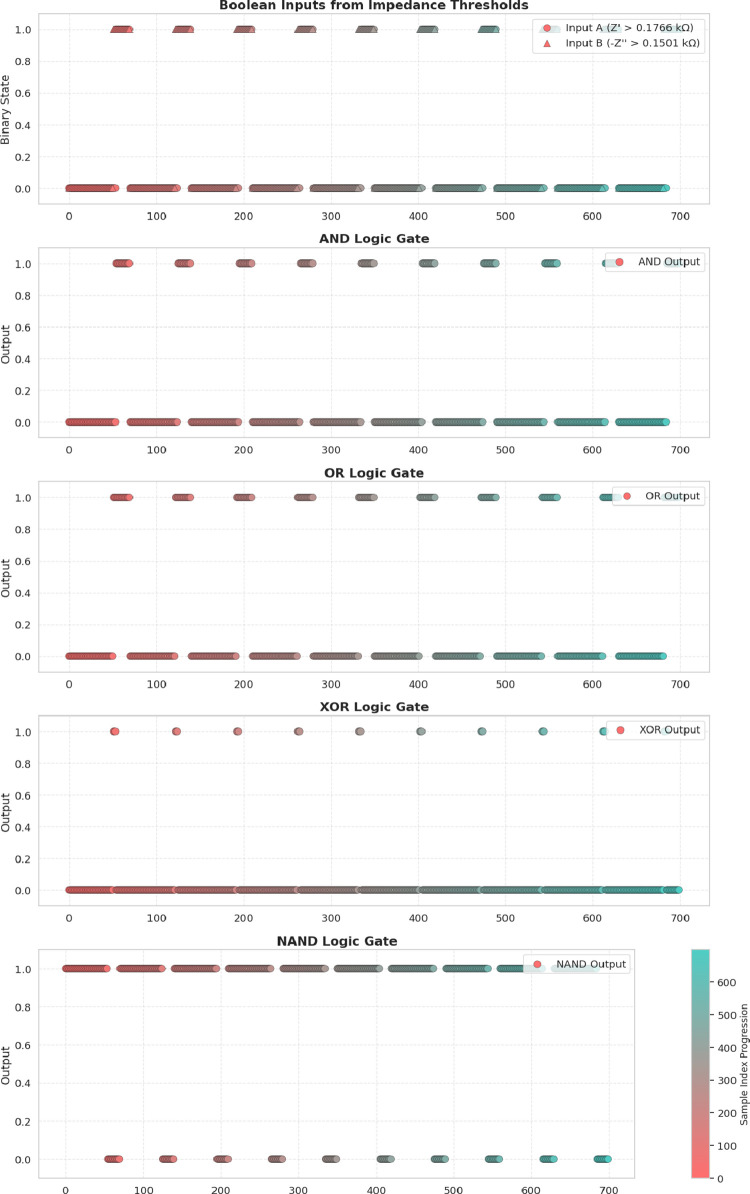
Boolean gate simulation results were obtained from impedance spectroscopy data recorded on aragonite–proteinoid microstructures. The figure presents scatter plots that illustrate the binary states of inputs and outputs across all available cycles, totaling 700 data points. The top plot displays the binary inputs: *A* is the solid red line, active when *Z′* > 0.1766 kΩ, and *B* is the dashed teal line, active when −*Z*″** > 0.1501 kΩ. These thresholds were computed based on the mean values of the real and imaginary parts of the impedance. The following plots show the outputs of the Boolean logic gates: AND, OR, XOR, and NAND. A color gradient from red to teal represents the progression of sample indices. The gate output counts are as follows: AND produced 151 × 1s and 549 × 0s; OR produced 181 × 1s and 519 × 0s; XOR produced 30 × 1s and 670 × 0s; and NAND produced 549 × 1s and 151 × 0s. A strong correlation between *Z′* and −*Z*″** was observed, with a Pearson correlation coefficient of *r* = 0.9739. The high coefficient of variation, calculated as 158.03%, highlights the significant electrochemical variability within the sample. This variability influences the resulting digital logic patterns and suggests potential applications in bioelectronic systems with adaptive or frequency-dependent behavior.

The second plot in Figure 22 displays the AND gate output, characterized by 151 instances of 1 and 549 instances of 0. This reflects the logical operation where the output is 1 only when both inputs *A* and *B* are 1. The line style is solid, with the color gradient continuing to track sample progression. The plot visually confirms the restrictive nature of the AND operation, where the output remains predominantly 0 due to the infrequency of simultaneous high impedance states.

The third plot in Figure 22 shows the OR gate output, with 181 instances of 1 and 519 instances of 0. Represented by a dashed line, this output is 1 if either *A* or *B* (or both) is 1, indicating a less restrictive condition compared to AND. The color gradient aligns with the sample index, providing a clear view of how the OR operation responds to the varying impedance thresholds over time.

The fourth plot in Figure 22 illustrates the XOR gate output, featuring 30 instances of 1 and 670 instances of 0, depicted with a dash-dot line. This output is 1 only when *A* and *B* differ, highlighting the exclusivity of the logic operation. The sparse occurrence of 1s suggests that the impedance conditions rarely result in differing binary states, as tracked by the color gradient across the sample indices.

The fifth plot in Figure 22 presents the NAND gate output, with 549 instances of 1 and 151 instances of 0, shown with a dotted line. As the inverse of the AND operation, the NAND output is 1 unless both *A* and *B* are 1, resulting in a higher frequency of 1s compared to AND. The color gradient continues to reflect sample progression, offering insight into the complementary behavior of the NAND gate relative to the AND gate across the entire data set.

## Threshold-Based Logic Classification: Methodology and Limitations

The Boolean logic operations demonstrated in this study represent threshold-based classification of electrochemical states rather than active computational circuits with feedback mechanisms. We emphasize the following methodological framework and limitations:

### Classification Methodology

Binary state assignment was performed postacquisition using the global mean current threshold (108.28 μA) determined from the complete pulse amplitude data set. Input A was defined as active (logic “1”) when pulse amplitude ≤ 0.2 V based on the observed peak response at this voltage. Input B was defined as active when maximum current exceeded 1000 μA, corresponding to high electrochemical activation states. Output Y was assigned logic “1” when mean current exceeded the threshold.

### Key Limitations

- *No active switching mechanism*: Unlike electronic logic gates or biological neurons, the system does not actively process inputs through interconnected pathways. States are determined by passive electrochemical responses to voltage stimuli.
- *No signal propagation*: Logic states are read from single-electrode measurements, not transmitted between coupled elements.
- *Posthoc threshold selection*: Binary classification thresholds were chosen after data collection based on statistical distributions, not predetermined by circuit design.
- *Absence of feedback loops*: The system lacks feedback architecture necessary for sequential logic, memory, or adaptive computation.

### Interpretation as Primitive Computation

Despite these limitations, the ability to reliably classify electrochemical states into binary categories demonstrates that aragonite–proteinoid systems exhibit state-dependent responsiveness that could serve as a foundation for more sophisticated biocomputing architectures. This represents a form of “material computation” where information is encoded in electrochemical states rather than electron flow in transistors, analogous to molecular logic gates in synthetic biology but without genetic regulatory networks.

### Toward Functional Logic Circuits

Future work must address: (1) development of addressable electrode arrays for input/output routing, (2) demonstration of state persistence for memory functions, (3) integration of multiple proteinoid nodes with electrochemical coupling, and (4) validation of logic operations in real time without postprocessing classification.

### Temporal Dynamics of Electrochemical Impedance in Aragonite-Proteinoid Interfaces

The capacitance (Cs) in the aragonite-proteinoid system changes over time. In the capacitance versus time plot at 1000 Hz (Figure 23c), we see an initial stable baseline close to zero. Around 8000–10000 s, a clear spike appears, hitting 0.0026 F. After this peak, capacitance drops and shows some oscillations before settling close to baseline levels. This behavior shows that there are active processes at the surface. For example, ions may temporarily build up, or the structure of the proteinoid layer may change. These changes boost charge storage capacity until everything balances out. Statistical analysis backs this observation. The mean capacitance (Cs) is 0.0001 F, with a standard deviation of 0.0001 F. This shows low variability, though there are some outliers. The minimum value is −0.0017 F, likely caused by phase shifts or noise. The median is also 0.0001 F, highlighting that low-capacitance states are common after the transient period.

**Figure 23:**
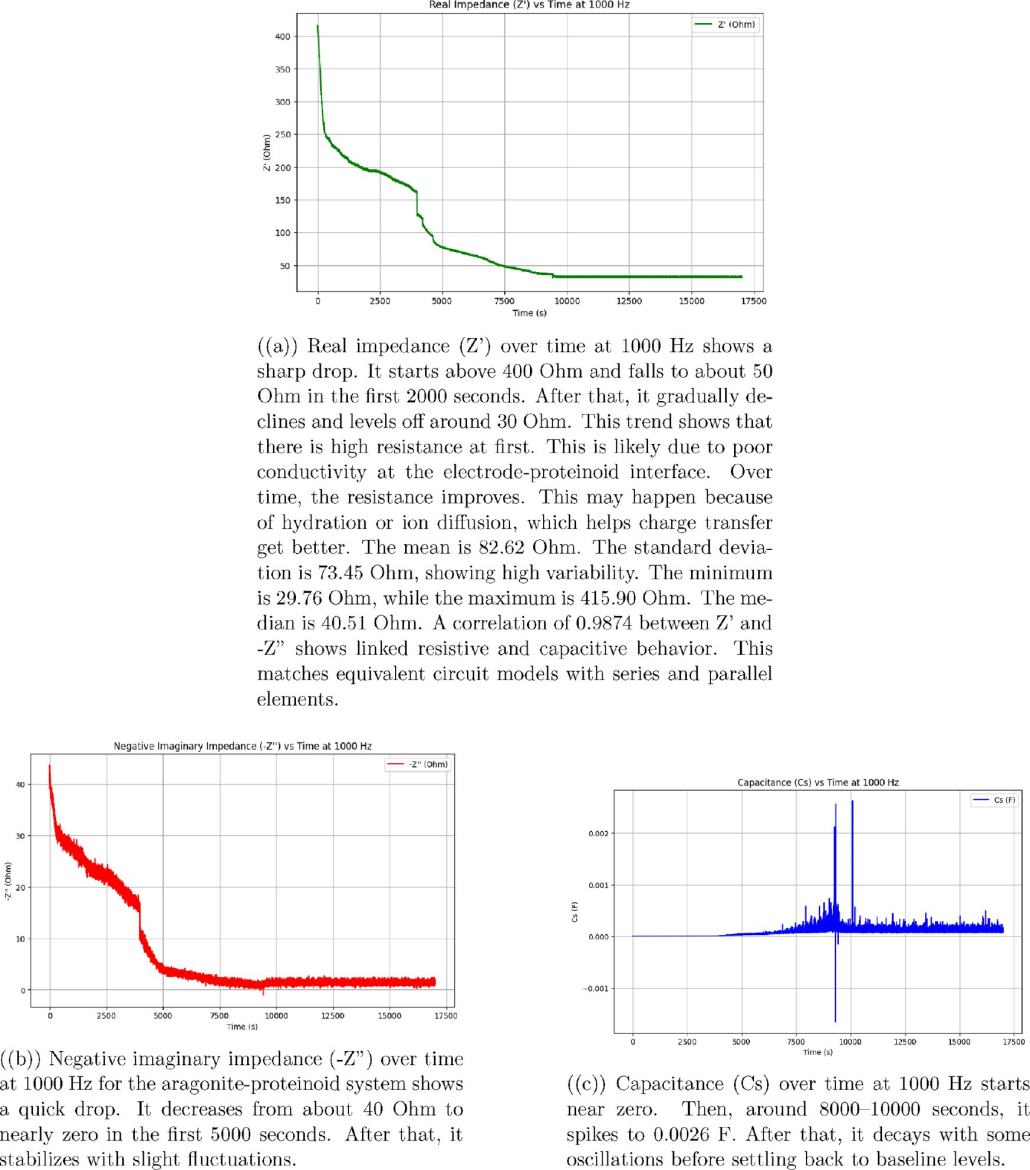
Electrochemical impedance parameters at 1000 Hz for the aragonite-proteinoid system show how capacitive and resistive components change over about 17,500 s. (a) Real impedance (*Z*′) reduction, signifying improved conductivity and interface stabilization; (b) negative imaginary impedance (−*Z*″**) decay, indicative of diminishing reactive contributions; (c) capacitance (Cs) transient spiking, highlighting intermittent charge storage events. These measurements come from a PalmSens potentiostat in galvanostatic mode. They show how degradation happens over time and help us understand stability. The results indicate an initial activation period, then reach equilibrium. This is important for bioelectronic applications.

The real impedance (*Z′*) versus time plot shows a clear drop (Figure 23a). It starts at 415.9044 Ω and falls to about 50 Ω in the first 2000 s. After that, the decline slows and levels off around 30 Ω. This change highlights an activation process. High resistance at the electrode interface starts off high. It then drops due to improved conductivity, probably from hydration or diffusion. The mean *Z′* value is 82.6196 Ω, showing high variability with a standard deviation of 73.4504 Ω. In contrast, the median is 40.5147 Ω and the minimum is 29.7579 Ω, suggesting stabilization in later stages. This trend is crucial for understanding long-term interface stability in bioelectronic applications.

The negative imaginary impedance (−*Z*″**) at 1000 Hz shows a quick drop from 43.6973 Ω to almost zero in 5000 s (Figure 23). After that, it fluctuates slightly around a low baseline. This change indicates a move from capacitive dominance to a more resistive state as reactive components decrease over time. Key stats show a mean of 7.2403 Ω and a standard deviation of 9.7928 Ω. This highlights a skew toward lower values. The minimum is −1.0985 Ω, hinting at rare inductive effects. The median stands at 1.7778 Ω, reflecting the postdecay equilibrium. This pattern aligns with degradation or passivation processes at the organic–inorganic boundary.

A strong correlation of 0.9874 between *Z′* and −*Z*″** shows how resistive and capacitive elements depend on each other. This is clear in all three plots. The high impedance phases match with high reactive components before settling into steady states. This coupling suggests an equivalent circuit model. It includes series resistance and parallel capacitance. Proteinoid swelling or ion migration may influence this model. Time-resolved impedance spectroscopy is useful for predicting biointerface performance.

The three figures show that impedance changes a lot at first (0–5000 s). Then, it stabilizes and has fewer fluctuations. This information can help optimize aragonite-proteinoid devices used in neuromorphic or sensing applications. The stats show high standard deviations in early data and low medians later on. This suggests that outside factors, like humidity and temperature, might be adjusted to lengthen the activation window. More cyclic studies are needed to check for reversibility and longevity.

At 1000 Hz, the impedance parameters for the aragonite–proteinoid system exhibit a clear and consistent pattern, as shown in Table 6. Both the real impedance component (*Z′*) and the negative imaginary impedance component (−*Z*″**) decay exponentially, with fitted decay rates of *b* = 0.0003 and time constants of approximately 3333 s. This similarity suggests that the resistive and reactive relaxation processes at the organic–inorganic interface occur concurrently. The fitted baselines indicate stabilization at *c* = 19.45 Ω for *Z′* and *c* = −0.05 Ω for −*Z*″**, implying transition to a low-resistance regime. In contrast, the capacitance (*C*_*s*_) shows nonmonotonic behavior and does not follow a simple exponential decay. Instead, it exhibits episodic spikes that result in a low mean value of 0.0001 F, but with high variability—its standard deviation is approximately 100% of the mean.

**Table 6:** Analytical Summary of Time-Dependent Impedance Parameters at 1000 Hz for the Aragonite-Proteinoid System over 17,016 Data Points (Approximately 4.7 h at 1 Hz Sampling)^a^

parameter	*Z′* (Ω)	–*Z*″** (Ω)	Cs (F)
Exponential decay fit parameters (*y* = *a* * exp(−*b* * *t*) + *c*)			
*a*	285.38	39.12	
*b*	0.0003	0.0003	
*c*	19.45	–0.05	
Summary statistics			
mean	82.6196	7.2403	0.0001
standard deviation	73.4504	9.7928	0.0001
minimum	29.7579	–1.0985	–0.0017
maximum	415.9044	43.6973	0.0026
median	40.5147	1.7778	0.0001
Correlation			
correlation (*Z′* and −*Z″*)	0.9874		

a The exponential decay fits for *Z′* (*a* = 285.38, *b* = 0.0003, *c* = 19.45) and −*Z*″** (*a* = 39.12, *b* = 0.0003, *c* = −0.05) show quick initial drops. The time constant is about τ ≈ 3333 s. After this, values stabilize to a baseline. This behavior shows interface activation and equilibration. The similar decay rates (b) point to linked resistive-reactive dynamics. Capacitance (Cs) varies in episodes and doesn’t show a steady decline. Its spikes, reaching a max of 0.0026 F, suggest sudden events like ion restructuring instead of a smooth decay. High standard deviations compared to means (e.g., 89% for *Z′*, 135% for −*Z*″**) show instability in early phases. The strong correlation of 0.9874 between *Z′* and −*Z″* indicates a semicircular Nyquist trajectory. This suggests a simple RC circuit model. Deviations, or negative minima, may stem from inductive artifacts or diffusion. These results highlight how time affects degradation rates. This information helps improve biointerfaces for better stability in neuromorphic applications.

The strong correlation between *Z′* and −*Z*″** (*r* = 0.9874) confirms a unified RC-like behavior in the equivalent circuit. Deviations at the minima—such as the −1.0985 Ω observed for −*Z*″** —may result from inductive effects or measurement noise. These observations underscore the need for more advanced models to accurately capture degradation kinetics in bioelectronic systems.

Figure 24 illustrates the temporal evolution of these parameters. In subfigure (a), *Z′* exhibits a rapid initial drop toward equilibrium, consistent with improved charge transfer following interface activation. Subfigure (b) shows −*Z*″** relaxing on a similar time scale, indicative of capacitive discharge processes. Subfigure (c) presents the phase angle over time, stabilizing around 2°, with a mean value of 3.46°. This suggests reduced polarization losses over time and identifies an initial phase (0–5000 s) marked by high variability. Beyond this point, the system approaches steady-state resistivity, which is crucial for assessing long-term reliability of biointerfaces.

**Figure 24:**
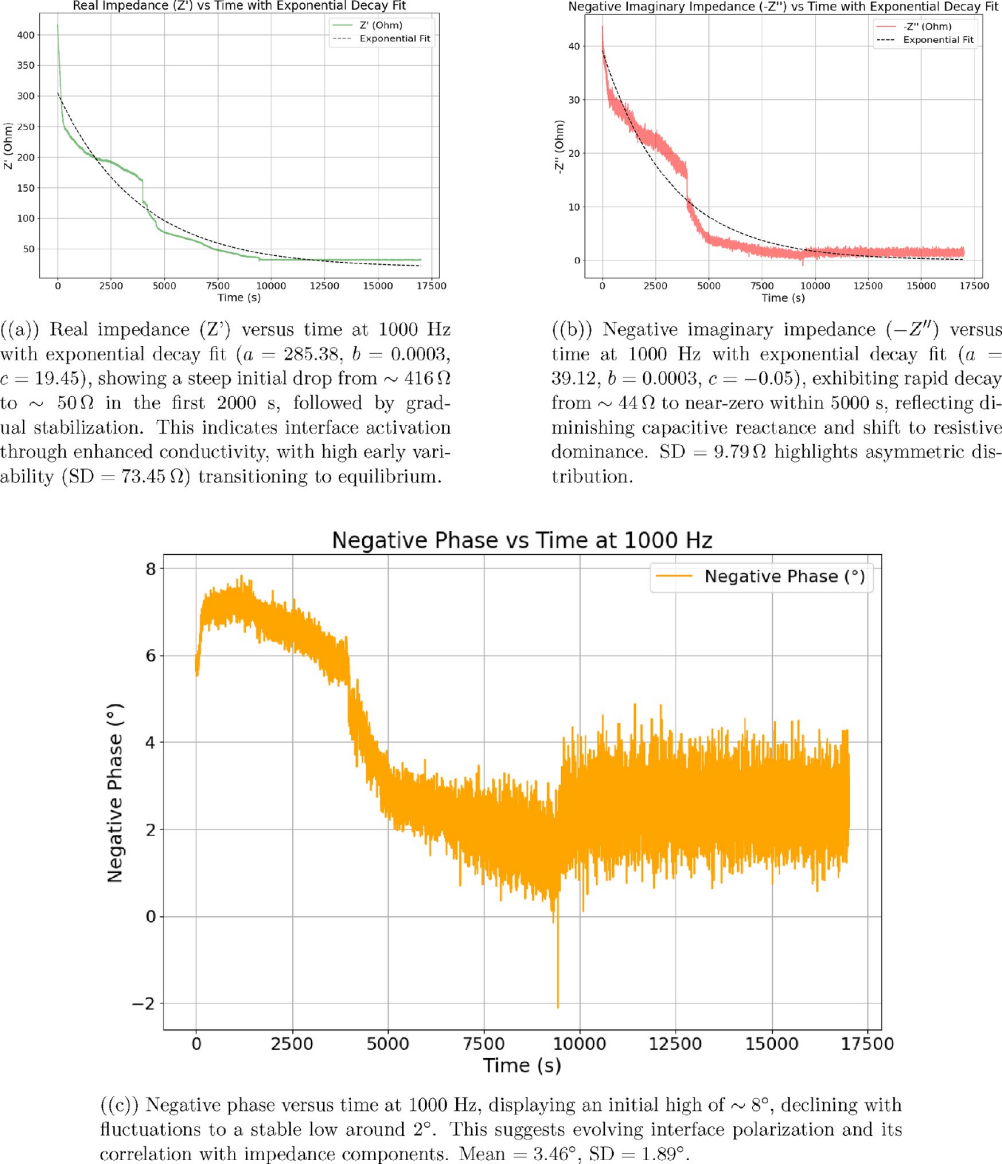
Time-series analysis of impedance parameters at 1000 Hz for the aragonite–proteinoid system, capturing activation and stabilization dynamics over ∼4.7 h. (a) *Z′* decay fit; (b) −*Z*″** decay fit; (c) phase evolution. Overall, the trends suggest initial reactive transients yielding to resistive equilibrium, with strong *Z′*––*Z*″** correlation (*r* = 0.9874), relevant for modeling bioelectronic interface behavior.

Figure 25 presents statistical distributions and interparameter relationships. Subfigures (a) through (c) reveal skewed histograms: *C*_*s*_ (a) is clustered near zero with intermittent high-capacitance events, *Z′* (b) shows a concentration at poststabilization values below 50 Ω, and −*Z″* (c) peaks at low reactance values. In all cases, the median is lower than the mean due to early high-value outliers. The Nyquist plot in subfigure (d) forms a depressed semicircle, reinforcing the observed RC behavior and strong correlation. The compression of the trajectory at low impedances suggests diffusion-limited charge transport in the later stages of the experiment. This provides a compelling visual confirmation for the equivalent circuit model and supports its application in predicting long-term stability of neuromorphic and bioelectronic interfaces.

**Figure 25:**
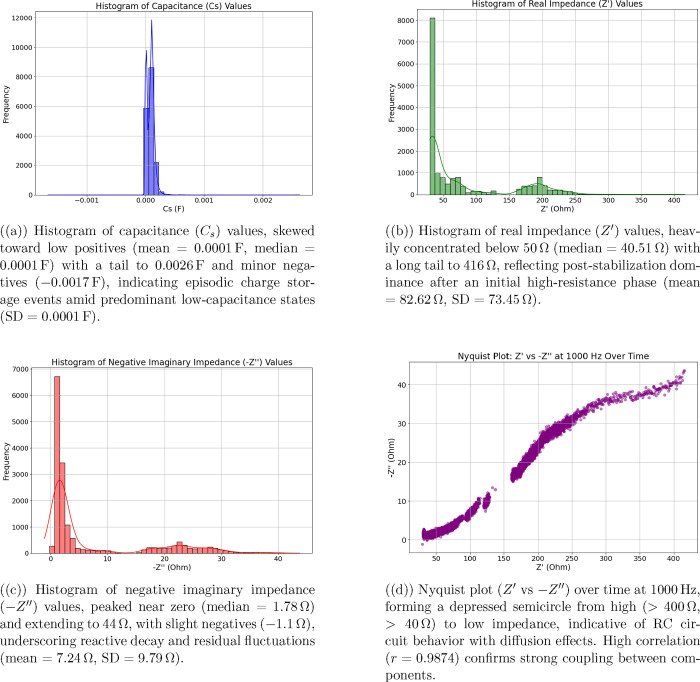
Distribution and relational analysis of impedance parameters at 1000 Hz, providing statistical and visual insights into variability and interdependencies. (a) *C*_*s*_ histogram; (b) *Z′* histogram; (c) −*Z*″** histogram; (d) Nyquist plot. Collectively, the histograms reveal skewed distributions toward equilibrium states, while the Nyquist representation supports equivalent circuit modeling and predictive degradation analysis for biointerfaces.

### Proposed Equivalent Circuit Model for Electrochemical Logic

The impedance behavior in Figure 26 shows that aragonite–proteinoid assemblies have two different electrochemical regimes. Each regime has a unique linear relationship between the imaginary and real components of impedance. This behavior suggests that the charge transport mechanism relies on both resistive and diffusive pathways. These pathways arise from the layered micro- and nanoscale structure of the aragonite–proteinoid matrix. Proteinoids form dense, porous aggregates. Their internal channels create complex ionic pathways, similar to disordered solid-state electrolytes. In the high-frequency range, the nearly straight slope of the Nyquist response reflects Warburg-type diffusion impedance. It is usually expressed as

$$Z_{W}(\omega )=\sigma (1-j)\omega^{-1/2}$$ 28

where σ is the Warburg coefficient and ω is angular frequency. This term comes from charge carriers moving through semi-infinite diffusion in complex pore networks. This is just like the structure seen in proteinoids. When combined with the solution resistance *R*_*s*_, the total impedance

$$Z(\omega )=R_{s}+Z_{W}(\omega )$$ 29

produces the characteristic 45° diffusion line seen in the early part of Figure 26. The proteinoid structure affects ion mobility, accumulation, and delay. Together, these factors shape the frequency-dependent phase response. This behavior is key for neuromorphic function. It works like diffusion-limited charge storage, which mimics the memory and filtering seen in biological ion channels and dendritic spines.

**Figure 26:**
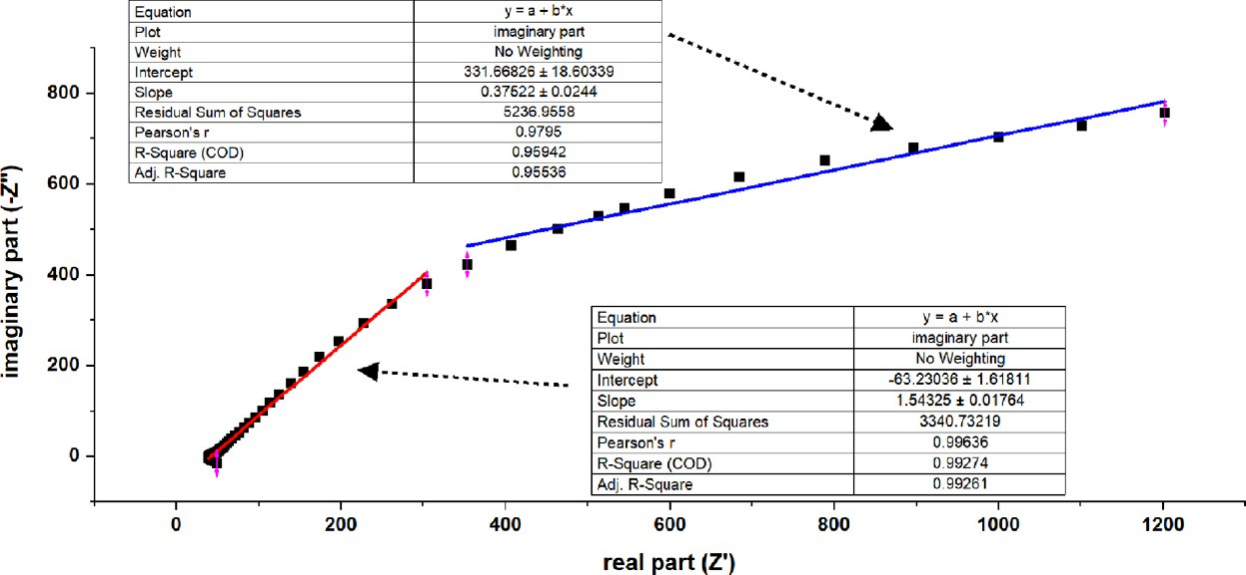
Linear fitting of the Nyquist plot showing the imaginary part of the impedance (−*Z*″**) as a function of the real part (*Z′*). The experimental data points are shown as black squares. Two distinct linear regions were fitted by ordinary least-squares regression using the model *y* = *a* + *bx*. In the low–impedance region (red line, left segment), the fit yields an intercept of *a* = −63.23 ± 1.62 Ω and a slope of *b* = 1.543 ± 0.018, with a residual sum of squares of 3340.7, Pearson correlation coefficient *r* = 0.996, and *R* ^2^ = 0.993 (adjusted *R* ^2^ = 0.993). In the high–impedance region (blue line, right segment), the intercept is *a* = 331.67 ± 18.60 Ω and the slope is *b* = 0.375 ± 0.024, with a residual sum of squares of 5237.0, Pearson *r* = 0.980, and *R* ^2^ = 0.959 (adjusted *R* ^2^ = 0.956). The change in slope between the two regimes indicates a transition in the dominant impedance contribution as *Z′* increases: the steeper low-*Z′* segment reflects a stronger increase of −*Z*″** with *Z′*, whereas the shallower high-*Z′* segment suggests a different transport or interfacial mechanism controlling the impedance at larger characteristic length or time scales.

At lower frequencies (see the right segment of Figure 26), the impedance moves away from the ideal Warburg line. It enters a region that is better explained by a distributed RC network. Proteinoid aggregates have many protonation sites, zwitterionic side chains, and microcapacitive interfaces. Together, these features act like a big group of parallel RC elements. A minimal kinetic model can be written as

$$Z(\omega )=R_{s}+\frac{R_{\mathrm{ct}}}{1+j\omega R_{\mathrm{ct}}C_{\mathrm{eff}}}+Z_{W}(\omega )$$ 30

where *R*_ct_ is the slow interfacial (redox-like) charge-transfer resistance, and *C*_eff_ represents the effective double-layer or dipolar capacitance from the proteinoid network. The absence of a complete semicircle shows a gradual slope change. In the low-impedance area, *b* ≈ 1.54, and in the high-impedance area, *b* ≈ 0.38. This suggests that capacitive charging grows stronger as diffusion slows and ions build up at local binding sites. This behavior shows fractional-order kinetics and constant-phase-element (CPE) responses. These occur naturally in complex organic materials with varied relaxation times. A mixed diffusion–capacitive response is important for neuromorphic operation. The proteinoid shows long-tailed responses, memory-like impedance histories, and signal attenuation that depends on frequency. This is similar to how synaptic filtering works. The RC–diffusion model explains the two-slope behavior seen in experiments. It shows how proteinoids store, integrate, and transform electrical signals. This process aligns with how artificial synaptic and dendritic elements function.

## Discussion

### Hierarchical Self-Assembly Mechanisms

The aragonite-proteinoid system shows a complex self-assembly process (Figure 27). Isolated proteinoid microspheres form complex three-dimensional networks. This happens due to the influence of aragonite surfaces. Proteinoids form when amino acids heat up and stick together. In water, they create hollow microspheres. When these microspheres meet calcium carbonate, like aragonite, they turn into tiny spheres about 110 nm wide.^23^ This process is like how cells form naturally, like protocells or lipid vesicles, where energy drives the assembly. In this case, hydrophobic interactions help minimize free energy. The process starts with nucleation in supersaturated solutions. Aragonite acts as a stabilizing site for these microspheres, helping them form. This mechanism shows how complex structures can form without biological processes. It gives hints about how prebiotic evolution happened. It also shows how entropy-driven aggregation and electrostatic interactions play a role.^56^

**Figure 27:**
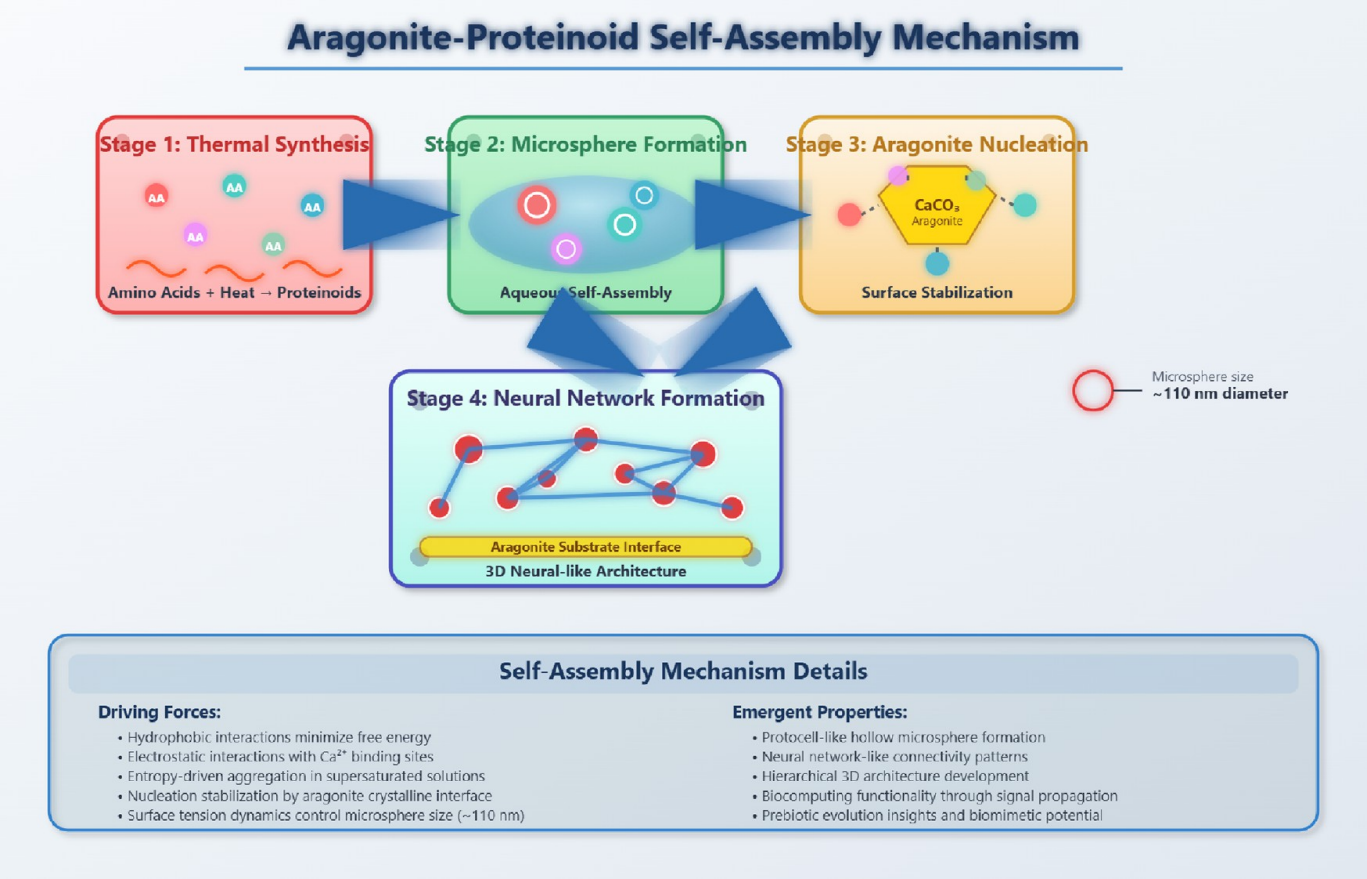
A diagram illustrates the hierarchical self-assembly process in the aragonite–proteinoid system. It outlines how isolated amino acids transform step by step into complex 3D neural-like structures. The process begins with Stage 1: Thermal Synthesis, where amino acids (represented as colored “AA” symbols) undergo heating and combine to form proteinoids. This mimics prebiotic thermal condensation mechanisms. In Stage 2: Microsphere Formation, aqueous self-assembly leads to the creation of hollow microspheres. Hydrophobic and electrostatic interactions cooperate to form spherical structures that encapsulate internal volumes, resembling protocells. Stage 3: Aragonite Nucleation follows, wherein microspheres interact with calcium carbonate (CaCO_3_) in its aragonite polymorph. This interaction stabilizes the microsphere surfaces and produces nucleation sites for further organization. Aragonite crystals, shown as hexagonal shapes, guide templating and polymorphic control. Stage 4: Neural Network Formation reveals the development of 3D microsphere networks on the aragonite substrate. These exhibit budding, asymmetric division, and branching morphologies reminiscent of biological neural structures, potentially supporting signal propagation and information processing. Microspheres are annotated with an average diameter of approximately 110 nm, emphasizing nanoscale precision. The lower panel presents the Self-Assembly Mechanism, detailing the driving forces: hydrophobic interactions minimizing Gibbs free energy, electrostatic attraction between charged residues and Ca^2+^ ions at binding sites, entropy-driven aggregation in supersaturated environments, nucleation stabilization at aragonite crystalline interfaces, and surface tension dynamics regulating microsphere sizes (∼1–3 μm). Emergent properties include: (i) protocell-like hollow microsphere formation for compartmentalization; (ii) neural-network-like connectivity for hierarchical organization; (iii) biocomputing potential through electrical or chemical signal propagation; and (iv) relevance to prebiotic evolution, with biomimetic implications for bioelectronics and neuromorphic system design. This diagram highlights abiotic routes to complexity and compares them to biological self-assembly processes such as actin polymerization and viral capsid formation. It emphasizes energy minimization and kinetic factors like Ostwald ripening and surface adsorption in shaping emergent structure.

### Electrochemical Optimization and Pulse Amplitude Effects

The electrochemical performance in the aragonite-proteinoid system shows a biphasic response. It peaks at a 0.2 V pulse amplitude. After that, efficiency drops. This decline happens because the system shifts from mixed redox processes to mainly oxidative ones. This optimization likely comes from activating electroactive sites on proteinoid surfaces at moderate amplitudes. This boosts charge transfer without causing denaturation or passivation.^57,58^ Yet, at higher amplitudes, overoxidation can deactivate these sites. This leads to more impedance and less reversibility. We see this in serotonin-incorporated proteinoids, where amplitude affects spiking patterns.^59^ Mechanically, this is like how biological ion channels work. Voltage thresholds balance excitation and inhibition. The transition indicates that redox-active amino acid residues become saturated. This insight helps design tunable bioelectrodes for sensing applications.

### Boolean Logic Implementation and Biocomputing Potential

Seven Boolean logic gates (AND, OR, XOR, NAND, and others) have been successfully created using electrochemical responses in proteinoid ensembles. This shows their potential in biocomputing. They can also convert analog signals to digital by using spiking thresholds. Gates work by reading current spikes as binary outputs. The natural electrical activity of proteinoids allows for optical or electrical changes during logic operations.^60,61^ This is like new protein designs for logic in synthetic biology but is simpler. The key point is in neuromorphic computing. Here, proteinoid devices may handle fault-tolerant tasks in mixed systems. However, challenges like scalability and noise tolerance still exist for real biocomputers.

### Frequency-Dependent Electrochemical Dynamics

Frequency-dependent dynamics show a power-law scaling in the aragonite-proteinoid system. The best performance occurs in the 30–50 Hz range. This suggests electrochemical saturation and low frequency sensitivity outside this range. Acoustic or electrical excitations create impulse patterns linked to specific frequencies. This hints at resonant modes that boost conductivity. This weak dependence shows that capacitance plays a big role at high frequencies, which limits charge storage. Yet, it also allows for temporal coding in bioelectronics. For device design, this means we can develop frequency-tuned sensors or oscillators. We can look to kombucha-proteinoid hybrids, as their composition affects selectivity. This could lead to more adaptable interfaces in wearable technology.

### Spontaneous Oscillatory Behavior and Autonomous Function

Autonomous oscillations lasted 25 h with a periodicity of 76.81 s in the aragonite-proteinoid system. These oscillations remind us of biological circadian rhythms. They come from limit cycle dynamics in phase space. Proteinoid microspheres show bursts that might come from ion gradients and swelling cycles. These bursts are like how neurons work. Phase trajectories reveal stable patterns, like Hopf bifurcations found in biological oscillators. This suggests they could be useful in self-sustaining bioelectronics. But, outside changes might disrupt their independence, much like desynchronization seen in some neural disorders.

### Electrochemical Stability and Degradation Kinetics

A 99.8% drop in electrochemical variance over 100 cycles shows that proteinoid materials degrade. This likely involves protein denaturation, surface passivation, and disruption at aragonite boundaries. Mechanisms include the breakdown of amino acid chains by heat or oxidation. This leads to changes and loss of electroactivity. It is like how polymers interact with proteins. Degradation products can change structure. This shows why stabilizers are needed. They help reduce unfolding from entropy and improve the lifespan of devices meant for repeated use.^62,63^

### Impedance Characteristics and Equivalent Circuit Modeling

Impedance at proteinoid-aragonite interfaces shows complex behavior that depends on frequency. The changes over time connect resistive and capacitive elements. This allows for predictive modeling using Randles circuits. High correlations point to ion diffusion-limited processes. These are shaped by polymorphic structures such as Glu-Phe-Asp networks.^64^ This knowledge helps in creating hybrid models for biointerfaces. Here, capacitance shows charge storage at the organic–inorganic boundary. This is key for improving sensor sensitivity, even with nonideal behaviors.

### Comparison with Biological Interconnected Microsphere Networks

Proteinoid networks mimic biological neural systems. They show oscillations, excitability, and connectivity. Yet, they lack synaptic plasticity and genetic regulation. Proto-neural ensembles mimic neuron spiking, using simpler, nonliving processes.^65^ They are less complex than mammalian networks, where transcription factors guide differentiation. Still, proteinoids share similarities in their emergent computation, making them models for early cognition. Table 7 shows the key findings of this study and the ongoing challenges in biocomputing.

**Table 7:** Breakthroughs Achieved in the Study of the Aragonite-Proteinoid System and the Remaining Unsolved Challenges in the Field of Biocomputing

breakthroughs in the aragonite-proteinoid system	unsolved challenges in biocomputing
**hierarchical self-a** **ssembly mechanisms**: demonstrated how aragonite surfaces guide proteinoid microspheres into complex 3D networks, mimicking biological processes and providing insights into prebiotic evolution through thermodynamic and kinetic factors.	stability issues, such as protein denaturation and surface passivation, limit long-term functionality and require advanced encapsulation techniques.
**neuron-reminiscent branched morphology– budding and network formation**: observed asymmetric division and daughter sphere formation, creating neural network-like architectures for signal propagation, analogous to biological microsphere budding and division.	lack of true synaptic plasticity and genetic regulation hinders advanced learning and memory capabilities compared to biological neural systems.
**electrochemic** **al optimization and pulse amplitude effects**: identified optimal performance at 0.2 V pulse amplitude, revealing mechanisms of electroactive site activation and biphasic redox behavior.	degradation kinetics over cycles, including a 99.8% reduction in variance, pose challenges for repeated-use devices and durability in real-world applications.
**Boolean logic implementation and biocomputing potential**: successfully implemented seven Boolean logic gates via electrochemical responses, enabling analog-to-digital conversion for neuromorphic computing.	scalability and noise tolerance remain barriers, as current systems struggle with complex computations and environmental interference.
**frequency-** **dependent electrochemical dynamics**: uncovered power-law scaling and optimal frequency range (30–50 Hz), suggesting applications in bioelectronic device design with resonant modes.	weak frequency dependence and electrochemical saturation limit adaptability in high-frequency operations and dynamic environments.
**spontaneous oscillatory behavior and autonomous function**: achieved 25-h autonomous oscillations with 76.81-s periodicity, comparable to biological rhythms, driven by limit cycle dynamics.	reproducibility challenges due to synthesis variability affect consistent performance and standardization for practical deployment.
**impedance characteristics and equivalent circuit modeling**: analyzed complex impedance behavior and correlations between resistive/capacitive elements for predictive modeling of biointerfaces.	technical barriers, such as impedance mismatches at organic–inorganic interfaces, complicate integration into hybrid bioelectronic systems.
**comparison with biological interconnected microsphere networks**: highlighted similarities in oscillations, excitability, and connectivity, positioning proteinoids as models for primitive cognition.	reduced complexity and absence of higher-order biological features restrict the emulation of advanced neural behaviors like adaptive learning.
**applications and technological implications**: proposed uses in autonomous sensing, energy harvesting, biocomputing, and neuromorphic technologies, leveraging biocompatibility and low-cost synthesis.	overall limitations in longevity, fault-tolerance, and integration with existing technologies hinder transition from lab prototypes to commercial applications.

### Functional Limitations Compared to Biological Neural Systems

While morphological similarities exist between aragonite-proteinoid microsphere networks and biological neural architectures, critical functional distinctions must be emphasized:

#### Lack of Active Signal Propagation

Unlike neurons that propagate action potentials via voltage-gated ion channels, we have not demonstrated directed signal transmission between proteinoid nodes. The observed oscillations appear localized rather than propagating waves.

#### Absence of Synaptic Mechanisms

No evidence of chemical or electrical synaptic transmission has been observed. The microsphere connections represent physical bridges, not functional synapses with neurotransmitter release, receptor binding, or synaptic plasticity.

#### No Adaptive or Learning Behavior

Biological neurons exhibit synaptic strengthening (LTP) and weakening (LTD) based on activity patterns. Our system shows degradation rather than potentiation, and no activity-dependent modification of responses has been demonstrated.

#### Missing Refractory Dynamics

Neuronal action potentials are followed by absolute and relative refractory periods due to ion channel inactivation. No analogous refractory behavior has been observed in proteinoid oscillations, which show continuous periodicity without activity-dependent timing changes. These distinctions indicate that aragonite-proteinoid systems represent primitive electrochemical oscillators with interesting computational potential, but should not be equated with functional neural networks.

## Conclusions

This study of aragonite-proteinoid microstructures shows a complex bioelectronic platform. It can process information on its own using new electrochemical behaviors. Hierarchical self-assembly works! It shows how single microspheres create complex, neural network-like structures. This provides strong proof that mineral-organic interfaces can be the basis for new computing systems. The best electrochemical conditions at a 0.2 V pulse amplitude, combined with full Boolean logic operations, show that these materials are effective for biocomputing. They connect biological and synthetic information processing systems. The discovery of sustained autonomous oscillations with a biological-like rhythm is a big breakthrough. It shows that these hybrid systems can create ongoing electrical signals without any outside help. Aragonite-proteinoid microstructures are promising for next-generation bioelectronic devices. Their tunable electrochemical properties and complex impedance characteristics support this potential. The power-law scaling relationship helps predict how devices respond to frequency. This framework allows us to improve performance and reduce energy use, which is crucial for real-world applications. The observed degradation rates show limits in operational lifetime. Yet, the fundamental principles in this work lay a strong foundation for future advances in mineral-organic computing platforms. Proteinoid budding and biological microsphere budding and division share striking similarities. These connections may reveal how information processing began in prebiotic systems. They could also lead to new biomimetic technologies. As we move toward advanced bioelectronic systems, aragonite-proteinoid microstructures offer exciting potential. They blend materials science, unconventional computing, and bioinspired design. This could change how we do autonomous sensing, adaptive computing, and energy harvesting in places where silicon-based tech falls short.

## Data Availability

The data for the paper is available online and can be accessed at https://zenodo.org/records/15877432.
